# Predictive Design of Fulvalene-Bridged Bimetallic Molecular Solar–Thermal Fuels

**DOI:** 10.1021/jacsau.6c00919

**Published:** 2026-08-16

**Authors:** Gaurab Ganguly, Leticia González

**Affiliations:** Institute of Theoretical Chemistry, Faculty of Chemistry, 27258 University of Vienna, Währinger Str. 17, 1090 Vienna, Austria

**Keywords:** molecular solar−thermal (MOST) energy storage, bimetallic complexes, molecular photoswitches, intersystem crossing, computational screening, periodic trends

## Abstract

Molecular solar–thermal (MOST) energy storage requires the simultaneous optimization of photocharging efficiency, energy storage capacity, and thermal persistence. The diruthenium fulvalene complex (Fv)­Ru_2_(CO)_4_ remains the only organometallic MOST fuel that supports a fully closed photothermal cycle, enabled by triplet-surface mediated photocharging and singlet-surface mediated thermal discharge. Isoelectronic metal substitution typically disrupts this closed cycle, either by quenching the essential triplet photocharging pathway or by kinetically trapping the high-energy isomer—yet these failures have remained without a unifying mechanistic rationale, and whether viable compositions exist beyond (Fv)­Ru_2_(CO)_4_ has been unknown. Here, we introduce a dual-gate framework that elevates metal-pair selection from an empirical choice to a predictive and rational design parameter. The framework defines two complementary filters: a ground-state *Storage*–*Stability Gate*, in which stored free energy and the thermal back-reaction barrier are intrinsically coupled, and an excited-state *Photo Gate*, requiring Franck–Condon-proximal access to the isomerizing triplet. Screening a 21-member library of Group 6/Group 8 homo- and heterobimetallic dimers with these two filters identifies (Fv)­OsW­(CO)_5_ as a previously unrecognized, synthetically accessible MOST candidate. This proof-of-concept demonstrates that viable MOST candidates can be found beyond the (Fv)­Ru_2_(CO)_4_ benchmark through rational design rather than empirical substitution. The framework simultaneously explains why isoelectronic substitution has historically failed, delineating viable and nonviable regions of bimetallic design space and providing transferable, mechanism-guided principles for MOST design.

## Graphical abstract

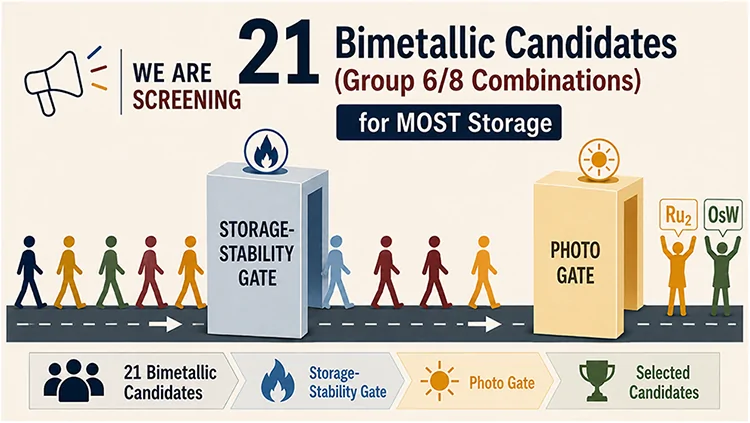

## Introduction

Efficient storage of solar energy remains a central challenge for sustainable energy technologies, because sunlight is abundant yet inherently intermittent and global demand continues to rise.^1,2^ Molecular solar–thermal (MOST) systems offer an intriguing and conceptually direct solution by encoding photon energy as chemical strain in a metastable photoisomer and releasing it as heat on demand.^3,4^ As a fully molecular, closed-cycle approach,^5^ MOST strategies can complement established solar-to-chemical routes such as water splitting^6^ or carbon dioxide reduction^7,8^ as well as downstream hydrogen storage,^9–11^ which are often constrained by phase changes, kinetic bottlenecks, and the handling of gaseous intermediates.

In a prototypical MOST cycle, visible-light excitation induces photoisomerization from a lower-energy isomer (LEI) to a photocharged higher-energy isomer (HEI), thereby storing solar energy as molecular strain.^3–5^ Energy is released during the thermal back-reaction (TBR), where the HEI reverts to the LEI. Viable MOST fuels must therefore satisfy three interdependent criteria: efficient photocharging under solar irradiation, a large storage free energy (Δ*G*_storage_), and kinetic persistence governed by an appropriately tuned TBR barrier (Δ*G*_TBR_^‡^). Because these objectives are rarely independently tunable, MOST discovery is inherently a multiobjective problem. Viable candidates are thus expected to lie on a narrow, Pareto-like trade-off surface between storage density and kinetic persistence, while large regions of chemical space may be fundamentally nonviable rather than merely uncharted. Consistent with this stringency, MOST development has remained largely confined to a small number of organic photoswitch families, for which only narrow substitution windows satisfy the coupled thermodynamic, kinetic, and photochemical demands.^3,4,12–18^ Transferable design rules that generalize beyond local chemical variations therefore remain scarce.^3,4^

Against this context of predominantly organic MOST systems, the fulvalene-bridged diruthenium complex (Fv)­Ru_2_(CO)_4_ stands as a rare exception: it remains the only organometallic MOST fuel—and one of the few thermally reversible photoswitches in the organometallic domain—demonstrated to support durable closed-loop cycling (Δ*G*_storage_ ≈ 19.8 kcal mol^–1^). This behavior is enabled by a distinctive spin-gated intersystem crossing (ISC) mechanism that accesses a triplet-mediated photocharging pathway coupled to singlet-surface thermal discharge (Figure 1a).^19,21–23^ This behavior is sharply metal-selective. Isoelectronic Group 8 congeners fail in distinct ways: (Fv)­Fe_2_(CO)_4_ undergoes destructive photodecarbonylation,^22,24^ (Fv)­Os_2_(CO)_4_ forms a kinetically trapped HEI that resists thermal reversion even at ca. 275 °C,^25^ and the heterobimetallic (Fv)­RuFe­(CO)_4_ does not photocharge.^25^ Taken together, these observations indicate that only (Fv)­Ru_2_(CO)_4_ simultaneously achieves efficient photocharging and viable thermal energy release, whereas its congeners fail in at least one of these respects. Consequently, (Fv)­Ru_2_(CO)_4_ serves as the benchmark organometallic MOST system against which new candidates may be assessed—yet the physical origins of its uniqueness, and of its congeners’ failures, have not previously been unified into transferable design principles.

**Figure 1:**
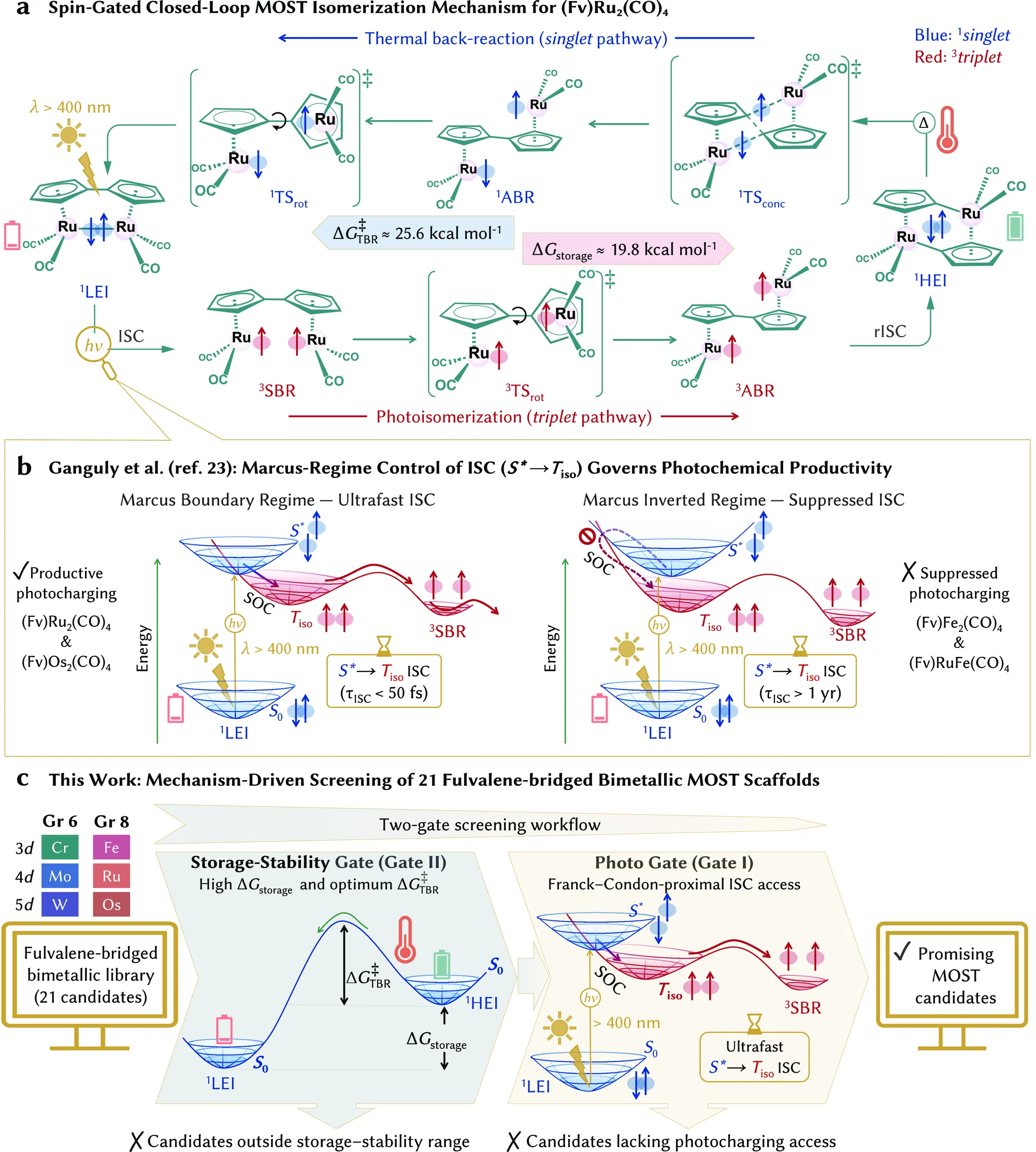
Spin-gated MOST mechanism and dual-gate screening strategy. (a) In (Fv)­Ru_2_(CO)_4_, photoexcitation of the lower-energy isomer (LEI) populates a bright singlet state (S*), followed by intersystem crossing (ISC) to the isomerizing triplet state (*T*_iso_). On the triplet surface, metal–metal bond homolysis generates the triplet *syn* biradical (^3^SBR), which undergoes *syn*-to-*anti* biradical conversion (^3^SBR → ^3^ABR) en route to the higher-energy isomer (HEI). Reverse ISC restores the singlet surface to complete photocharging. The thermal back-reaction (TBR) proceeds exclusively on the singlet surface via concerted C–C/M–C bond reorganization followed by C–C torsional relaxation.^19–23^ (b) The decisive S* → *T*_iso_ transition is governed by Marcus-type alignment of the singlet–triplet crossing seam. When the seam resides near the Marcus boundary (e.g., Ru_2_, Os_2_), ISC is ultrafast and photocharging proceeds efficiently. When displaced into the Marcus-inverted regime (e.g., Fe_2_, RuFe), ISC becomes kinetically suppressed despite substantial spin–orbit coupling, thereby preventing population of *T*_iso_. Adapted from ref 23. (c) Mechanism-informed screening integrates two independent requirements: Gate I (*Photo Gate*), demanding Franck–Condon-proximal ISC access to *T*_iso_, and Gate II (*Storage*–*Stability Gate*), requiring favorable energy storage (Δ*G*_storage_) together with a kinetically viable TBR barrier (Δ*G*_TBR_). Productive MOST candidates emerge only when both gates are satisfied.

The examples above illustrate that the choice of the metal controls both photochemical access and thermal discharge. For decades, however, metals in fulvalene-bridged bimetallic complexes were chosen empirically. Only recently, we identified the ISC step as a key factor controlling photocharging (Figure 1b).^23^ Following excitation into a bright singlet manifold (S*), productive photocharging proceeds via a spin-gated cascade: Franck–Condon-proximal ISC, enabled by spin–orbit coupling, populates the isomerization-active triplet state (*T*_iso_), which relaxes via metal–metal bond homolysis into the triplet *syn* biradical (^3^SBR) reservoir, from which structural evolution through *anti* biradical (^3^ABR) proceeds toward the photocharged isomer (HEI). Crucially, the efficiency of the decisive S* → *T*_iso_ step is governed by the energetic placement of the crossing seam, according to Marcus-type behavior.^26,27^ When the seam lies near the Marcus boundary, ISC is efficient and photoisomerization proceeds (as in Ru_2_ and Os_2_ complexes); when it is displaced into the Marcus-inverted regime, ISC is kinetically hindered even with strong spin–orbit coupling, blocking photocharging (as in Fe_2_ and RuFe complexes).^23^ Marcus-type control of excited-state relaxation, including ISC and energy transfer, spans organic photosensitizers,^28^ organic mixed-valence systems,^29^ and transition-metal complexes,^30–34^ underscoring its generality. This behavior allows us to establish what we call Gate I (*Photo Gate*) as an excited-state viability criterion for MOST operation.

A complementary, transferable descriptor governing ground-state control of Δ*G*_storage_ and Δ*G*_TBR_^‡^ has not yet been formulated. While it is empirically known that metal identity reshapes the HEI stability and discharge kinetics—e.g., rendering Os_2_ kinetically trapped while Ru_2_ is functional^20,25^ —the physical origin of this selectivity has not been articulated as a design principle. As a result, candidates that are photoactive may still perform poorly due to unfavorable ground-state energetics, and yet would appear promising in the absence of a mechanistic filter. Here, we exploit the fact that bond-exchange thermochemistry intrinsically couples storage free energy to the thermal back-reaction barrier to define a narrow thermodynamic–kinetic frontier that constrains viable MOST cycling (Figure 1c). We call this requirement Gate II (*Storage*–*Stability Gate*), thereby defining large regions of bimetallic design space as intrinsically nonactionable prior to photochemical screening. Together, Gates I and II act as complementary filters that transform metal pairing from a purely compositional choice into a mechanism-guided principle for rational MOST design.

In this paper, we apply our defined two-gate framework to a 21-member library of synthetically viable fulvalene-bridged Group 6 and Group 8 homo- and heterobimetallic compounds. Because estimating reaction barriers in the ground-state is computationally less demanding than excited-state analysis, we implement first the *Storage*–*Stability* workflow to screen thermo-kinetically constrained candidates prior to evaluate the *Photo Gate* conditions. The two gates are independent, so this ordering affects only cost, not the final outcome. This strategy identifies (Fv)­OsW­(CO)_5_ as a previously unexplored heterobimetallic candidate that satisfies both mechanistic criteria, providing proof-of-concept that MOST-competent compositions exist beyond the serendipitously discovered (Fv)­Ru_2_(CO)_4_ benchmark and can be discovered through mechanism-guided design rather than that empirical metal substitution. A central contribution of this work is also the dual-gate framework itself: a transferable, mechanism-encoded approach that transforms metal-pair selection from an empirical choice into a rational design variable.

## Results and Discussion

### Library Design and Screening Strategy

To systematically probe how metal pairing governs MOST performance, we constructed a library of 21 (Fv)­MM′(CO)*_n_* complexes (*n* = 4–6), spanning all homo- and heterobimetallic combinations of Group 6 (Cr, Mo, W) and Group 8 (Fe, Ru, Os) metals. The library is defined by a 17-electron fragmentation scheme that enforces a single scissile M–M′ bond and fixes carbonyl stoichiometry by electron counting. Formal disconnection into (Cp)­M­(CO)_*x*_ fragments (*x* = 2, 3) yields 17-electron metal centers, dictating *n* = 4 for Group 8/Group 8 dimers, *n* = 6 for Group 6/Group 6 dimers, and *n* = 5 for Group 6/Group 8 heterodimers (Supporting Section S1). This electron-precise construction isolates the M/M′ combination as the primary variable controlling storage thermodynamics, kinetic stability, and photochemical accessibility.

We adopt the experimentally validated closed-loop mechanism of (Fv)­Ru_2_(CO)_4_ depicted in Figure 1a as a mechanistic template for the (Fv)­MM′(CO)*_n_* family. There, visible-light excitation of the LEI populates a singlet state that undergoes ISC into an isomerizing triplet state for photocharging, followed by exclusive singlet-surface back-reaction during thermal discharge.^20–23^ Metal pairing then controls both (i) access to ISC into the isomerizing triplet manifold and (ii) the ground-state storage–stability landscape defined by Δ*G*_storage_ and the rate-determining Δ*G*_TBR_^‡^, which together set HEI lifetime and determine whether closed-loop cycling is feasible. Accordingly, we implement the Gate II → Gate I workflow (Figure 1c), screening the library against practical persistence targets before photochemical evaluation.

### Mechanistic Overview: Why Two Gates?

The two gates encode physically independent requirements, which is why both are necessary and neither alone suffices. Gate I (labeled as *Photo Gate*) is an excited-state criterion: after being photoexcited to the bright singlet manifold (S*), the population must reach the isomerizing triplet (*T*_iso_) through a Franck–Condon-proximal crossing, and fails when that seam is displaced into the Marcus-inverted regime.^23,26,27^ Gate II (labeled as *Storage*–*Stability Gate*) is a ground-state criterion: the HEI must store useful energy (Δ*G*_storage_) behind a back-reaction barrier (Δ*G*_TBR_^‡^) that is neither too low (spontaneous discharge) nor too high (permanent trapping).

Because the descriptor governing photocharging is decoupled from the one governing storage and release, a candidate may clear one gate while failing the other. The known congeners of (Fv)­Ru_2_(CO)_4_ fail in opposite directions: (Fv)­Os_2_(CO)_4_ photocharges efficiently^23^ yet its HEI is kinetically trapped, failing Gate II,^25^ whereas (Fv)­Fe_2_(CO)_4_ and (Fv)­RuFe­(CO)_4_ cannot photocharge as their S*/*T*_iso_ crossings fall in the Marcus-inverted regime, failing Gate I.^23^ A viable fuel must clear both gates; within the present library, only (Fv)­Ru_2_(CO)_4_ and (Fv)­OsW­(CO)_5_ do so (*vide infra*).

### Gate II: Thermal Storage and Release (Ground-State Screen)

#### Bond-Exchange Model

In the (Fv)­MM′(CO)*_n_* platform, LEI ↔ HEI interconversion follows a bond-exchange picture (Figure 2a): photocharging cleaves the unique ^1^LEI M–M′ bond and, after fulvalene reorganization, forms two terminal ^1^HEI M–Cp/M′–Cp bonds. The amount of energy stored is therefore set by a simple competition: the penalty for cleaving the M–M′ bond against the reward for forming the two M–Cp bonds. To leading order, the storage free energy reflects this bond exchange

1 $$\Delta G_{\mathrm{storage}}\approx D_{\mathrm{LEI}}\left(M\text{−}M^{\prime }\right)-\left[D_{\mathrm{HEI}}\left(M\text{−}\mathrm{Cp}\right)+D_{\mathrm{HEI}}\left(M^{\prime }\text{−}\mathrm{Cp}\right)\right]+C_{\mathrm{storage}}$$

where *D*_X_(A–B) denotes the homolytic bond dissociation free energy of bond A–B in species X, and *C*_storage_ collects effectively invariant contributions (including Cp–Cp bonding within the fulvalene framework, ligand reorganization, and entropic effects). This model directly links periodic trends in Δ*G*_storage_ to the covariation of *D*_LEI_(M–M′) and *D*_HEI_(M–Cp) across the 3*d*/4*d*/5*d* metal series.

**Figure 2:**
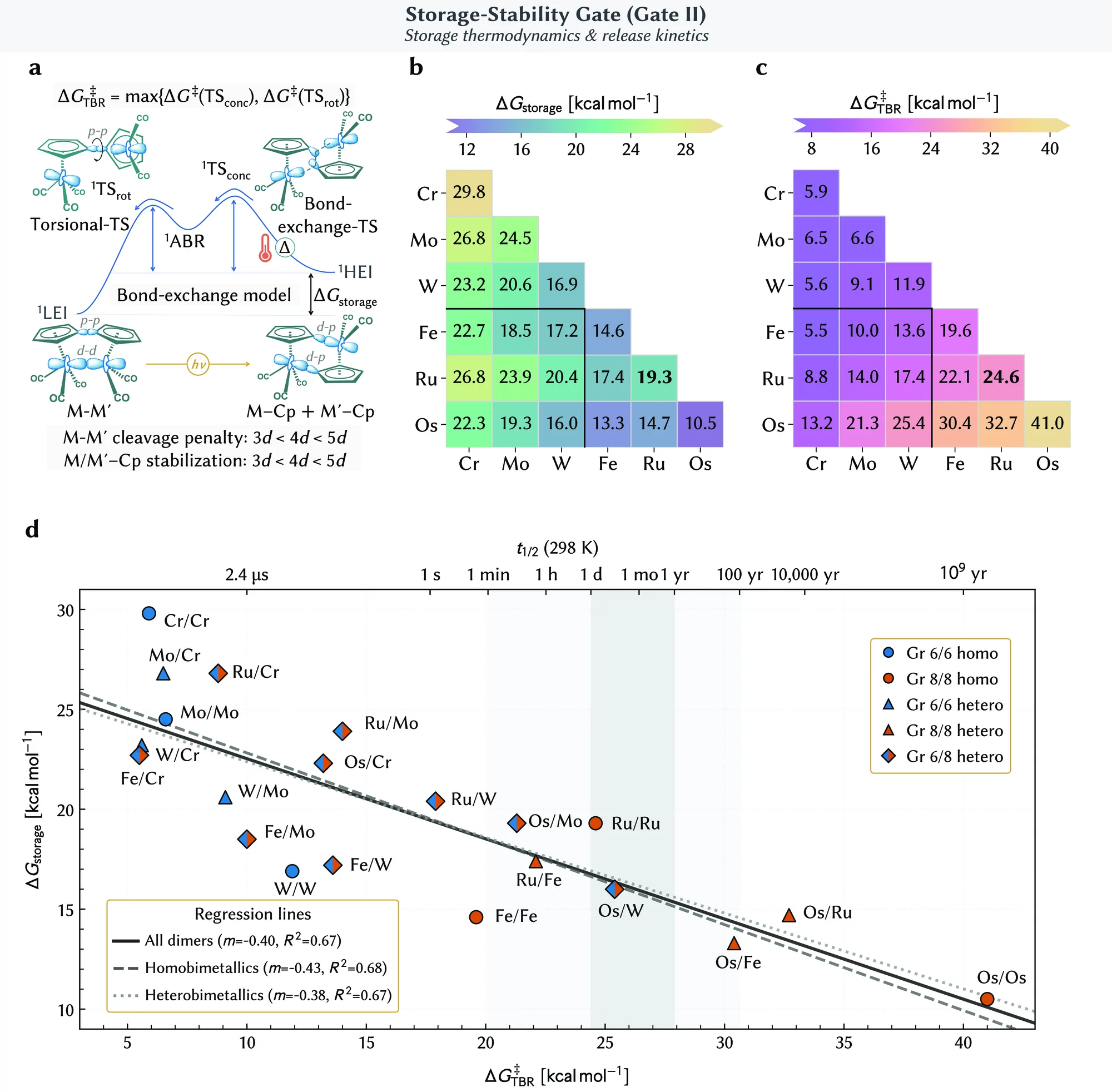
Bond-exchange energetics impose an intrinsic storage–stability frontier that constrains viable bimetallic MOST design. (a) Bond-exchange control of storage and thermal back-reaction in fulvalene-bridged bimetallic MOST dimers. Photocharging (*h*ν) converts lower energy isomer (LEI) → higher energy isomer (HEI) by cleaving the unique M–M′ bond and forming two terminal M–Cp and M′–Cp bonds; thermal discharge (Δ) reverses this exchange. Qualitative 3d/4d/5d trends are indicated. (b) Storage free energies, Δ*G*_storage_ (kcal mol^–1^), shown as a lower-triangular matrix with rows and columns ordered as Cr, Mo, W, Fe, Ru, and Os; moving to heavier metals (bottom right) lowers Δ*G*_storage_. (c) Thermal back-reaction barriers, Δ*G*_TBR_^‡^ (kcal mol^–1^), for the same set of dimers, defined as Δ*G*_TBR_^‡^ = max {Δ*G* ^‡^(^1^TS_conc_), Δ*G* ^‡^(^1^TS_rot_)} (concerted bond-exchange vs fulvalene-rotation transition state); heavier metals increase Δ*G*_TBR_^‡^ and thus kinetic stability. (d) Storage–stability trade-off: Δ*G*_storage_ versus Δ*G*_TBR_^‡^. The opposing trends in (b, c) enforce an intrinsic compromise: gains in stored energy come at the cost of kinetic persistence. An ordinary-least-squares fit, Δ*G*_storage_ ≈ *a*_all_ + *m*_all_Δ*G*_TBR_^‡^, summarizes the trend across the full data set. Symbols denote homobimetallic dimers (circles; Group 6/6 and Group 8/8), same-group heterobimetallic dimers (triangles), and cross-group Group 6/8 heterobimetallic dimers (split diamonds). Shaded regions indicate the 298 K stability window (*t*_1/2_ = 1 day–1 year, dark; *t*_1/2_ = 1 min–100 year, light; upper axis); only Ru_2_ and OsW fall within the daily use (dark) window. Frontier candidates are Ru_2_, OsW, OsFe, RuFe, and OsMo. All energies were computed at the B3LYP-D3­(BJ)/def2-TZVPP level with Stuttgart–Dresden effective core potentials for the transition metals.

The HEI → LEI thermal back-reaction proceeds exclusively on the singlet surface (Figures 1a and 2a): ^1^HEI → ^1^TS_conc_ → ^1^ABR → ^1^TS_rot_ → ^1^LEI. Here, ^1^TS_conc_ is the concerted bond-exchange transition state (simultaneous Cp–Cp bond formation and M–Cp bond cleavage) and therefore the bond-reorganization bottleneck, whereas ^1^TS_rot_ is a fulvalene dihedral-rotation transition state dominated by steric (torsional) strain; ^1^ABR denotes the singlet *anti* biradical intermediate. Because the pathway contains two distinct transition states, we define the operative barrier as the larger activation free energy (and thus the rate-determining step by definition),

ΔGTBR‡≡max{ΔG‡(TSconc1),ΔG‡(TSrot1)}

For most dimers, ^1^TS_conc_ lies slightly above ^1^TS_rot_ and is therefore rate-determining, consistent with bond reorganization in ^1^TS_conc_ versus predominantly steric torsional strain in ^1^TS_rot_ (Figure 2a). At the simplest level, because ^1^TS_conc_ involves partially breaking the same M–Cp bonds whose formation stored the energy, this barrier is approximated by

2 $$\Delta G_{\mathrm{TBR}}^{‡}\approx \alpha \left[D_{\mathrm{HEI}}\left(M\text{−}\mathrm{Cp}\right)+D_{\mathrm{HEI}}\left(M^{\prime }\text{−}\mathrm{Cp}\right)\right]+C_{\mathrm{TBR}}$$

where 0 < α < 1 captures the effective extent of M–Cp bond cleavage in ^1^TS_conc_, and *C*_TBR_ collects the remaining, largely metal-insensitive contributions (partial Cp–Cp bond formation, Cp-framework reorganization, and entropic terms). Equations 1 and 2 thus show that storage density and release kinetics are governed by the same microscopic bond strengths but enter with opposite effective signs, enforcing an intrinsic storage–stability compromise.

Full computational protocols are provided in Supporting Section S2. In short, the ground-state energy landscape was investigated using Kohn–Sham density functional theory at the B3LYP-D3­(BJ)/def2-TZVPP level,^35–38^ with Stuttgart–Dresden relativistic effective core potentials for the transition metals.^39^ This protocol was selected based on calibration against experimental storage and thermal back-reaction energetics for (Fv)­Ru_2_(CO)_4_ (Supporting Section S3). Because conventional density functionals incompletely describe long-range correlation, explicit dispersion corrections are essential in these systems, where dispersion contributions can rival or exceed covalent interactions in stabilizing weak or dative metal–metal contacts.^38,40,41^ At this level of theory, both the storage free energy (Δ*G*_storage_) and the TBR barriers (^1^TS_conc_ and ^1^TS_rot_) of the benchmark system are reproduced with good accuracy, providing a reliable foundation for analyzing relative trends across the bimetallic series. Moreover, because all 21 dimers share a common bond-exchange scaffold and ligand environment, relative free energies benefit from substantial cancellation of systematic errors; consistently, the smooth periodic trends and linear storage–stability relationship across the library (*vide infra*) indicate residual errors that are systematic rather than random.

The applicability of this protocol across the chemically diverse library is further supported by fractional occupation number weighted density (FOD) analysis^42,43^ of all 21 dimers. All systems exhibit only modest static correlation except the Cr_2_ LEI, whose energetics are therefore treated as qualitative; moreover, the frontier candidates occupy the same electronic-structure regime as the experimentally benchmarked (Fv)­Ru_2_(CO)_4_ while displaying among the lowest *N*_FOD_ values in the library (Supporting Section S4).

#### Storage Thermodynamics

The computed storage energies shown in Figure 2b reveal clear periodic trends governed by evolving metal–ligand bond strengths. Group 6 homobimetallics show a monotonic decrease in stored energy down the group: Cr_2_ (29.8 kcal mol^–1^), Mo_2_ (24.5 kcal mol^–1^), W_2_ (16.9 kcal mol^–1^). The 3*d* → 4*d* → 5*d* progression increases valence-shell diffuseness and metal–ligand overlap, strengthening *D*_HEI_(M–Cp). Scalar-relativistic effects also enhance *D*_LEI_(M–M) in the 5*d* series; however, in W_2_ this stabilization is concentrated primarily in the HEI W–Cp σ bonds, while the Cp-attenuated (fulvalene-weakened) LEI W–W interaction gains comparatively little. Consequently, Δ*D*_HEI_(M–Cp) ≫ Δ*D*_LEI_(M–M), such that LEI compensation is ineffective and the bond-exchange balance in eq 1 drives Δ*G*_storage_ downward.

Group 8 homobimetallics instead exhibit a nonmonotonic trend: Fe_2_ (14.6 kcal mol^–1^), Ru_2_ (19.3 kcal mol^–1^), Os_2_ (10.5 kcal mol^–1^). From 3*d* → 4*d*, increased orbital diffuseness strengthens both LEI and HEI; in Ru_2_, the pronounced rise in *D*_LEI_(M–M) competes effectively with the more modest increase in *D*_HEI_(M–Cp), yielding a maximum in Δ*G*_storage_. Upon entering the 5*d* row, scalar-relativistic 6*s* contraction and 5*d* radial expansion enhance covalency; in Os_2_, this preferentially stabilizes the HEI Os–Cp bonds over the LEI Os–Os interaction, so overstabilization of the HEI in eq 1 produces the lowest storage free energy in the series.

These trends mirror the intrinsic M–M bonding hierarchy established for bare dimers, where spectroscopic and high-level theoretical studies place Cr–Cr > Fe–Fe, Mo–Mo > Ru–Ru, and W–W > Os–Os in terms of bond dissociation energy, reflecting the higher formal bond orders accessible to Group 6 metals relative to their Group 8 congeners.^44–47^ A microscopic rationale emerges from *ab initio* valence-bond analyzes by Joy et al., which relate these periodic trends to the radial disposition of valence *nd* orbitals relative to the (*n* – 1)*s* ^2^(*n* – 1)*p* ^6^ semicore–shell.^48^ Compact 3*d* orbitals (lacking radial nodes) reside deep within the semicore region, so dimer formation generates strong Pauli “pressure”, favoring charge-shift bonding in which covalent–ionic resonance stabilizes an otherwise weak or even repulsive covalent structure. In contrast, additional radial nodes and relativistic expansion in the 4*d*/5*d* series shift valence density outward, relieve Pauli repulsion, and restore more conventional covalent M–M bonding. Although originally formulated for carbonyl dimers, this semicore–shell picture extends naturally to the present fulvalene complexes and rationalizes the systematic evolution of *D*_LEI_(M–M′) across the 3*d*/4*d*/5*d* blocks entering eq 1.

In contrast to the systematic periodic trends governing M–M interactions, the M–Cp bonds defining *D*_HEI_ arise from a distinct electronic balance of C­(*sp* ^*n*^) σ donation and M­(*d*) π backbonding, modulated primarily by periodic-row and relativistic effects. Accordingly, *D*_HEI_(M–Cp) does not exhibit a simple Group 6/Group 8 offset once oxidation state and ligand environment are fixed,^49^ thereby breaking the symmetry between LEI and HEI contributions and precluding simple scaling in eq 1.

Within each group, heterobimetallics validate the additivity implicit in the bond-exchange model: their storage free energies interpolate linearly between the homobimetallic limits (Figure 2b). This behavior is evident in Group 6 (CrMo: 26.8 kcal mol^–1^, CrW: 23.2 kcal mol^–1^, MoW: 20.6 kcal mol^–1^) and mirrored in Group 8 (FeRu: 17.4 kcal mol^–1^, FeOs: 13.3 kcal mol^–1^, RuOs: 14.7 kcal mol^–1^). Minor deviations from perfect linearity likely reflect asymmetric polarization and entropic contributions, but the first-order additivity encoded in eq 1 remains robust.

Cross-group heterobimetallics enact a complementary division of labor: the Group 6 partner supplies a large LEI cleavage penalty [*D*_LEI_(M–M′)], while the Group 8 partner contributes strong HEI formation rewards [*D*_HEI_(M–Cp) + *D*_HEI_(M′–Cp)]. Consequently, Δ*G*_storage_ again follows an approximately additive trend between the corresponding homobimetallics (Figure 2b). Ru (4*d*) offers the optimal balance in Group 8, providing strong LEI M–M′ bonding to sustain energy density without the excessive HEI stabilization that undermines storage in some 5*d* congeners. Accordingly, Ru-based cross-group dimers such as RuMo (23.9 kcal mol^–1^) and RuW (20.4 kcal mol^–1^) outperform their Fe analogues (FeMo: 18.5 kcal mol^–1^; FeW: 17.2 kcal mol^–1^) yet avoid the overstabilization characteristic of Os analogues (OsMo: 19.3 kcal mol^–1^; OsW: 16.0 kcal mol^–1^). As discussed below, the same bond-strength lever that optimizes Δ*G*_storage_ also controls the thermal back-reaction barrier (Δ*G*_TBR_^‡^) via the M–Cp-dominated expression in eq 2. Notably, the same relativistic HEI overstabilization that lowers Δ*G*_storage_ for the heavy 5*d* metals also accompanies their larger atomic mass, so both effects act in the same direction on a per-mass basis; the resulting gravimetric storage energies are analyzed in Supporting Section S5.

#### Release Kinetics

The thermal back-reaction barrier, Δ*G*_TBR_^‡^, governs both HEI persistence and on-demand discharge. The resulting barriers (Figure 2c) show a simpler, more direct dependence on metal identity than Δ*G*_storage_, with markedly less scatter across the library. Whereas the storage energy reflects a competition between LEI M–M′ and HEI M–Cp bonding, Δ*G*_TBR_^‡^ depends primarily on partial cleavage of the two HEI M–Cp bonds in ^1^TS_conc_. Because the compensating LEI term is absent in eq 2, Δ*G*_TBR_^‡^ follows a largely monotonic trend: as M–Cp bonds strengthen down each group, the HEI well deepens and the barrier increases.

Accordingly, the periodic 3*d* → 4*d* → 5*d* and scalar-relativistic trends that shape the storage landscape appear here in amplified form. Down Group 6, barriers rise from 5.9 to 6.6 to 11.9 kcal mol^–1^ for Cr_2_, Mo_2_, and W_2_, respectively. Down Group 8, the progression is even steeper: Fe_2_ (19.6 kcal mol^–1^), Ru_2_ (24.6 kcal mol^–1^), Os_2_ (41.0 kcal mol^–1^), where scalar-relativistic effects in Os_2_ impose a particularly large penalty for Os–Cp cleavage. This strengthening can be rationalized by enhanced σ-donation and M → C π backbonding, which progressively increase covalent character along the 3*d* → 4*d* → 5*d* series and are further reinforced by relativistic effects, rather than reflecting a simple Group 6/Group 8 dichotomy.^49^

Within-group heterobimetallic barriers interpolate nearly linearly between the homobimetallic limits (Figure 2c), consistent with approximately additive M–Cp bond strengths. Fe partners yield labile HEIs (FeW: 13.6 kcal mol^–1^), Os partners give protected systems (OsW: 25.4 kcal mol^–1^), and Ru-based dimers occupy a balanced regime (RuW: 17.4 kcal mol^–1^) where HEIs are storable yet releasable.

For the (Fv)­Ru_2_(CO)_4_ benchmark, our protocol assigns ^1^TS_conc_ as the rate-determining step, with a barrier of 24.6 kcal mol^–1^ that matches the experimental ^1^TS_conc_ barrier,^21^ while correcting an earlier GGA-based attribution of the TBR rate-determining step to ^1^TS_rot_.^20^ This assignment holds for 18 of the 21 dimers. The three exceptions—OsW (24.8 vs 25.4 kcal mol^–1^), OsMo (19.0 vs 21.3 kcal mol^–1^), and RuW (17.4 vs 17.4 kcal mol^–1^)—instead exhibit a marginally higher or essentially identical ^1^TS_rot_ barrier. These inversions arise from a slight stabilization of ^1^TS_conc_ rather than from any anomalous elevation of ^1^TS_rot_, which lies consistently 5–7 kcal mol^–1^ above the open-shell singlet ^1^ABR across the library, whereas ^1^TS_conc_ retains closed-shell character. Here, the expectation value ⟨*Ŝ* ^2^⟩_BS_ provides a diagnostic (closed-shell singlet: ⟨*S* ^2^⟩ ≈ 0; open-shell singlet with substantial triplet admixture: ⟨*S* ^2^⟩ ≈ 1): accordingly, ^1^ABR exhibits ⟨*Ŝ* ^2^⟩_BS_ ≈ 1, while ^1^TS_conc_ remains near ⟨*Ŝ* ^2^⟩_BS_ ≈ 0. Because the two barriers differ by less than about 2 kcal mol^–1^—within the expected uncertainty of the method—and Δ*G*_TBR_^‡^ is defined as the larger of the two, the barrier is insensitive to which transition state is rate-determining when the two are nearly degenerate, leaving the gate classification unaffected. The overall barrier trend therefore remains governed by the M–Cp-controlled term in eq 2. Full one-dimensional thermal back-reaction potential-energy surfaces and numerical barriers are provided in Supporting Section S6. Together, these results show that periodic and relativistic strengthening of M–Cp bonding intrinsically couples storage capacity with barrier height, enforcing the storage–stability trade-off quantified below.

#### Integrated Performance Map

The storage and kinetic trends collectively establish an *opposing-gradient rule*: heavier, more covalent metals systematically lower Δ*G*_storage_ while raising Δ*G*_TBR_^‡^. An integrated performance map (Figure 2d) visualizes this trade-off, plotting each dimer by its Δ*G*_TBR_^‡^ (abscissa, *x*; 298 K half-lives on a secondary axis) versus its Δ*G*_storage_ (ordinate, *y*). This map serves as a design “phase diagram”, showing how metal choice navigates an intrinsic storage–stability surface. The resulting landscape resembles a Pareto-like frontier: improvements in stored energy are achieved only at the expense of kinetic persistence, and vice versa. Movement along the diagonal therefore reflects controlled traversal of a single underlying bond-strength manifold rather than independent optimization of storage and stability, so that metal substitution primarily generates nondominated compromises rather than unconstrained improvements in both objectives. In the language of charge-shift bonding,^48^ these opposing gradients arise because semicore–shell Pauli repulsion—and its relativistic relief—jointly tune *D*_LEI_(M–M′) and *D*_HEI_(M–Cp) in eqs 1 and 2.

This design constraint in bimetallic MOST systems thus becomes explicit. Group 6 homobimetallics define the high-storage/low-barrier limit: Cr_2_ delivers the largest Δ*G*_storage_ but an impractically small Δ*G*_TBR_^‡^. Group 8 homobimetallics move toward the opposite regime: Fe_2_ discharges too rapidly, Ru_2_ lies in a balanced window, and Os_2_ is stabilized to an excessively long lifetime. Heterobimetallic dimers fall within a narrow diagonal band between their homoparents, confirming that metal pairing primarily translates candidates along an intrinsic storage–stability surface rather than toward an unconstrained optimum.

A linear least-squares fit across all (*N* = 21) dimers quantifies this trend

$$\Delta G_{\mathrm{storage}}\approx a_{\mathrm{all}}+m_{\mathrm{all}}\Delta G_{\mathrm{TBR}}^{‡},,m_{\mathrm{all}}=-0.40\left(\pm 0.06\right),R^{2}=0.67$$

where *a*_all_ is the regression intercept and *m*_all_ is the slope (uncertainty as one standard error). The associated Pearson correlation coefficient is *r* = −0.82, and the rank-based Spearman coefficient—which tests the monotonic trade-off without assuming strict linearity—is ρ = −0.80 (*p* < 10^–4^); the opposing-gradient rule is thus a statistically robust, moderately strong correlation rather than a scatter of unrelated points. Subset fits for homobimetallics (*N* = 6; *m*_homo_ = −0.43, *R* ^2^ = 0.68) and heterobimetallics (*N* = 15; *m*_hetero_ = −0.38, *R* ^2^ = 0.67) recover the same slope within uncertainty, consistent with a shared trade-off manifold; given the small size of each subset, we read this as consistency rather than as independent confirmation. This behavior is consistent with a linear free-energy relationship^50,51^ in which the bond-exchange scaffold defines a common reaction family and the M–Cp bond strength acts as the principal thermodynamic control parameter governing both storage energetics and thermal-release barriers.

Δ*G*_TBR_^‡^ therefore emerges as a single dominant descriptor, with *R* ^2^ = 0.67 indicating that the linear model accounts for roughly two-thirds of the variance in Δ*G*_storage_; equivalently, the root-mean-square residual (RMSE) about the fit is 2.74 kcal mol^–1^. The remaining variance reflects second-order contributions beyond the bond-exchange model, including asymmetric polarization, entropic effects, and residual methodological error. We stress that this correlation is descriptive, not predictive. Its role is to establish that a single bond-strength lever governs both objectives across the 3*d*/4*d*/5*d* library; it is not a regression from which the storage energy or lifetime of an untested dimer can be read off, since the prediction interval for a new candidate—roughly ±2 RMSE ≈ ±5.5 kcal mol^–1^—spans an appreciable fraction of the ∼19 kcal mol^–1^ storage window. Thus, candidate selection in Gate II rests not on the fitted line but on *window membership* in the (Δ*G*_TBR_^‡^, Δ*G*_storage_) plane, a categorical criterion that is robust to the residual scatter (*vide infra*). Full regression and correlation statistics, including subset confidence intervals, are provided in Supporting Section S7. The slope itself remains physically informative: algebraically inverting the fitted relationship

$$\Delta G_{\mathrm{TBR}}^{‡}\approx \frac{\Delta G_{\mathrm{storage}}-a_{\mathrm{all}}}{m_{\mathrm{all}}}$$

gives 1/*m*_all_ ≈ −2.5, indicating that a 1 kcal mol^–1^ increase in Δ*G*_storage_ is accompanied by a ∼2.5 kcal mol^–1^ decrease in Δ*G*_TBR_^‡^ on average across the library—a magnitude-bearing statement about the trade-off rate, distinct from any claim of per-compound predictability.

Assuming first-order thermal back-reaction kinetics

3 $$-\frac{d\left[\mathrm{HEI}\right]}{dt}=k\left[\mathrm{HEI}\right]$$

the half-life of the HEI, *t*_1/2_ = ln 2/*k*, follows from conventional transition-state theory^52^ via the Eyring equation

4 $$t_{1/2}=\frac{h\ln2}{k_{B}T}e^{\Delta G_{\mathrm{TBR}}^{‡}/\mathrm{RT}}$$

where *h* is Planck’s constant, *k*_B_ is Boltzmann’s constant, *R* is the gas constant, and *T* = 298.15 K throughout. This gives *t*_1/2_ ≈ 1 min at Δ*G*_TBR_^‡^ ≈ 20.1 kcal mol^–1^ and *t*_1/2_ ≈ 100 year at Δ*G*_TBR_^‡^ ≈ 30.6 kcal mol^–1^, with smaller barriers corresponding to subminute discharge. The light-gray band in Figure 2d (20.1–30.6 kcal mol^–1^) thus spans *t*_1/2_ ≈ 1 min–100 year, within which the lifetime is highly sensitive to barrier height: each additional kcal mol^–1^ lengthens *t*_1/2_ by ∼5.4×. Because DFT-computed barriers carry intrinsic uncertainties of a few kcal mol^–1^,^53,54^ which translate into half-life (*t*_1/2_) uncertainties of orders of magnitude, we adopt this broad window as the operative Gate II viability criterion. A narrower “daily-use” window (*t*_1/2_ = 1 day–1 year; 24.4–27.9 kcal mol^–1^) is highlighted by the dark-gray band (Figure 2d) and serves as an illustrative operational target rather than a selection criterion. Notably, only Ru_2_ and OsW fall within this daily use window at 298 K, placing them on the practical storage–stability frontier as the chemically relevant nondominated candidates within the present design space. Ru_2_ lies near the lower edge, combining high storage (19.3 kcal mol^–1^) with manageable lifetimes, whereas OsW sits at the upper edge, delivering ∼16.0 kcal mol^–1^ of storage with Δ*G*_TBR_^‡^ ≈ 25.4 kcal mol^–1^ and correspondingly robust retention. We emphasize that the ∼1 kcal mol^–1^ barrier difference between OsW and Ru_2_ lies well within method uncertainty and carries no interpretive weight; the significance of OsW is that it independently satisfies the gate criteria, not that it outperforms the benchmark. Accordingly, the classification is robust: under uniform shifts of all computed barriers by ±3 kcal mol^–1^, Ru_2_ and OsW remain within the viability window, Os_2_ remains kinetically trapped, and the Group 6 homobimetallics remain too labile, leaving the qualitative classification unchanged; only candidates near the window edges (e.g., Fe_2_, OsMo) may enter or leave under such shifts and are therefore not advanced as predictions. Several dimers cluster just outside the daily use band within this broader window (Figure 2d): OsMo matches Ru_2_ in storage (19.3 kcal mol^–1^) but discharges too quickly (*t*_1/2_ < 1 day); OsFe offers extended retention (*t*_1/2_ > 1 year) but at the cost of lower storage (13.3 kcal mol^–1^); and RuFe occupies the complementary regime (17.4 kcal mol^–1^, *t*_1/2_ < 1 day). Together, these near-frontier candidates delineate the practical boundaries of the storage–stability landscape.

Taken together, Gate II identifies OsW as satisfying the thermodynamic and kinetic requirements for a closed-loop MOST cycle. However, storage competence is only a necessary condition: productive photocharging in fulvalene-bridged dimers requires access to the triplet-mediated isomerization manifold via spin-gated ISC. We therefore turn to Gate I, focusing on Ru_2_ (benchmark) and OsW (frontier lead) while treating RuFe, OsFe, and OsMo as mechanistic comparators near the frontier (Supporting Sections S8.1 and S8.2) to test whether OsW can access the isomerizing triplet state required for productive photocharging.

### Gate I: Photochemical Viability (Excited-State Screen)

Gate I imposes two requirements: the ^1^LEI must absorb near-visible light, and the resulting bright singlet population (S*) must efficiently reach the isomerizing triplet state (*T*_iso_) via ISC (Figure 1b). These requirements reflect the central role of excited-state ordering in metal-containing systems, where productive photochemistry depends on the relative alignment of singlet and triplet manifolds.^23,55,56^

To evaluate these criteria, we computed Franck–Condon (FC) absorption spectra for Ru_2_ and OsW using time-dependent density functional theory within the Tamm–Dancoff approximation (TDDFT/TDA)^57^ at the PBE0-D4 ^58^ level (Figure 3a,b); methodological details are provided in Supporting Section S2. The protocol is validated both by functional-sensitivity tests (Supporting Section S9) and by direct benchmarking against extended multistate complete active space second-order perturbation theory (XMS-CASPT2), established for Ru_2_ previously^23^ and extended to OsW here (Supporting Section S12). Across a substantial region around the FC point the relevant states retain predominantly single-reference character, making TDDFT/TDA a reliable and efficient approach for evaluating Gate I state ordering.

**Figure 3:**
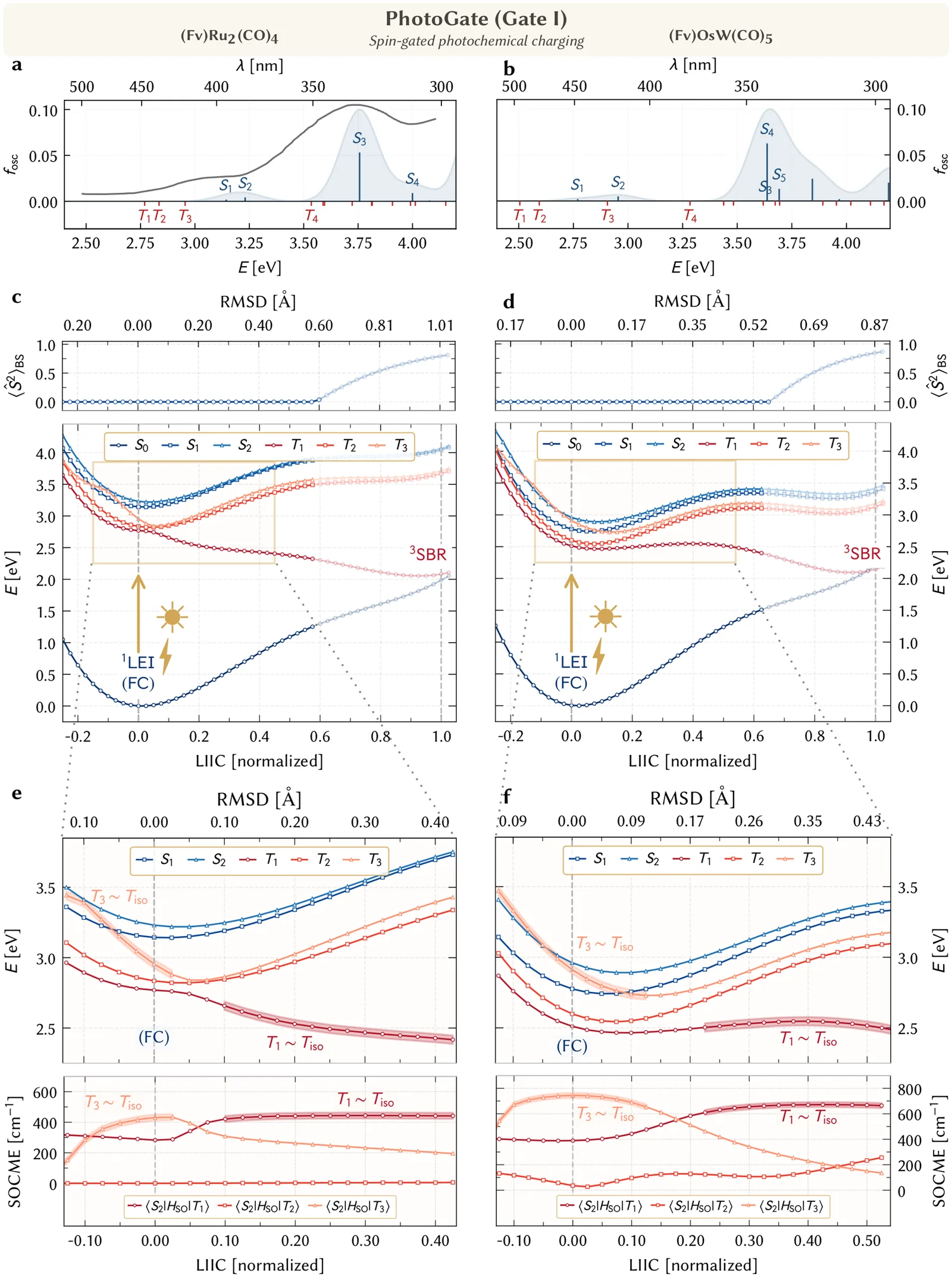
Gate I: photochemical viability of fulvalene-bridged bimetallic MOST dimers. (a, b) Franck–Condon absorption spectra for (Fv)­Ru_2_(CO)_4_ and (Fv)­OsW­(CO)_5_ computed using TDDFT/TDA at the PBE0-D4/def2-TZVPP/ZORA level, highlighting the lowest bright singlet states (*S*_1_, *S*_2_) that define the onset of solar harvesting. For (Fv)­Ru_2_(CO)_4_, the experimental spectrum^59^ is overlaid in gray (λ_onset_ ≈ 450 nm, λ_max_ ≈ 330 nm). (c, d) Adiabatic potential-energy profiles along the M–M′ bond-cleavage coordinate connecting the Franck–Condon (FC) region to the triplet biradical ^3^SBR; top: ⟨*Ŝ* ^2^⟩_BS_ of *S*_0_, validating the single-reference description near FC (pale boxes); faded curves mark its breakdown toward ^3^SBR. (e, f) Expanded view of the FC region showing the evolution of the isomerizing triplet *T*_iso_ and its crossings with *S*_1_/*S*_2_, with spin–orbit coupling matrix elements (SOCMEs, cm^–1^) for *T*_1_–*T*_3_ below.

We then map the energetic alignment between the bright singlet manifold (S*) and the isomerizing triplet (*T*_iso_) along the M–M′ bond-cleavage coordinate (Figure 3c–f). This analysis resolves the mechanistic pathway linking optical excitation to triplet biradical formation and quantifies the spin–orbit coupling that mediates intersystem crossing in the FC region.

#### Franck-Condon Absorption

For homobimetallic Ru_2_ (Figure 3a), the bright-onset manifold S* begins with *S*_1_ and *S*_2_ at 394 and 383 nm (3.14 and 3.23 eV), though both remain weakly allowed (*f* ≈ 2 × 10^–3^ and 4 × 10^–3^). The first intense transition occurs at *S*_3_ (329 nm/3.76 eV; *f* ≈ 5 × 10^–2^), while the lowest triplets (*T*_1_–*T*_3_) lie at 447–419 nm (2.77–2.96 eV). Consistent with experiment, (Fv)­Ru_2_(CO)_4_ exhibits an absorption onset at λ_onset_ ≈ 450 nm and a maximum at λ_max_ ≈ 330 nm.^59^ These energies agree well with XMS-CASPT2 results.^23^ Natural transition orbitals (NTOs) show that the low-lying states are metal-centered and, by symmetry, essentially equally delocalized over both Ru sites (Supporting Sections S10 and S11). Within a σ/π framework,^23^ *S*_1_ and *S*_2_ correspond to π_⊥_^*^ → σ* and π_∥_^*^ → σ* promotions. *T*_1_ and *T*_2_ mirror these parent excitations, whereas *T*_3_ is primarily σ → σ*. Here and throughout, σ, π, and δ denote orbitals of the metal–metal manifold, with asterisks marking antibonding counterparts and the subscripts ⊥ and ∥ distinguishing the out-of-plane and in-plane π components.

The heterobimetallic OsW complex (Figure 3b) retains this metal-centered architecture but shifts the entire manifold bathochromically, bringing the lowest singlet transitions into the near-visible. Here S* begins with *S*_1_ and *S*_2_ (2.77 and 2.96 eV; 447 and 419 nm; *f* ≈ 2 × 10^–3^ and 5 × 10^–3^), with *T*_1_ and *T*_2_ immediately below (2.51–2.59 eV; 494–478 nm). A higher-energy bright cluster follows at 3.64–3.84 eV (340–322 nm; *f* ≈ 10^–2^–6 × 10^–2^). This redistribution of oscillator strength enhances absorption in the 420–450 nm region, strengthening near-visible addressability and the optical entry point for spin-gated photocharging. NTO analysis shows that the excitations remain predominantly metal-centered but become strongly polarized toward W, consistent with symmetry breaking in the heterodimer ( Supporting Sections S10 and S11). Accordingly, *S*_1_ retains largely π_⊥_^*^ → σ* character, whereas *S*_2_ is dominated by a δ → σ* promotion; both are essentially W-centered. *T*_1_ and *T*_2_ again track their singlet counterparts, while *T*_3_ remains chiefly σ → σ* and is more evenly distributed across the OsW core. For (Fv)­OsW­(CO)_5_, this TDDFT/TDA description was benchmarked against XMS-CASPT2 (Supporting Section S12): vertical excitation energies agree to a mean absolute deviation of 0.11 eV, and both methods assign the same σ → σ* state as *T*_iso_, at near-degeneracy with the bright singlet manifold in the FC region.

#### Adiabatic Potential Energy Profiles

We map adiabatic potential-energy surfaces along a normalized linear internal coordinate (LIIC) connecting the FC geometry of ^1^LEI (LIIC = 0) to the bond-cleaved triplet *syn*-biradical ^3^SBR (LIIC = 1; Figure 3c,d). The LIIC is extended to negative values to capture local curvature around the equilibrium, FC geometry, and the upper abscissa reports the RMSD from there (Å).

At the FC point, the metal–metal distances are 2.84 Å (Ru_2_) and 3.08 Å (OsW), with nearly planar fulvalene bridges. At ^3^SBR, these increase to 4.11 and 4.15 Å, respectively, and the corresponding ϕ (M–C–C–M′) dihedral angles open to 33.8° and 25.1°. The associated RMSDs (1.01 and 0.87 Å for Ru_2_ and OsW) support the use of a single normalized LIIC for comparison. Geometries for the remaining dimers (including OsMo and the Fe-containing outliers) are provided in Supporting Sections S8.1 and S8.2; Fe-containing dimers deviate from the canonical bond-cleavage topology and are not pursued further here.

We assess the reliability of TDDFT along the LIIC using the singlet broken-symmetry diagnostic ⟨*Ŝ* ^2^⟩_BS_ of *S*_0_ (Figure 3c,d; top), which provides a practical measure of multireference character—manifested as spin contamination—within a single-determinant DFT framework.^60^ For both complexes, ⟨*Ŝ* ^2^⟩_BS_ ≈ 0 over ∼70% of the path, including the FC-adjacent region highlighted by the pale boxes, supporting a dominant single-reference description in this window. We therefore interpret crossings and state reorderings primarily within this validated region and de-emphasize the terminal segment approaching ^3^SBR, where ⟨*Ŝ* ^2^⟩_BS_ increases and the single-reference description deteriorates (reduced opacity in Figure 3c,d). This single-reference-to-multiconfigurational transition along the bond-cleavage coordinate, consistent with prior observations in cyclobutadiene–metal complexes^61^ and in the diruthenium MOST benchmark,^23^ supports our cautious interpretation of the TDDFT results within the FC-adjacent window.

Along the LIIC, we plot the three lowest singlets (*S*_0_–*S*_2_) and triplets (*T*_1_–*T*_3_). The excited-state topology is conserved between the two complexes (Figure 3c,d): *S*_1_ and *S*_2_ form shallow minima near FC, whereas only one triplet stabilizes along the path to ^3^SBR, with the remaining triplets staying FC-centered. We therefore focus on the FC-adjacent window to identify the diabatic states, isolate the isomerizing triplet manifold, and locate crossings with the bright *S*_1_/*S*_2_ states.

#### Diabatic Triplet Reservoir and ISC Seams

Because TDDFT yields adiabatic eigenstates whose ordering and character interchange along the LIIC, a purely adiabatic description obscures the diabatically continuous isomerizing triplet *T*_iso_ that correlates with ^3^SBR. To recover this reactive channel, we construct a quasi-diabatic representation (Figure 3e,f; top) by tracking the dominant metal–metal π/σ excitation character and using spin–orbit coupling matrix elements (SOCMEs) with *S*_1_ and *S*_2_ as state-specific fingerprints to follow triplet identities. For clarity, Figure 3e,f (bottom) plots ⟨*S*_2_|*Ĥ*_SO_|*T*_*j*_⟩ (*j* = 1–3), which produces a cleaner avoided-crossing pattern than ⟨*S*_1_|*Ĥ*_SO_|*T*_*j*_⟩. This SOCME-guided tracking identifies the diabatically continuous *T*_iso_ within the adiabatic triplet manifold; because the diabatic SOCMEs vary smoothly and only weakly with geometry, their characteristic magnitudes serve as stable fingerprints for following a given diabatic state through the adiabatic avoided crossings. Within this quasi-diabatic framework, SOCMEs between diabatic states are treated as geometry-independent constants (Condon approximation), such that ISC rates are governed primarily by the energetic proximity of the crossing seam. This is supported by the smooth, slowly varying SOCMEs computed explicitly at each LIIC point within the FC-proximal window (Figure 3e,f, bottom).

For both Ru_2_ and OsW, *T*_iso_ aligns with the adiabatic *T*_3_ state near the FC geometry (including negative LIIC), as indicated by the dominant SOCME ⟨*S*_2_|*Ĥ*_SO_|*T*_3_⟩. Moving toward bond cleavage (positive LIIC), *T*_iso_ evolves into *T*_1_ via a *T*_1_/*T*_3_ avoided crossing, marked by exchange of their SOCME fingerprints. Quantitatively, the SOCMEs linking the lowest bright singlet (*S*_1_) to the reactive triplets are already large at FC: for Ru_2_, ⟨*S*_1_|*Ĥ*_SO_|*T*_2_⟩ and ⟨*S*_1_|*Ĥ*_SO_|*T*_3_⟩ are ∼270 and 330 cm^–1^ at RMSD = 0, whereas for OsW, ⟨*S*_1_|*Ĥ*_SO_|*T*_2_⟩ and ⟨*S*_1_|*Ĥ*_SO_|*T*_3_⟩ reach ∼340 and 760 cm^–1^ at FC. NTO analysis at representative points left of, at, and right of the FC geometry confirms the corresponding evolution of triplet-state character (Supporting Section S10).

Along this coordinate (pre*-*FC = left, post*-*FC = right), Ru_2_ first forms a near-degenerate seam between *S*_2_ and *T*_iso_ slightly before FC and then crosses *S*_1_ at a comparable displacement (both <0.10 Å RMSD). Thus, the FC point is bracketed by sequential *S*_2_/*T*_iso_ and *S*_1_/*T*_iso_ crossings, consistent with the highlighted *T*_3_ ∼ *T*_iso_ and *T*_1_ ∼ *T*_iso_ segments in Figure 3e.

For OsW, *T*_iso_ likewise first crosses *S*_2_ slightly on the pre-FC side (<0.05 Å RMSD), whereas the *S*_1_/*T*_iso_ crossing occurs on the post-FC side (<0.10 Å RMSD), as reflected by the corresponding highlighted segments in Figure 3f. Because these crossings occur extremely close to the FC geometry, only minimal geometric distortion is required to activate ISC.

Accordingly, Ru_2_ and OsW share the same near-FC crossing topology: sequential *S*_2_/*T*_iso_ and *S*_1_/*T*_iso_ seams that bracket FC and are driven by metal–metal π/σ excitations. However, OsW provides a stronger spin-gated funnel into *T*_iso_ owing to its larger SOCMEs. Replacing Ru–Ru with Os–W therefore strengthens spin gating while preserving the Franck–Condon-adjacent S*/*T*_iso_ alignment required to avoid Marcus-inverted suppression of the decisive ISC step.

Within Gate I, FC-proximal near-degeneracy between a bright singlet and *T*_iso_ under strong SOC emerges as the operational criterion for photochemical viability. The known spin-gated behavior of Ru_2_ is recovered, providing a reliable reference for interpreting OsW, which preserves efficient photocharging while enhancing ISC into *T*_iso_.

### Triplet Biradical Torsional Relaxation

Passing Gate I delivers the excited population into the triplet manifold; whether photocharging is then completed depends on the fate of the resulting biradical—specifically, whether it survives long enough to isomerize rather than crossing back to the ground-state. Formation of the *syn*-triplet biradical (^3^SBR) initiates a kinetic competition between reverse intersystem crossing (rISC) to the ground-state and torsional relaxation toward the *anti*-triplet configuration (^3^ABR), the latter driving productive photoisomerization. For (Fv)­Ru_2_(CO)_4_, the experimentally observed ∼2 ns triplet lifetime^21^ provides sufficient time for this *syn*–to–*anti* rotation. Given the nearly identical torsional energy profiles and spin–orbit coupling trends established above, a comparable triplet lifetime is expected for (Fv)­OsW­(CO)_5_.

Previous XMS-CASPT2 calculations established the torsional relaxation pathway for (Fv)­Ru_2_(CO)_4_,^23^ which we revisit here using broken-symmetry DFT and extend to the heterobimetallic analogue (Fv)­OsW­(CO)_5_ (Figure 4). In both systems the triplet biradical minimum occurs at the *syn* configuration (ϕ ≈ 40°), and a small barrier on the triplet surface (ca. 1–2 kcal mol^–1^) via the torsional transition state ^3^TS_rot_ enables facile *syn*–to–*anti* interconversion between ^3^SBR and ^3^ABR.

**Figure 4:**
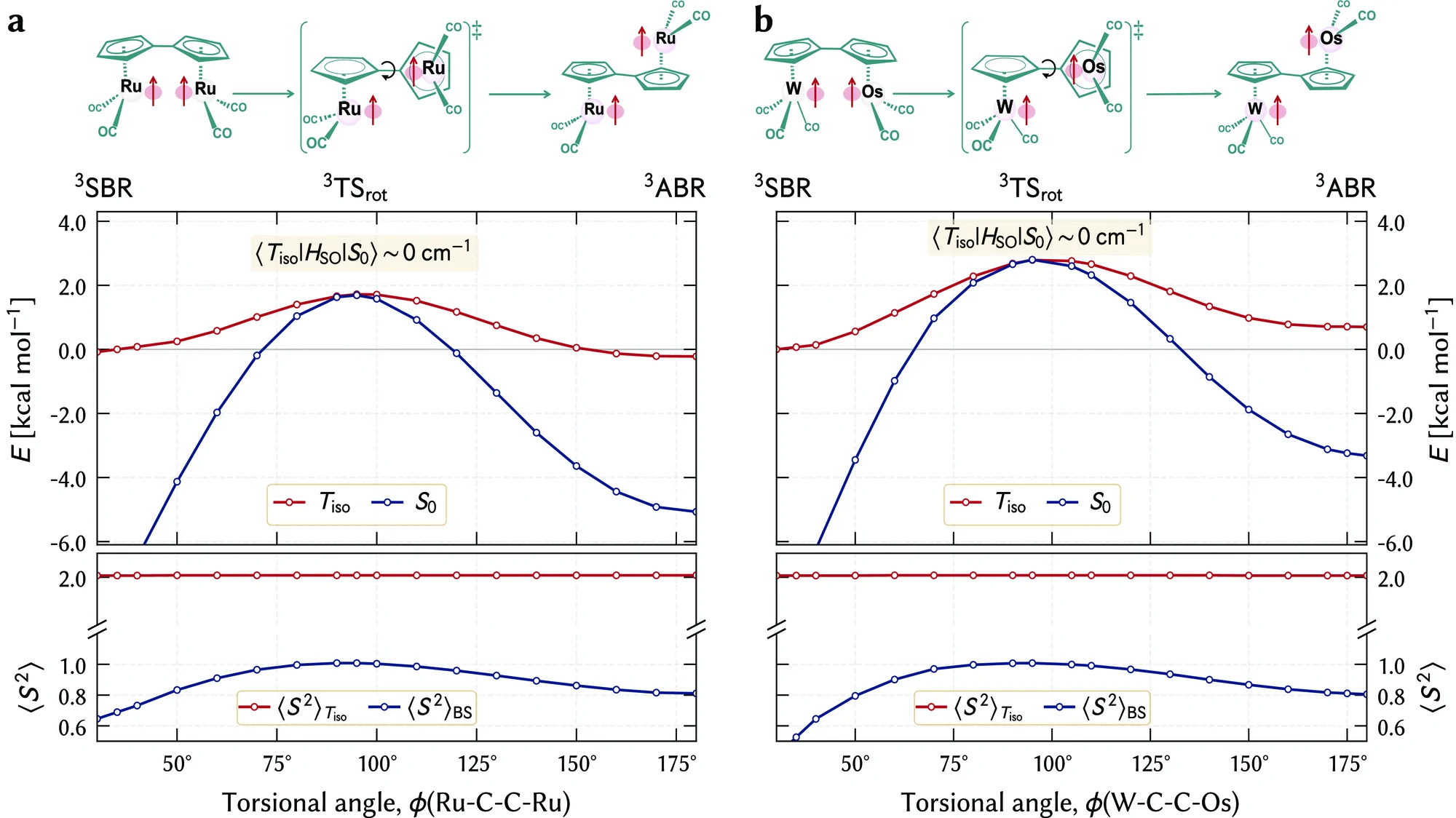
Triplet biradical stability along the fulvalene torsional coordinate. (a) (Fv)­Ru_2_(CO)_4_ and (b) (Fv)­OsW­(CO)_5_. For both systems: Top: Mechanism of the conversion of the *syn*-triplet biradical (^3^SBR) to the *anti*-biradical (^3^ABR) via the torsional transition state (^3^TS_rot_). Middle: Adiabatic potential-energy profiles of the isomerizing triplet (*T*_iso_) and the ground-state singlet (*S*_0_), referenced to ^3^SBR. Near the crossing region (ϕ ≈ 95°), the singlet acquires pronounced open-shell character (⟨*S* ^2^⟩_BS_ ∼ 1), rendering its orbital configuration similar to that of *T*_iso_ and quenching the spin–orbit coupling (⟨*T*_iso_|*Ĥ*_SO_|*S*_0_⟩ ≈ 0 cm^–1^), in accordance with El-Sayed’s rule.^62^ All structures and energies were computed at the B3LYP-D3­(BJ)/def2-TZVPP level with Stuttgart–Dresden effective core potentials for the transition metals. Bottom: Broken-symmetry singlet ⟨*S* ^2^⟩ along the fulvalene dihedral ϕ­(M-C-C-M').

Despite the proximity of the singlet and triplet surfaces near ϕ ≈ 95°, rISC is strongly suppressed. The singlet surface in this region was described using an open-shell broken-symmetry treatment ( Supporting Section S2), with ⟨*S* ^2^⟩_BS_ ∼ 1 confirming its biradical character (Figure 4). As the torsional coordinate evolves, the singlet state adopts an electronic configuration similar to that of the triplet, causing the spin–orbit coupling between *T*_iso_ and *S*_0_ to approach zero in accordance with El-Sayed’s rules.^23,62^

Consequently, torsional relaxation proceeds predominantly on the triplet surface, extending the biradical lifetime and preventing premature metal–metal bond reformation. Importantly, (Fv)­Ru_2_(CO)_4_ and (Fv)­OsW­(CO)_5_ exhibit nearly identical torsional energy profiles and spin–orbit coupling trends along ϕ, indicating mechanistically equivalent triplet relaxation pathways and therefore comparable photoisomerization efficiencies.

## Conclusions

This work demonstrates that MOST viability in fulvalene-bridged bimetallic systems is governed not by independent optimization of storage capacity and photocharging efficiency, but by the simultaneous satisfaction of two coupled mechanistic constraints operating in the ground and excited states, respectively. Thus, ground-state (Gate II) and excited-state (Gate I) filters enable the systematic screening of bimetallic MOST candidates. Gate II reveals that bond-exchange thermochemistry intrinsically couples stored energy and the thermal back-reaction barrier, giving rise to a Pareto-type thermodynamic–kinetic frontier that constrains viable cycling. Gate I requires access to the isomerizing triplet manifold via Franck–Condon-proximal singlet–triplet crossing, while avoiding the Marcus-inverted regime^26,27,30–33^ that suppresses productive intersystem crossing. Applying these sequential filters to 21 fulvalene-bridged dimers—based on the fulvalene carbonyl scaffold established by Vollhardt and co-workers, many members of which have been synthesized^19,21,22,24,25,63–69^ —identifies (Fv)­OsW­(CO)_5_ as a previously unrecognized MOST-competent composition located on the practical storage–stability frontier (Figure 5).

**Figure 5:**
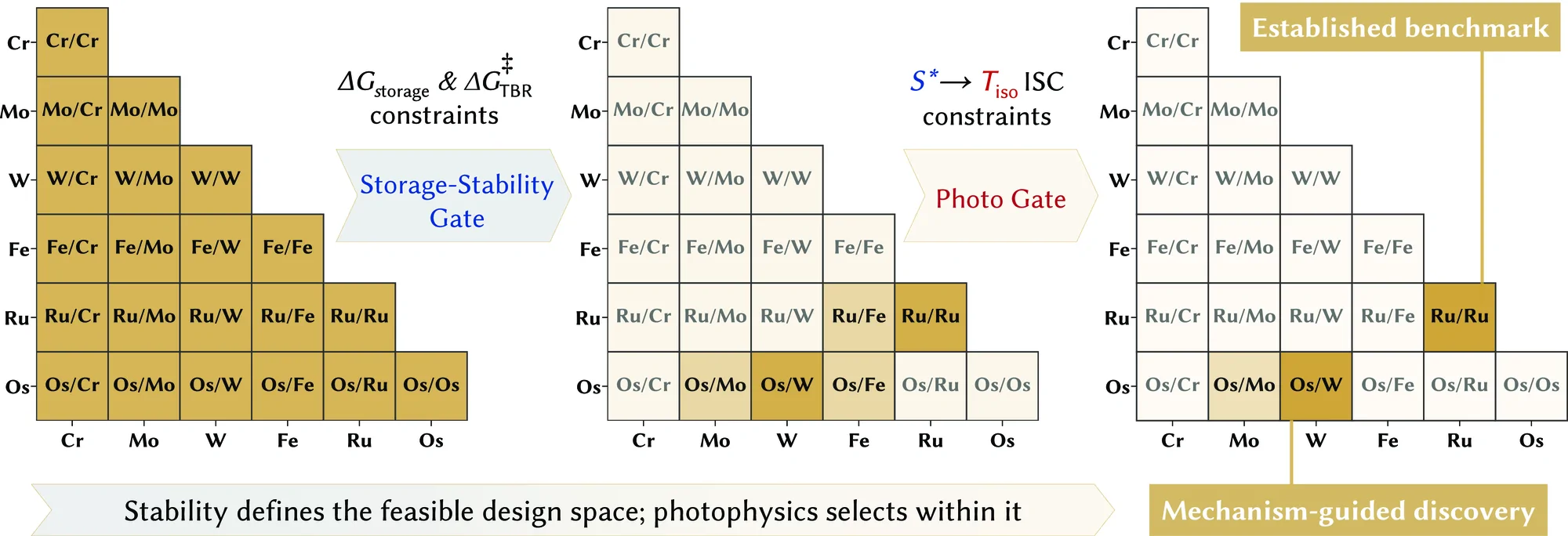
Mechanistically informed, stability-first (Gate II → Gate I) screening for the discovery of fulvalene-bridged bimetallic MOST systems. From left to right, the panels depict the sequential filtering of the complete 21-member design space, with all candidates treated *a priori* as equally plausible. The *Storage*–*Stability Gate* applies the combined Δ*G*_storage_ and Δ*G*_TBR_^‡^ criteria to define the feasible design space, eliminating thermodynamically or kinetically unsuitable systems while leaving three near-misses—(Fv)­OsMo­(CO)_5_, (Fv)­OsFe­(CO)_4_, and (Fv)­RuFe­(CO)_4_—just outside the target window (center). The subsequent *Photo Gate* enforces the S* → *T*_iso_ ISC requirement, excluding (Fv)­OsFe­(CO)_4_ and (Fv)­RuFe­(CO)_4_ and leaving (Fv)­OsMo­(CO)_5_ as the sole genuine near-miss (right). Only two of the 21 candidates—the established benchmark (Fv)­Ru_2_(CO)_4_ and the newly identified (Fv)­OsW­(CO)_5_—satisfy both gates, demonstrating that stability defines the feasible design space, whereas photophysics selects the viable molecular solar–thermal fuels within it.

The Os–W combination achieves favorable storage thermodynamics and thermal persistence while retaining Franck–Condon-proximal access to the isomerizing triplet manifold required for spin-gated photocharging. It preserves near-visible absorption (λ_onset_ ≳ 400 nm), characteristic of Os-containing dimers, while avoiding the extreme kinetic stabilization observed for Os_2_. We note that the gravimetric energy density, elemental abundance, cost, and materials sustainability remain more favorable for the Ru-based benchmark (Supporting Sections S5 and S13). Nevertheless, (Fv)­OsW­(CO)_5_ is the only composition in the 21-member library besides the benchmark to satisfy both mechanistic gates: it matches the storage–stability placement of (Fv)­Ru_2_(CO)_4_ while exhibiting even stronger spin–orbit coupling into the reactive triplet channel. Moreover, the photoisomerization quantum yields reported for (Fv)­Ru_2_(CO)_4_ and its derivatives are modest (≲1%) and viscosity-dependent,^59^ reflecting in part the competition between productive triplet formation and unproductive decay; the substantially larger spin–orbit couplings computed for (Fv)­OsW­(CO)_5_ may therefore translate into improved photocharging quantum yields, an experimentally testable prediction of the present framework. Accordingly, while (Fv)­OsW­(CO)_5_ is not necessarily a replacement for (Fv)­Ru_2_(CO)_4_, it stands as proof that the dual-gate framework can identify compositions—potentially including ones with improved photocharging efficiency—that would remain invisible to empirical metal substitution alone.

The framework further rationalizes why closely related systems fall just inside or outside the viable window: (Fv)­OsMo­(CO)_5_ passes Gate I and lies just below the Gate II daily use window owing to a marginally insufficient thermal barrier (Supporting Section S8.1), whereas the Fe-containing dimers (Fv)­RuFe­(CO)_4_ and (Fv)­OsFe­(CO)_4_ fail Gate I through Marcus-inverted suppression of the decisive S* → *T*_iso_ crossing despite substantial spin–orbit coupling (Supporting Section S8.2). These contrasting outcomes show that the framework explains nonviable as well as viable compositions through common electronic-structure principles, delineating large regions of bimetallic design space as fundamentally inaccessible.

While (Fv)­OsW­(CO)_5_ itself has not yet been reported, computed homodimer-scrambling energetics show that it is thermodynamically stable toward disproportionation into (Fv)­Os_2_(CO)_4_ and (Fv)­W_2_(CO)_6_ (Supporting Section S13); combined with extensive precedent for stable Os–W carbonyl motifs^70–73^ and the selective synthesis of related fulvalene heterobimetallic complexes from mixed-metal precursors,^63,64^ these results support its experimental accessibility and motivate its targeted synthesis.

Although the quantitative ranking of closely related candidates may depend on the level of electronic-structure theory employed, the dual-gate framework and the mechanistic trends identified across the series are robust, as corroborated by the agreement between TDDFT/TDA and XMS-CASPT2 for the key excited-state alignments (Supporting Section S12). These findings establish that MOST performance in organometallic systems emerges from satisfying two coupled mechanistic constraints—one thermodynamic and one spin-dynamical—that cannot be optimized independently. By encoding these constraints as transferable design rules, the dual-gate framework maps the accessible region of bimetallic MOST chemical space and provides a concrete path toward extending the approach beyond carbonyl scaffolds to broader classes of organometallic solar thermal fuels.

## Supplementary Material



## Data Availability

Additional data are available from the corresponding authors upon reasonable request.

## Notes

Full methodological details and extended data underpinning the two-gate screening workflow, including: (i) electron-counting construction of the fulvalene-bridged Group 6/Group 8 bimetallic library (Supporting Section S1); (ii) computational protocols for ground- and excited-state calculations used in Gate II and Gate I, respectively (Supporting Section S2); (iii) DFT functional calibration against experimental storage and thermal back-reaction energetics of (Fv)­Ru_2_(CO)_4_ (Supporting Section S3); (iv) assessment of multireference character via fractional occupation number weighted density (FOD) analysis across the full bimetallic library (Supporting Section S4); (v) gravimetric energy density for the full bimetallic library (Supporting Section S5); (vi) complete ^1^HEI → ^1^LEI thermal back-reaction free-energy surfaces for all homo- and heterobimetallic candidates (Supporting Section S6); (vii) regression and correlation statistics for the storage–stability trade-off across the complete bimetallic library (Supporting Section S7); (viii) TDDFT evaluation of key comparator systems, including LIIC/SOC-based assessment of ISC accessibility: the Gate II near-miss (Fv)­OsMo­(CO)_5_ (Supporting Section S8.1) and the Gate I-failing Fe-containing dimers (Fv)­RuFe­(CO)_4_ and (Fv)­OsFe­(CO)_4_ (Supporting Section S8.2); (ix) Gate I excited-state benchmarks for (Fv)­Ru_2_(CO)_4_ and (Fv)­OsW­(CO)_5_: TDDFT functional-sensitivity tests (Supporting Section S9), NTO characterization (Supporting Section S10), and fragment-resolved (localized vs charge-transfer) decomposition (Supporting Section S11); (x) benchmarking of the TDDFT/TDA protocol against XMS-CASPT2 for the low-lying excitation manifold of (Fv)­OsW­(CO)_5_ (Supporting Section S12); and (xi) thermodynamic stability of (Fv)­OsW­(CO)_5_ against homodimer scrambling (Supporting Section S13) (PDF)

## Notes

This work is dedicated to the memory of Prof. Rudolph A. Marcus (1923–2026), whose Nobel Prize–winning electron transfer theory provides the conceptual foundation for the boundary/inverted-regime behavior underlying the intersystem crossing constraint—the “Photo Gate”—central to this study.
