# Supplementary material for: Predictive Design of Fulvalene-Bridged Bimetallic Molecular Solar–Thermal Fuels
Source: JACS Au. 2026 Aug 16;6(10):5648–62. doi: 10.1021/jacsau.6c00919 (PMC13714945; doi:10.1021/jacsau.6c00919)
Supplement: Supplementary file 1 [file au6c00919_si_001.pdf]

Supporting Information for:

Predictive Design of Fulvalene-Bridged Bimetallic Molecular Solar–Thermal Fuels

Gaurab Ganguly* and Leticia González*

Institute of Theoretical Chemistry, Faculty of Chemistry, University of Vienna, Währinger Str. 17, 1090 Vienna, Austria

E-mail: gaurab.ganguly@univie.ac.at; leticia.gonzalez@univie.ac.at

This work is dedicated to the memory of Prof. Rudolph A. Marcus (1923–2026), whose Nobel Prize-winning electron transfer theory provides the conceptual foundation for the boundary/inverted-regime behavior underlying the intersystem crossing constraint—the "Photo Gate"—central to this study.

Table of Contents of the Supporting Information:

S1 Electron-Counting Design of the Fulvalene-Bridged Group 6/Group 8 Molecular Solar–Thermal Library S-3

S2 Computational Details for Ground- and Excited-State Calculations S-4

S2.1 Ground-State Calculations S-4

S2.2 Excited-State Calculations S-5

S3 Density Functional Benchmark for $(Fv)Ru_2(CO)_4$: GGA and Hybrid Rungs of Jacob's Ladder S-6

S4 Assessment of Multireference Character: Fractional Occupation Number Weighted Density (FOD) Analysis S-7

S5 Practical Metrics: Gravimetric Energy Storage Density S-10

S6 Gate II: Thermal Back-Reaction Potential-Energy Surfaces of Fulvalene-Bridged Bimetallic Complexes S-12

S7 Regression and Correlation Statistics for the Storage–Stability Tradeoff S-19

S8 Gate I: TDDFT Analysis of Comparator Bimetallic Systems S-20

S8.1 $(Fv)OsMo(CO)_5$: Gate I Pass, Gate II Near-Miss S-20

S8.2 Fe-Containing Dimers: Intrinsic Gate I Failure S-22

S9 Gate I: TDDFT Benchmark of $(Fv)Ru_2(CO)_4$ and $(Fv)OsW(CO)_5$ — Franck–Condon Absorption, LIIC Topology, and Spin–Orbit Coupling S-25

S10 Gate I: Natural Transition Orbital Analysis S-29

S10.1NTOs Along the LIIC for $(Fv)Ru_2(CO)_4$ S-30

S10.2NTOs Along the LIIC for $(Fv)OsW(CO)_5$ S-33

S11 Gate I: Fragment-Resolved Excited-State Character in $(Fv)Ru_2(CO)_4$ and $(Fv)OsW(CO)_5$ S-36

S12 Gate I: Benchmarking TDDFT/TDA Against XMS-CASPT2 for the Low-Lying Excitation Manifold of $(Fv)OsW(CO)_5$ S-38

S13 Stability of $(Fv)OsW(CO)_5$ toward Disproportionation S-40

S1 Electron-Counting Design of the Fulvalene-Bridged Group 6/Group 8 Molecular Solar–Thermal Library

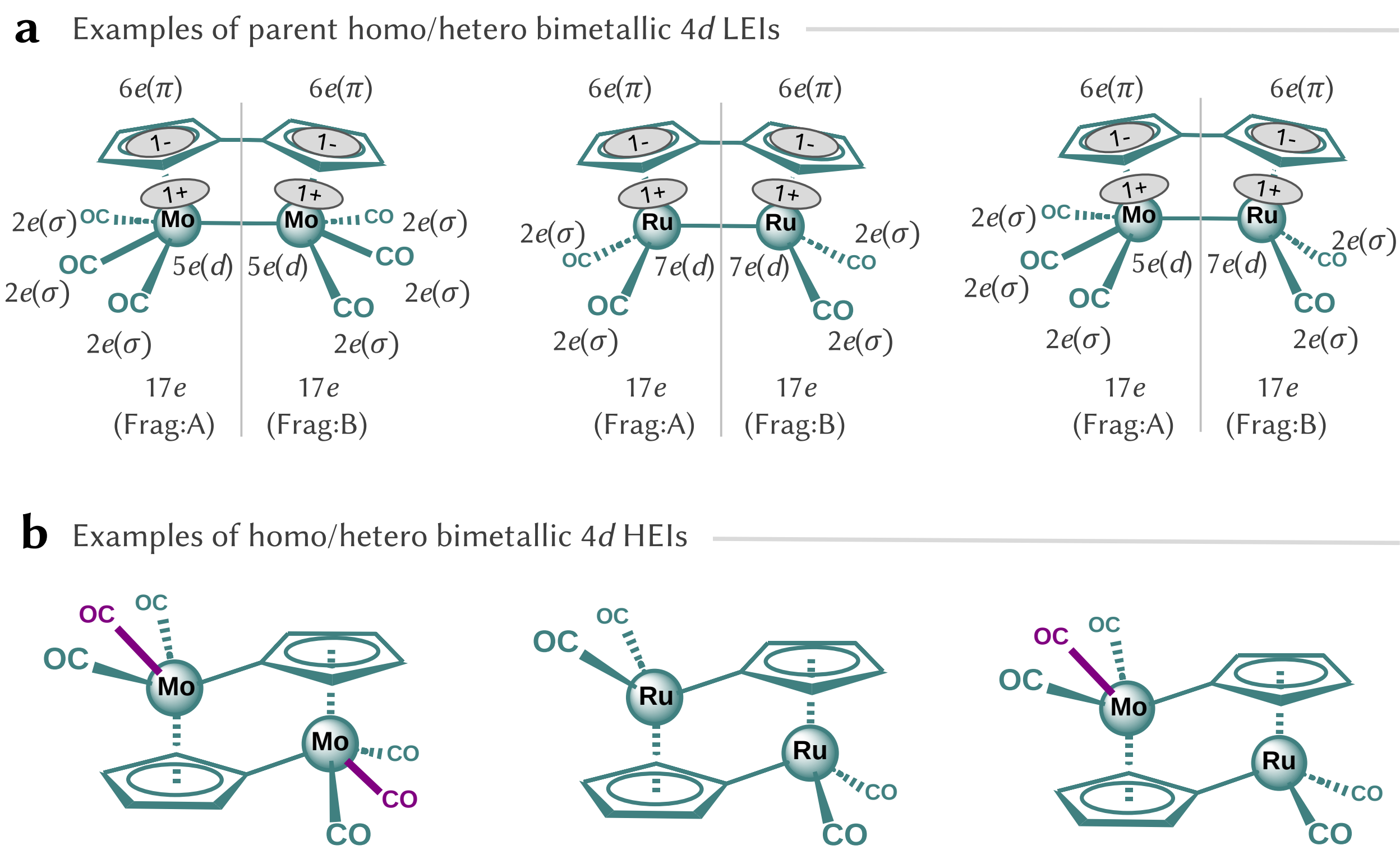

Figure S1. Electron-counting design of the fulvalene-bridged Group 6/Group 8 MOST library. (a) Seventeen-electron fragmentation of parent homo- and heterobimetallic 4d LEIs, illustrated for (Fv)$Mo_2(CO)_6$, (Fv)$Ru_2(CO)_4$, and (Fv)MoRu$(CO)_5$, into (Cp)M$(CO)_x$ fragments ($x = 2, 3$) fixes each Cp^- as a 6e(π) donor, each CO as a 2e(σ) donor, and enforces a single 2e M—M scissile bond with 17e at each metal center. (b) Representative homo- and heterobimetallic 4d HEIs derived from the same electron-precise scheme, in which one carbonyl ligand migrates upon M—M bond cleavage; analogous constructions extend across the full Group 6/Group 8 set.

S2 Computational Details for Ground- and Excited-State Calculations

S2.1 Ground-State Calculations

All ground-state electronic-structure calculations were performed using the GAUSSIAN 16 program package.[S1] Geometry optimizations and harmonic frequency calculations were carried out within Kohn–Sham density functional theory using the hybrid B3LYP exchange–correlation functional[S2,S3] augmented by the D3(BJ) dispersion correction.[S4] This level of theory was adopted for all ground-state structures and energetics discussed in the manuscript and was selected on the basis of benchmarking against experimental storage and thermal back-reaction energetics for (Fv)$Ru_2(CO)_4$ (Section S3).

Scalar relativistic effects for the transition metals were treated using second-order Stuttgart–Dresden effective core potentials[S5] together with their associated valence basis sets. For lighter atoms (C, H, and O), the def2-TZVPP basis set[S6] was employed. All calculations used tight self-consistent-field convergence criteria and ultrafine numerical integration grids throughout. Unless otherwise noted, all calculations were performed in the gas phase without inclusion of an implicit solvent model.

For open-shell species, the nature of the converged SCF wave functions was verified using two complementary diagnostics. First, the expectation value of the spin-squared operator, $\langle S^2 \rangle$, was examined: typical values are approximately 0.0 for closed-shell singlets (CSS), approximately 1.0 for spin-contaminated open-shell singlets (OSS), and approximately 2.0 for open-shell triplets (OST). Second, the corresponding electronic structures were analyzed using natural spin densities and the associated Lewis-type bonding patterns.

For open-shell singlet solutions, spin-projection corrections were applied to the spin-contaminated broken-symmetry singlet energy (${}^1E_{\mathrm{UDFT}}$) to obtain the spin-corrected energy (${}^1E_{\mathrm{SC}}$) according to the approximate spin-projection scheme of Yamaguchi and co-workers:[S7,S8]

$$ {}^1E_{\mathrm{SC}} = {}^1E_{\mathrm{UDFT}} + f_{\mathrm{SC}} \left({}^1E_{\mathrm{UDFT}} - {}^3E_{\mathrm{DFT}} \right), \tag{1} $$

where

$$ f_{\mathrm{SC}} \approx \frac{{}^1\langle S^2 \rangle}{{}^3\langle S^2 \rangle - {}^1\langle S^2 \rangle}. \tag{2} $$

All stationary points were characterized by harmonic vibrational frequency analysis. Local minima were confirmed by the absence of imaginary frequencies, whereas transition states exhibited a single imaginary frequency corresponding to motion along the relevant reaction coordinate. Gibbs free energies at 298 K were obtained from harmonic vibrational analyses including zero-point, thermal, and entropic corrections.

The storage free energy $\Delta G_{\mathrm{storage}}$ was defined as the free-energy difference between the

higher-energy isomer (HEI) and the lower-energy isomer (LEI). The thermal back-reaction barrier $\Delta G^{\ddagger}_{\mathrm{TBR}}$ was defined as the larger of the two activation free energies along the HEI → LEI pathway,

$$\Delta G_{\mathrm{TBR}} = \max\left\{\Delta G^{\ddagger}(^{1}TS_{\mathrm{conc}}),\ \Delta G^{\ddagger}(^{1}TS_{\mathrm{rot}})\right\}. \quad (3)$$

To confirm the barrier topology and identify the rate-determining step, one-dimensional potential-energy scans were additionally constructed along the metal–metal bond elongation coordinate.

S2.2 Excited-State Calculations

Excited-state calculations and spin–orbit couplings between singlet and triplet states were performed using the ORCA 6.1 program package.[S9,S10] Vertical excitation energies, oscillator strengths, and natural transition orbitals (NTOs) were computed using time-dependent density functional theory within the Tamm–Dancoff approximation (TDDFT/TDA) at the PBE0-D4 level,[S11] using the def2-TZVPP basis set with scalar-relativistic corrections treated within the ZORA framework.[S12] Spin–orbit coupling matrix elements (SOCMEs) were evaluated using the spin–orbit mean-field (SOMF) approximation as implemented in ORCA. NTOs were analyzed with the THEODORE package,[S13] which enables decomposition of transition densities into fragment-localized and charge-transfer contributions and provides quantitative insight into excited-state character.

To assess the sensitivity of the Gate I analysis to the choice of exchange–correlation functional, the Franck–Condon spectra, LIIC profiles, and spin–orbit couplings of $(Fv)Ru_2(CO)_4$ and $(Fv)OsW(CO)_5$ were additionally recomputed with the global hybrid B3LYP-D4 and the range-separated hybrids CAM-B3LYP-D4 and ωB97X-D4 (Section S9); the resulting state orderings, crossing topology, and SOC trends are consistent across all four functionals.

Excited-state potential-energy profiles were mapped along linearly interpolated internal coordinates (LIICs). For the Gate I analysis, the LIIC connects the optimized singlet LEI Franck–Condon (FC) geometry to the bond-cleaved region approaching the triplet biradical configuration; these reference geometries were obtained from B3LYP-D3(BJ)/def2-TZVPP/SDD calculations using GAUSSIAN 16.[S1] Quasi-diabatic tracking of the isomerizing triplet state along the LIIC was achieved by monitoring dominant orbital character together with state-specific SOCME fingerprints, allowing identification of the diabatically continuous triplet state correlated with bond cleavage even when adiabatic state ordering changed along the interpolation coordinate.

The reliability of the TDDFT description along the LIIC was assessed using the singlet broken-symmetry diagnostic $\langle \hat{S}^2 \rangle_{\mathrm{BS}}$. Interpretation of excited-state crossings and state re-orderings was restricted to regions where $\langle \hat{S}^2 \rangle_{\mathrm{BS}} \approx 0$, indicating negligible spin contamination and a predominantly single-reference electronic structure. Within the FC region, this criterion supports

the use of TDDFT/TDA for evaluating the energetic alignment of the bright singlet manifold and the isomerizing triplet state relevant to Gate I screening.

S3 Density Functional Benchmark for (Fv)$Ru_2(CO)_4$: GGA and Hybrid Rungs of Jacob's Ladder

To validate the density functional employed in this work, the reaction energetics of the benchmark system (Fv)$Ru_2(CO)_4$ were evaluated with several dispersion-corrected functionals spanning the GGA and hybrid rungs of Jacob's ladder and compared with experimental thermochemical data.[S14] Experimentally, the energy-storage reaction is characterized by a reaction enthalpy of $\Delta H_{\mathrm{storage}} = 19.8 \pm 1.4$ kcal mol^{-1}, while the thermal back reaction proceeds over a barrier of $\Delta G^{\ddagger}_{\mathrm{TS_{conc}}} = 25.6 \pm 0.5$ kcal mol^{-1}.

Along the reaction pathway the system evolves according to

$$\mathrm{HEI} \rightarrow \mathrm{TS_{conc}} \rightarrow \mathrm{ABR} \rightarrow \mathrm{TS_{rotn}} \rightarrow \mathrm{LEI}. \tag{4}$$

All computed energies are reported relative to HEI (set to zero), so the storage enthalpy is $\Delta H_{\mathrm{storage}} = H_{\mathrm{HEI}} - H_{\mathrm{LEI}}$ and the LEI minimum carries a negative relative energy.

Table S1: Computed reaction enthalpies (ΔH, kcal mol^{-1}) along the HEI → LEI pathway of (Fv)$Ru_2(CO)_4$, relative to HEI. Values in parentheses denote $\langle S^2 \rangle$. Bold entries mark the adopted B3LYP-D3(BJ) functional.

Functional (HF%)	HEI	$\mathrm{TS_{conc}}$	ABR	$\mathrm{TS_{rotn}}$	LEI
PBE-D3 (0%)	0.0 (0.0)	22.4 (0.0)	17.8 (0.0)	30.2 (1.0)	−17.0 (0.0)
BP86-D3 (0%)	0.0 (0.0)	23.2 (0.0)	19.5 (0.0)	31.7 (1.0)	−16.8 (0.0)
BP86-D3BJ (0%)	0.0 (0.0)	25.0 (0.0)	22.1 (0.0)	34.5 (1.0)	−16.7 (0.0)
B3LYP-D3 (20%)	0.0 (0.0)	23.5 (0.0)	15.6 (0.7)	21.4 (1.0)	−19.1 (0.0)
**B3LYP-D3BJ** (20%)	**0.0** (0.0)	**25.1** (0.0)	**18.5** (0.7)	**24.6** (1.0)	**−19.0** (0.0)
ωB97X-D (22%)	0.0 (0.0)	26.9 (0.5)	20.1 (0.9)	24.0 (1.0)	−17.4 (0.0)
PBE0-D3 (25%)	0.0 (0.0)	27.3 (0.0)	20.5 (0.8)	25.2 (1.0)	−17.7 (0.0)
PBE0-D3BJ (25%)	0.0 (0.0)	28.1 (0.0)	21.8 (0.8)	27.0 (1.0)	−17.7 (0.0)
Experiment					−19.8 ± 1.4

Computed LEI energies are reported relative to HEI (set to zero) and are therefore negative; the experimental storage enthalpy $|\Delta H_{\mathrm{storage}}| = 19.8$ kcal mol^{-1} is listed with matching sign convention for direct comparison with the computed LEI column.

Overall, the hybrid B3LYP-D3(BJ) functional provides the most balanced description of the reaction energetics. In particular, B3LYP-D3(BJ) reproduces the experimental barrier for the rate-determining $^1\mathrm{TS_{conc}}$ step ($\Delta G^{\ddagger}_{\mathrm{TS_{conc}}} = 24.6$ kcal mol^{-1} vs. 25.6 kcal mol^{-1} experimentally) while simultaneously yielding a storage enthalpy in excellent agreement with experiment ($\Delta H_{\mathrm{storage}} = 19.0$ kcal mol^{-1} vs. 19.8 kcal mol^{-1}). Although ωB97X-D reproduces the

Table S2: Computed reaction free energies (ΔG, kcal mol^{-1}) along the HEI → LEI pathway of (Fv)$Ru_2(CO)_4$, relative to HEI. Bold entries mark the adopted B3LYP-D3(BJ) functional; the rate-determining ^{1}TS$_{conc}$ barrier is the relevant comparison with experiment.

Functional (HF%)	HEI	TS$_{conc}$	ABR	TS$_{rotn}$	LEI
PBE-D3 (0%)	0.0	21.9	15.0	26.0	−17.3
BP86-D3 (0%)	0.0	22.7	16.8	27.5	−17.1
BP86-D3BJ (0%)	0.0	24.3	19.4	30.1	−17.1
B3LYP-D3 (20%)	0.0	23.2	12.5	19.4	−19.3
**B3LYP-D3BJ** (20%)	**0.0**	**24.6**	**15.0**	**22.3**	**−19.3**
ωB97X-D (22%)	0.0	25.4	18.4	21.5	−17.4
PBE0-D3 (25%)	0.0	26.6	16.9	23.2	−17.9
PBE0-D3BJ (25%)	0.0	27.4	18.1	24.9	−18.0
Experiment		25.6 ± 0.5			

^{1}TS$_{conc}$ barrier marginally more closely (25.4 kcal mol^{-1}), it underestimates the storage enthalpy by 2.4 kcal mol^{-1} (17.4 vs. 19.8 kcal mol^{-1}), whereas B3LYP-D3(BJ) matches both observables to within ~1 kcal mol^{-1} simultaneously. Pure GGA functionals systematically underestimate the storage enthalpy, whereas functionals with larger fractions of Hartree–Fock exchange tend to overestimate the activation barrier. Consequently, B3LYP-D3(BJ) was adopted for the ground-state mechanistic analysis presented in the main text.

S4 Assessment of Multireference Character: Fractional Occupation Number Weighted Density (FOD) Analysis

To assess whether static electron correlation could compromise the single-reference DFT protocol employed throughout this work, we quantified multireference character using the fractional occupation number weighted density (FOD) approach of Grimme and Hansen.[S15,S16] FOD calculations were performed for both the lower-energy isomer (LEI) and higher-energy isomer (HEI) of all 21 (Fv)MM′$(CO)_n$ dimers.

All calculations were carried out with ORCA 6.1[S10] at the TPSS-D3(BJ)[S4,S17] level using a Fermi smearing temperature of $T_{el} = 5000$ K. Scalar-relativistic effects were treated within the zeroth-order regular approximation (ZORA),[S12] employing the ZORA-def2-TZVPP basis set for the light atoms and segmented all-electron relativistically contracted SARC-ZORA-TZVPP basis sets[S18] for the transition metals, together with the SARC/J auxiliary basis. The calculations were performed on the B3LYP-D3(BJ)/def2-TZVPP/SDD optimized geometries described in Section S2. The resulting N_{FOD} values are collected in Table S3.

Two systematic trends emerge. First, in every system the LEI exhibits a larger N_{FOD} value than the corresponding HEI. This identifies the M−M′ σ bond present in the LEI as the primary locus of static correlation. Upon photoisomerization to the HEI, where both metal centers

become anchored by covalent M−Cp bonds, this correlation is substantially reduced.

Second, N_{FOD} decreases systematically down the $3d/4d/5d$ series. All Ru- and Os-containing complexes exhibit $N_{\mathrm{FOD}} \leq 0.45$, indicating only modest static correlation. As observed for the storage energetics discussed in the main text, heterobimetallic systems generally display intermediate N_{FOD} values relative to their homobimetallic limits, with the Group 8 metal significantly attenuating the static correlation associated with the Group 6 partner.

For context, the reference scale established by Grimme and Hansen[S15] places typical closed-shell organic molecules at $N_{\mathrm{FOD}} \lesssim 0.2$, whereas genuinely multireference systems such as singlet *p*-benzyne or MnO_4^- exhibit substantially larger values accompanied by spatially delocalized FOD densities. Many well-behaved closed-shell organometallic compounds, including ferrocene, occupy an intermediate regime characterized by relatively small, metal-localized FOD densities for which single-reference DFT methods have generally proven reliable. With the exception of the Cr_2 LEI, all systems considered here fall within this intermediate regime (N_{FOD} = 0.12–0.76), with the systematic LEI→HEI reduction confirming that the static correlation is confined to the M−M′ bonding region rather than pervading the entire electronic structure.

The sole exception is the Cr_2 LEI, for which $N_{\mathrm{FOD}} = 1.26$. This value approaches the regime typically associated with strongly multiconfigurational electronic structures and is consistent with the well-established multireference nature of Cr−Cr bonding.[S19–S21] The energetics reported for Cr_2 should therefore be regarded as qualitative. This system lies far from the viability frontier (Figure 2b–d of the main text) and is not advanced as a viable MOST candidate. Moreover, the elevated FOD value is confined to the LEI, whereas the corresponding HEI exhibits a much smaller value ($N_{\mathrm{FOD}} = 0.35$).

Notably, the frontier candidates display among the lowest N_{FOD} values in the entire library. For example, OsW exhibits $N_{\mathrm{FOD}} = 0.355$ (LEI) and 0.284 (HEI), comparable to the experimentally benchmarked (Fv)$Ru_2(CO)_4$ system itself ($N_{\mathrm{FOD}} = 0.255$ and 0.149 for LEI and HEI, respectively). These results indicate that the frontier candidates occupy a similar electronic-structure regime to the benchmark complex for which the underlying DFT protocol reproduces the available experimental thermochemistry and kinetics, thereby supporting the transferability of the computational framework employed here.

Finally, all stationary points investigated in this work, including biradicaloid intermediates and transition states, were treated within the broken-symmetry unrestricted Kohn–Sham DFT (BS-UDFT) formalism with approximate spin projection (Section S2). This treatment explicitly accounts for the dominant left–right correlation channel associated with σ^2/σ^{*2} mixing identified by the FOD analysis.

Table S3: Fractional occupation number weighted electron counts, N_{FOD}, for the lower-energy isomer (LEI, single M−M′ bond) and higher-energy isomer (HEI, two M−Cp bonds) of all 21 (Fv)MM′(CO)$_n$ dimers, computed at the scalar-relativistic TPSS-D3(BJ)/ZORA-def2-TZVPP(SARC-ZORA-TZVPP) level (T_{el} = 5000 K).

Dimer	LEI	HEI	Dimer	LEI	HEI
Cr_2	1.257	0.354	FeMo	0.568	0.348
Mo_2	0.648	0.411	RuCr	0.443	0.236
W_2	0.637	0.428	RuMo	0.395	0.277
Fe_2	0.499	0.280	RuW	0.406	0.283
Ru_2	0.255	0.149	RuFe	0.382	0.217
Os_2	0.220	0.121	OsCr	0.378	0.247
CrMo	0.761	0.378	OsMo	0.345	0.274
CrW	0.755	0.399	OsW	0.355	0.284
MoW	0.654	0.411	OsFe	0.355	0.215
FeCr	0.687	0.308	RuOs	0.228	0.120
FeW	0.567	0.360			

S5 Practical Metrics: Gravimetric Energy Storage Density

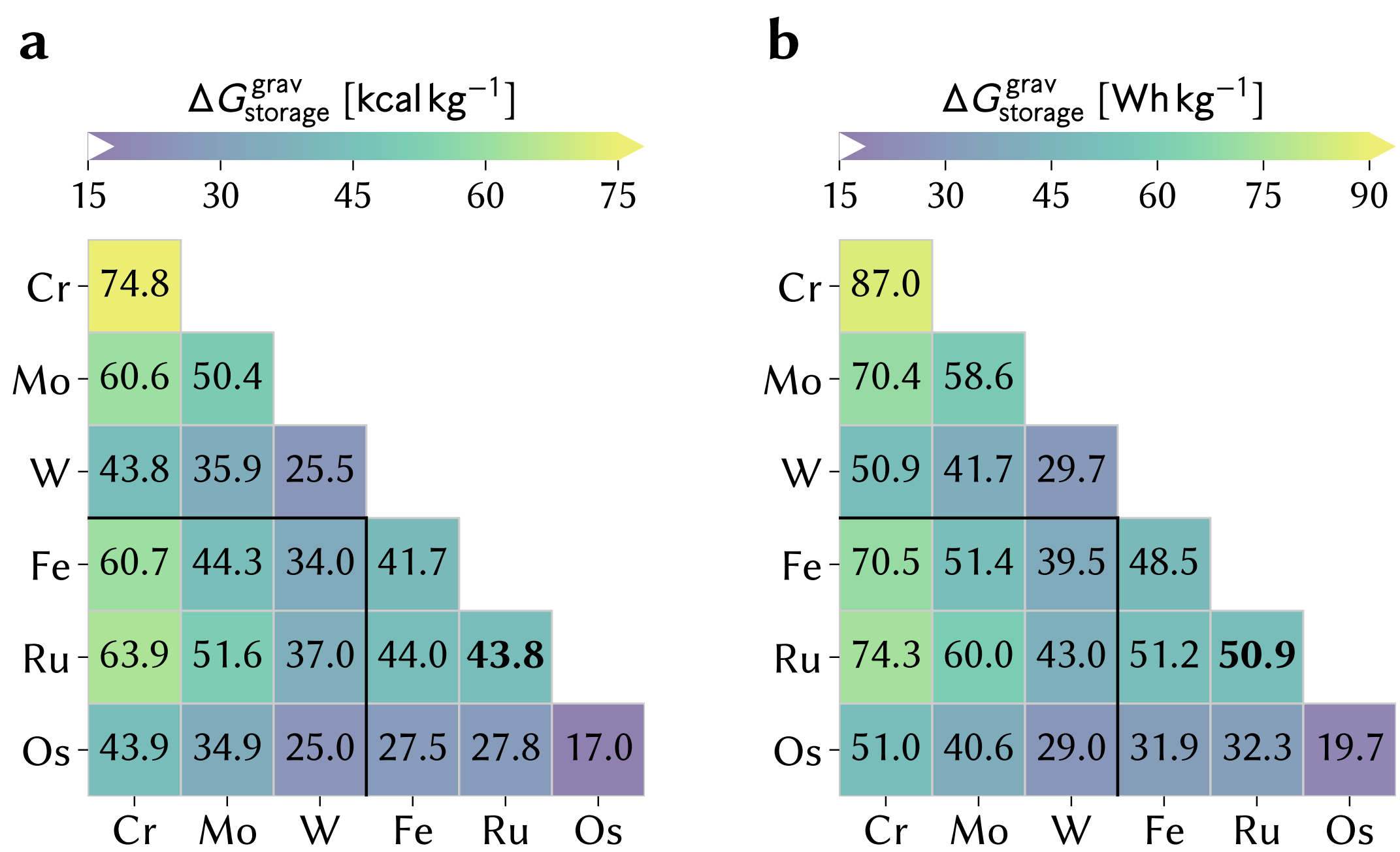

Figure S2. **Gravimetric storage energy of the 21 fulvalene-bridged bimetallic MOST candidates.** Storage free energy normalized by molecular weight, $\Delta G^{\text{grav}}_{\text{storage}} = \Delta G_{\text{storage}}/M_{\text{w}}$, shown as a lower-triangular matrix ordered by Cr, Mo, W, Fe, Ru, and Os in two common units: **(a)** kcal kg^{-1} and **(b)** Wh kg^{-1}. Molecular weights are calculated from the corresponding fulvalene-carbonyl compositions (fulvalene $C_{10}H_6$ plus n CO, where $n = 6$, 5, and 4 for Group 6/6, Group 6/8, and Group 8/8 pairs, respectively). The benchmark (Fv)$Ru_2(CO)_4$ is highlighted in bold. Mass normalization favors lighter congeners: Cr_2 exhibits the highest gravimetric storage density, whereas Os_2 exhibits the lowest. The corresponding molar storage energies are shown in Figure 2b.

For a MOST molecular fuel, the gravimetric storage energy, $\Delta G^{\text{grav}}_{\text{storage}}$, connects the molecular-level storage thermodynamics with the practical energy-storage capacity. It quantifies the amount of stored energy available per unit mass of material and therefore determines the amount of molecular fuel required to deliver a given energy output. It follows directly from the storage free energy and the molecular weight of the photoswitch,

$$\Delta G^{\text{grav}}_{\text{storage}} = \frac{\Delta G_{\text{storage}}}{M_{\text{w}}}, \tag{5}$$

and is evaluated for all 21 candidates in Figure S2 using the two commonly used units kcal kg^{-1} and Wh kg^{-1}.

The gravimetric storage energies correlate strongly with the corresponding molar storage energies ($r \approx 0.89$; Figure S2), as expected because $\Delta G^{\text{grav}}_{\text{storage}}$ scales directly with $\Delta G_{\text{storage}}$. The correlation is not perfect as molecular weight introduces a second independent design parameter. The heavy $5d$ metals that enhance relativistic bonding interactions and over-stabilize the high-energy isomer (HEI) also contribute substantially larger molecular masses. Conse-

quently, heavy-metal substitution penalizes gravimetric storage density through two simultaneous effects: a reduced storage free energy in the numerator and an increased molecular weight in the denominator. This double penalty drives the heavy Os_2 and OsW systems toward the bottom of both the molar and gravimetric rankings, whereas the lighter Cr_2 analogue achieves the highest gravimetric value.

Importantly, this mass normalization does not alter the gate-based selection criteria, which are defined by the storage–stability window rather than by gravimetric density alone. Instead, it provides a practical perspective on the material-level consequences of metal substitution and highlights the tradeoff between favorable electronic structure and molecular mass.

The two gate-satisfying candidates illustrate this distinction directly. For the benchmark $(Fv)Ru_2(CO)_4$ ($\Delta G_{\text{storage}} = 19.3$ kcal mol^{-1}; $M_{\text{w}} = 440.3$ g mol^{-1}), Eq. (5) gives $\Delta G_{\text{storage}}^{\text{grav}} \approx$ 43.8 kcal kg^{-1} (50.9 Wh kg^{-1}). In comparison, for $(Fv)OsW(CO)_5$ ($\Delta G_{\text{storage}} = 16.0$ kcal mol^{-1}; $M_{\text{w}} = 640.3$ g mol^{-1}), the corresponding value is only $\Delta G_{\text{storage}}^{\text{grav}} \approx 25.0$ kcal kg^{-1} (29.0 Wh kg^{-1}). The lower gravimetric storage density of $(Fv)OsW(CO)_5$ therefore arises from two compounding factors: its smaller storage free energy (16.0 versus 19.3 kcal mol^{-1}) and the approximately 45% larger molecular mass of $(Fv)OsW(CO)_5$ relative to $(Fv)Ru_2(CO)_4$ (640.3 versus 440.3 g/mol). Consequently, the Ru benchmark provides approximately 75% higher gravimetric storage capacity than the Os–W analogue despite only a $\sim$ 3 kcal mol^{-1} difference in molar storage energy.

These gravimetric considerations, together with the greater elemental abundance, lower cost, and improved sustainability profile of Ru relative to Os and W, further favor the benchmark system for practical MOST applications. Accordingly, $(Fv)OsW(CO)_5$ is not proposed as a direct replacement for $(Fv)Ru_2(CO)_4$; rather, its importance lies in independently satisfying both mechanistic gates, thereby validating the dual-gate framework as a predictive strategy for identifying promising bimetallic MOST candidates.

S6 Gate II: Thermal Back-Reaction Potential-Energy Surfaces of Fulvalene-Bridged Bimetallic Complexes

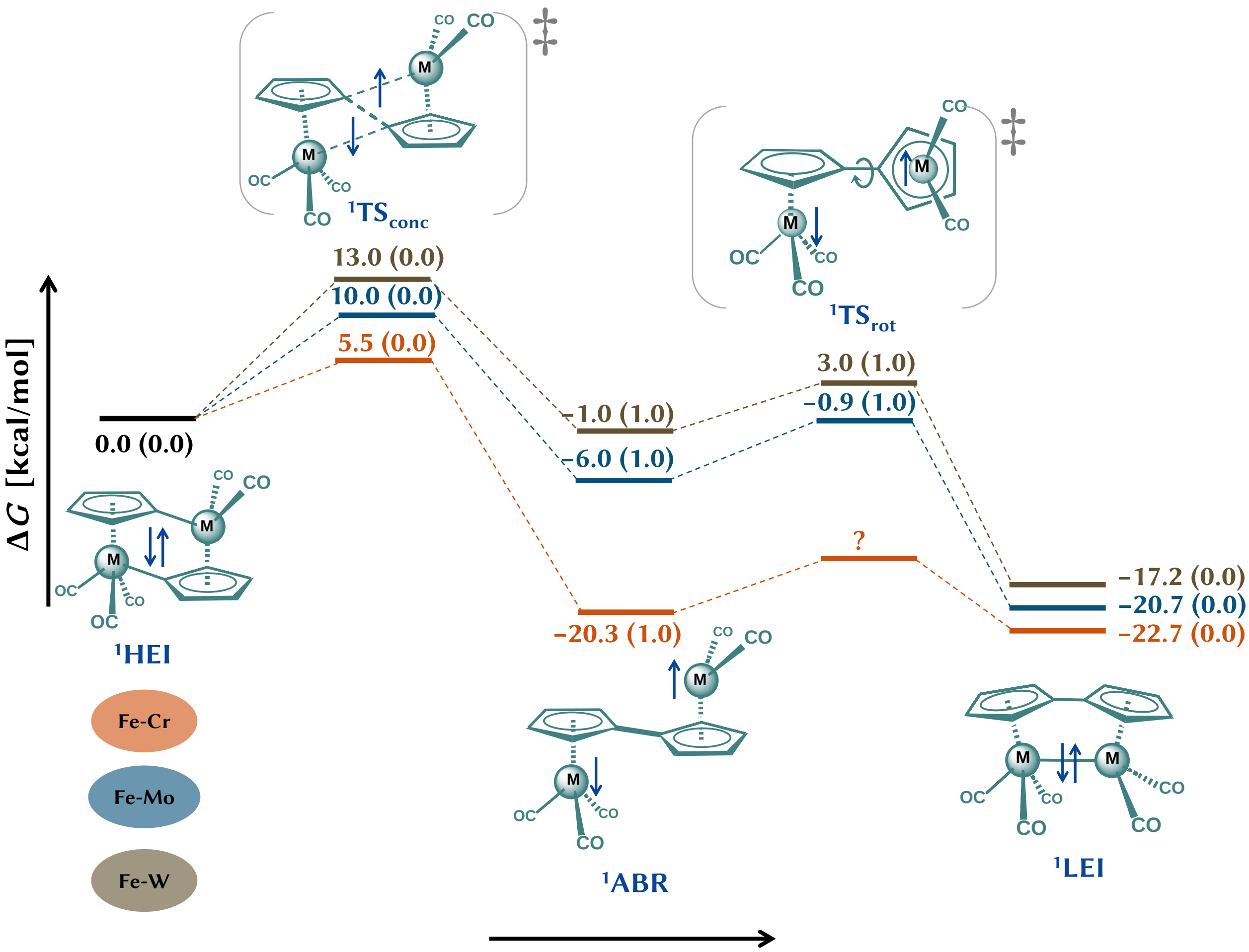

Figure S3. Yamaguchi-corrected free-energy surfaces for the Group 6/Group 8 heterobimetallic complexes (Fv)FeCr$(CO)_5$, (Fv)FeMo$(CO)_5$, and (Fv)FeW$(CO)_5$ (distinguished by color), mapped along the ^{1}HEI→^{1}LEI storage pathway *via* $^1TS_{conc}$, ^{1}ABR, and $^1TS_{rot}$. Values in parentheses report $\langle \hat{S}^2 \rangle_{BS}$.

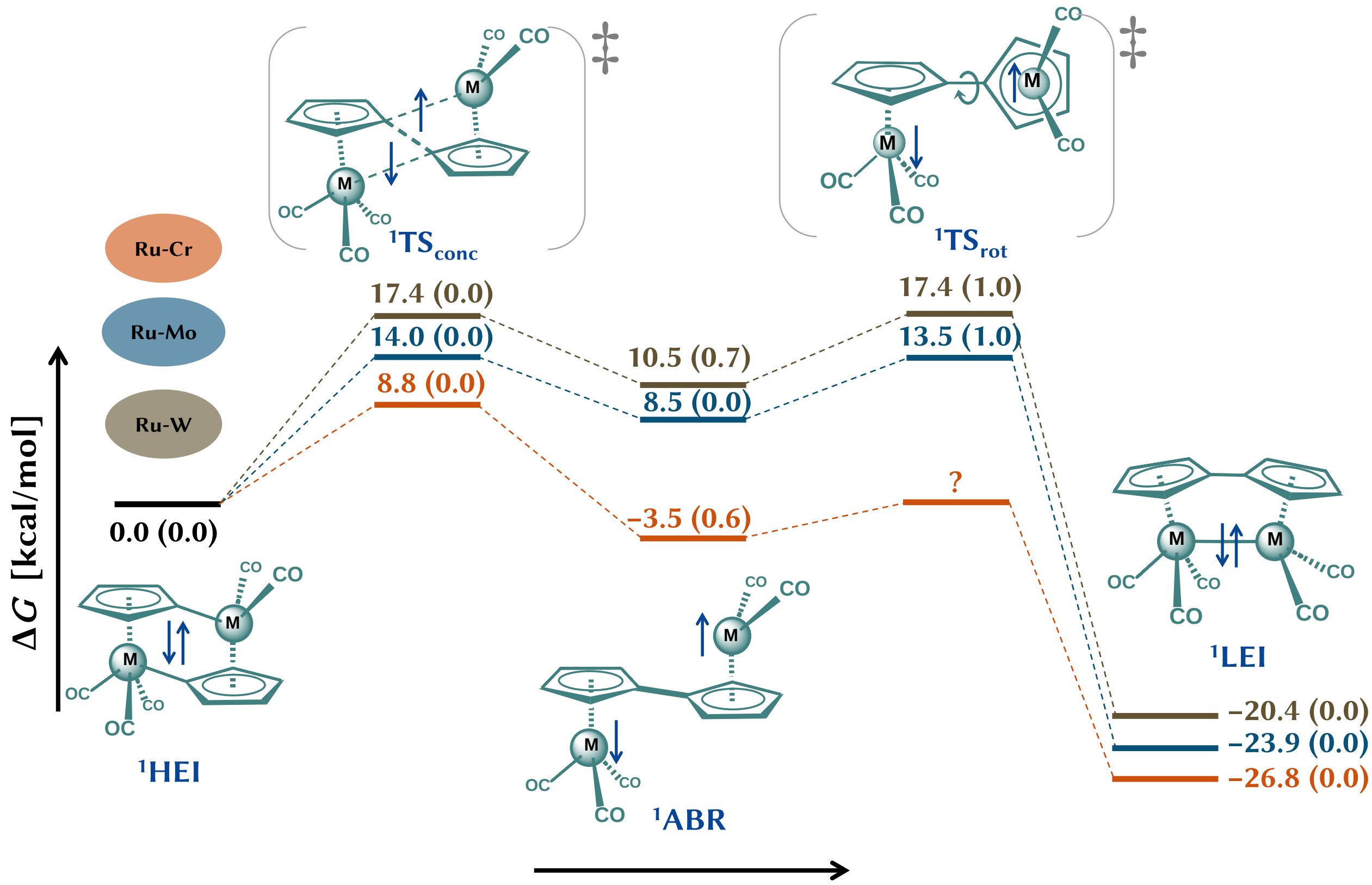

Figure S4. Yamaguchi-corrected free-energy surfaces for the Group 6/Group 8 heterobimetallic complexes (Fv)RuCr$(CO)_5$, (Fv)RuMo$(CO)_5$, and (Fv)RuW$(CO)_5$ (distinguished by color), mapped along the ^{1}HEI→^{1}LEI storage pathway *via* $^1TS_{conc}$, ^{1}ABR, and $^1TS_{rot}$. Values in parentheses report $\langle \hat{S}^2 \rangle_{BS}$.

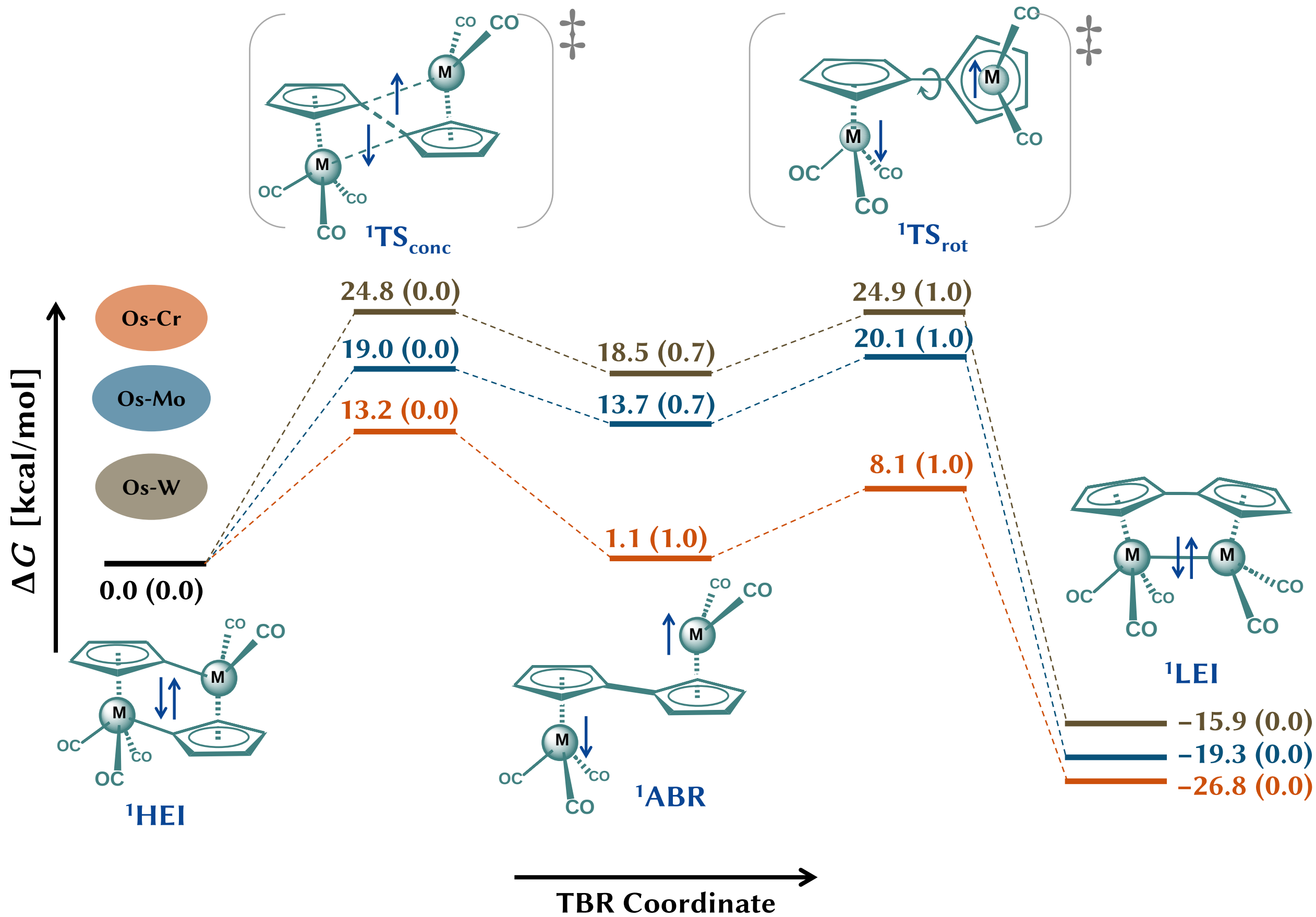

Figure S5. Yamaguchi-corrected free-energy surfaces for the Group 6/Group 8 heterobimetallic complexes (Fv)OsCr$(CO)_5$, (Fv)OsMo$(CO)_5$, and (Fv)OsW$(CO)_5$ (distinguished by color), mapped along the ^{1}HEI→^{1}LEI storage pathway *via* $^1TS_{conc}$, ^{1}ABR, and $^1TS_{rot}$. Values in parentheses report $\langle \hat{S}^2 \rangle_{BS}$.

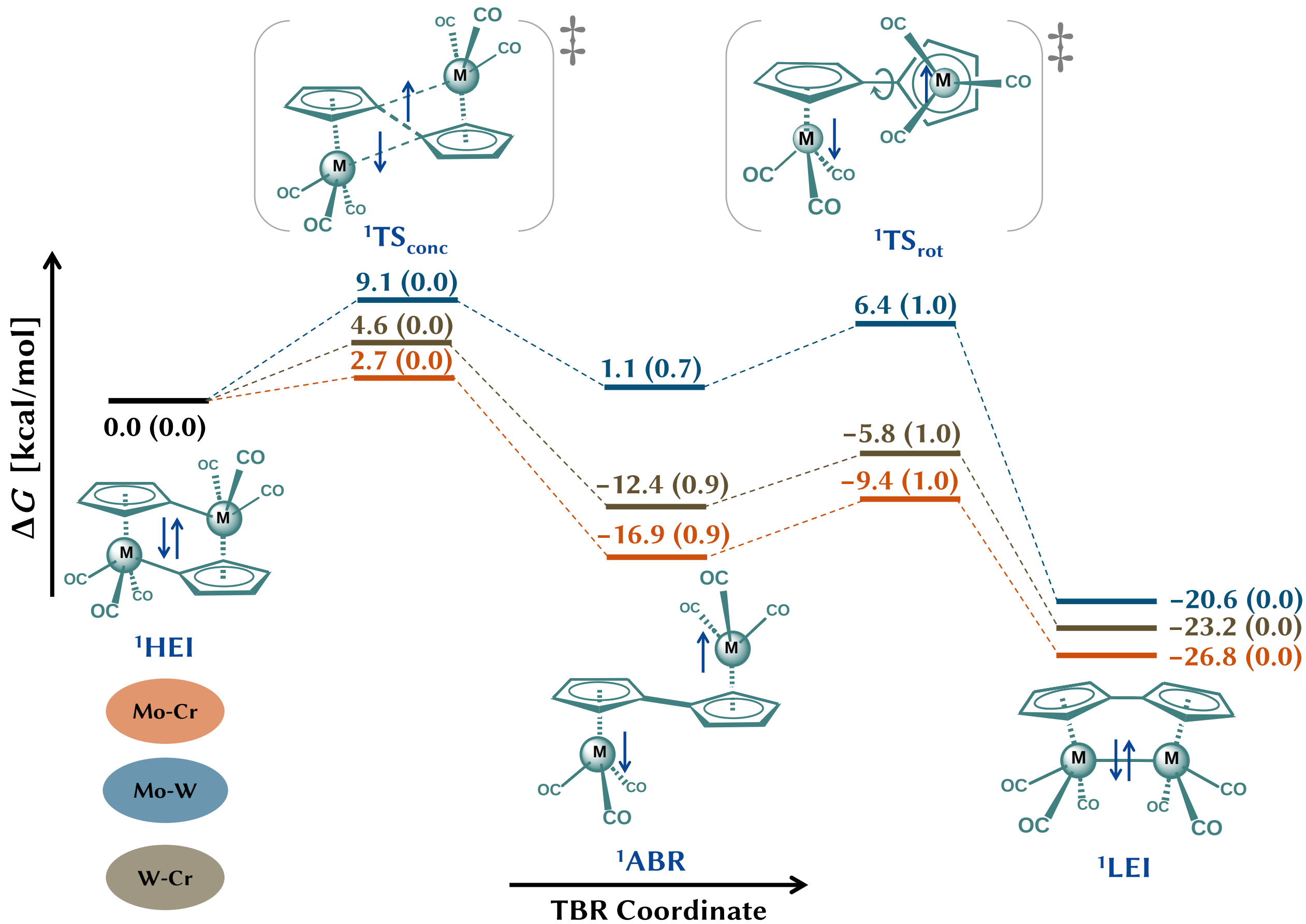

Figure S6. Yamaguchi-corrected free-energy surfaces for the Group 6 heterobimetallic complexes (Fv)MoCr$(CO)_6$, (Fv)MoW$(CO)_6$, and (Fv)WCr$(CO)_6$ (distinguished by color), mapped along the ^{1}HEI→^{1}LEI storage pathway *via* ^{1}TS$_{conc}$, ^{1}ABR, and ^{1}TS$_{rot}$. Values in parentheses report $\langle \hat{S}^2 \rangle_{BS}$.

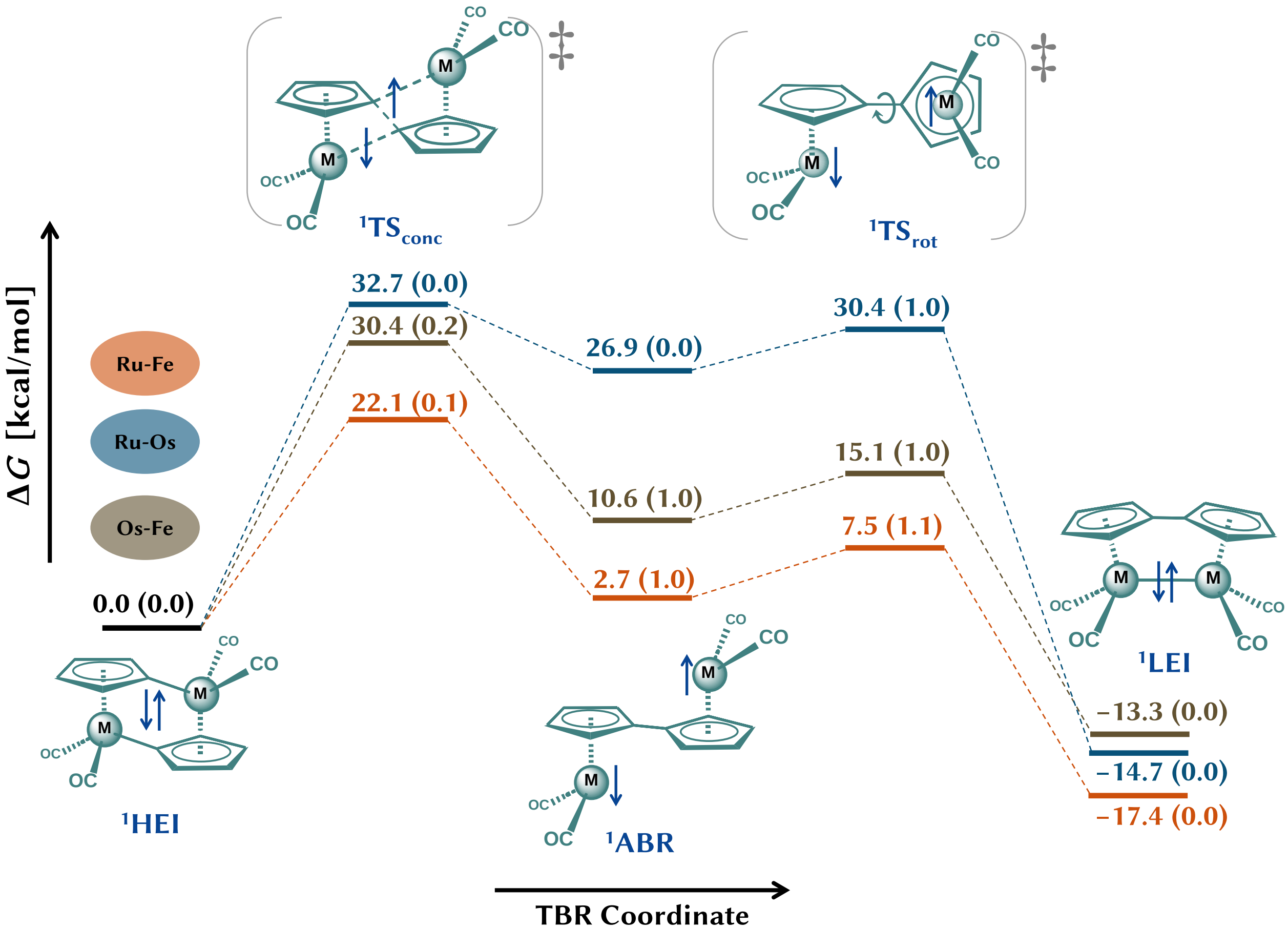

Figure S7. Yamaguchi-corrected free-energy surfaces for the Group 8 heterobimetallic complexes (Fv)RuFe$(CO)_4$, (Fv)RuOs$(CO)_4$, and (Fv)OsFe$(CO)_4$ (distinguished by color), mapped along the ^{1}HEI→^{1}LEI storage pathway *via* ^{1}TS$_{conc}$, ^{1}ABR, and ^{1}TS$_{rot}$. Values in parentheses report $\langle \hat{S}^2 \rangle_{BS}$.

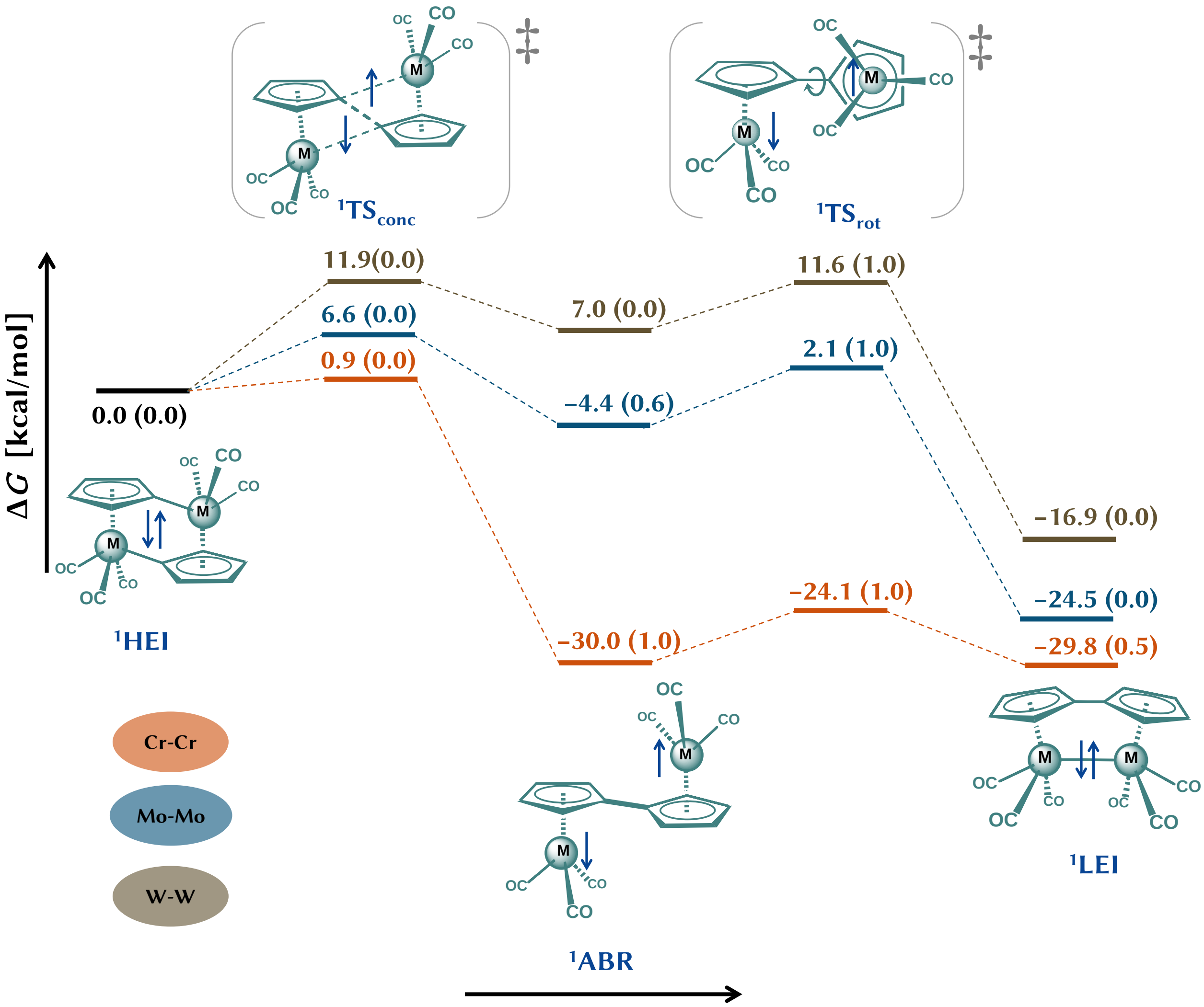

Figure S8. Yamaguchi-corrected free-energy surfaces for the Group 6 homobimetallic complexes $(Fv)Cr_2(CO)_6$, $(Fv)Mo_2(CO)_6$, and $(Fv)W_2(CO)_6$ (distinguished by color), mapped along the ^{1}HEI→^{1}LEI storage pathway *via* $^1TS_{conc}$, ^{1}ABR, and $^1TS_{rot}$. Values in parentheses report $\langle \hat{S}^2 \rangle_{BS}$.

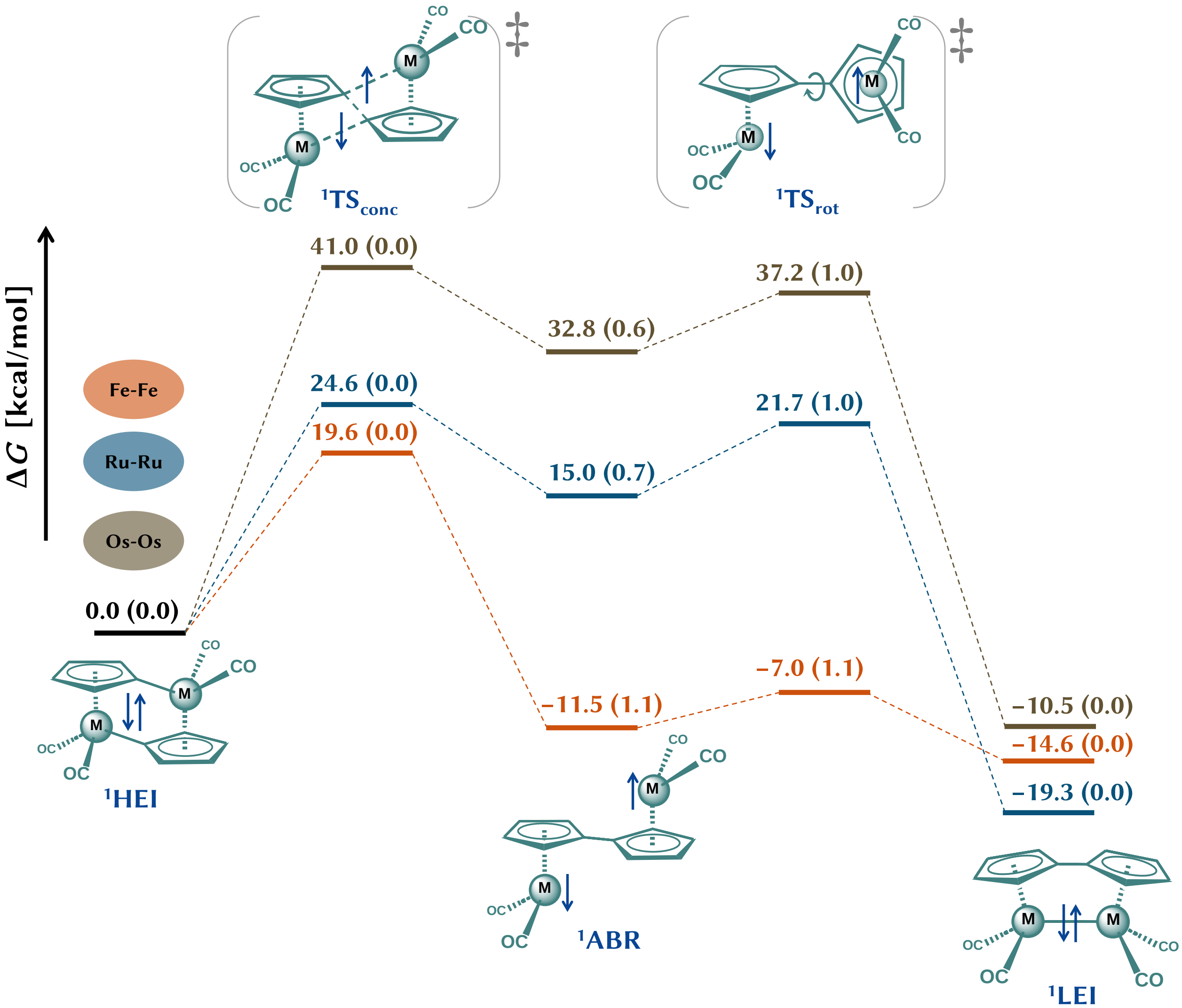

Figure S9. Yamaguchi-corrected free-energy surfaces for the Group 8 homobimetallic complexes $(Fv)Fe_2(CO)_4$, $(Fv)Ru_2(CO)_4$, and $(Fv)Os_2(CO)_4$ (distinguished by color), mapped along the ^{1}HEI→^{1}LEI storage pathway *via* ^{1}TS$_{conc}$, ^{1}ABR, and ^{1}TS$_{rot}$. Values in parentheses report $\langle \hat{S}^2 \rangle_{BS}$.

S7 Regression and Correlation Statistics for the Storage–Stability Tradeoff

The storage–stability tradeoff between $\Delta G_{\text{storage}}$ and $\Delta G^{\ddagger}_{\text{TBR}}$ (main text, Figure 2d) was quantified by ordinary least-squares regression using the linear model $\Delta G_{\text{storage}} = a + m\,\Delta G^{\ddagger}_{\text{TBR}}$, fitted to the complete library of $N = 21$ dimers and, separately, to the homobimetallic ($N = 6$) and heterobimetallic ($N = 15$) subsets. Standard errors for the slope and intercept were obtained from the least-squares covariance matrix, and 95% confidence intervals for the slope were estimated by bootstrap resampling (10^4 resamples). The covariance- and bootstrap-based uncertainty estimates are in close agreement for the complete data set and differ only for the small homobimetallic subset, whose confidence interval is correspondingly wider. In addition to the coefficient of determination (R^2), we report the Spearman rank correlation coefficient (ρ), which quantifies the strength of the monotonic association without assuming a strictly linear relationship, together with its two-sided p-value.

Table S4: Linear regression and correlation statistics for the storage–stability tradeoff (Figure 2d), obtained using the linear model $\Delta G_{\text{storage}} = a + m\,\Delta G^{\ddagger}_{\text{TBR}}$. Energies are reported in kcal mol^{-1}. Slope (m) and intercept (a) uncertainties are one standard error (SE); the 95% confidence interval (CI) for m was estimated by bootstrap resampling (10^4 resamples). RMSE is the root-mean-square residual about the fit (n-normalized). ρ is the Spearman rank correlation coefficient, with the corresponding two-sided p-value.

Subset	N	$m \pm$ SE	95% CI on m	$a \pm$ SE	R^2	RMSE	ρ (p)
All	21	-0.40 ± 0.06	(-0.51, -0.30)	26.54 ± 1.23	0.67	2.7	-0.80 (1.2×10^{-5})
Homo	6	-0.43 ± 0.15	(-1.12, -0.18)	27.11 ± 3.27	0.68	3.6	-0.83 (4.2×10^{-2})
Hetero	15	-0.38 ± 0.07	(-0.50, -0.27)	26.17 ± 1.32	0.67	2.3	-0.80 (3.5×10^{-4})

The similar regression parameters obtained for the complete, homobimetallic, and heterobimetallic data sets indicate that the storage–stability tradeoff is not driven by a particular chemical subclass but is instead a general feature of the screened fulvalene-bridged bimetallic library. Importantly, however, this correlation plays a descriptive rather than predictive role: it establishes that a common bond-strength descriptor governs both storage thermodynamics and release kinetics across the library, whereas candidate selection in Gate II rests on membership in the ($\Delta G^{\ddagger}_{\text{TBR}}$, $\Delta G_{\text{storage}}$) storage–stability window rather than on the regression itself.

S8 Gate I: TDDFT Analysis of Comparator Bimetallic Systems

To contextualize the two-gate screening results, TDDFT analyses were performed for additional comparator systems illustrating the two principal non-viability modes: (i) Gate I-passing compounds that fall just outside the Gate II daily-use window, and (ii) systems where the excited-state alignment suppresses productive ISC despite substantial SOC.

S8.1 (Fv)OsMo(CO)$_5$: Gate I Pass, Gate II Near-Miss

The heterobimetallic complex (Fv)OsMo(CO)$_5$ closely follows the Ru_2/OsW subclass identified in the main text. At the FC geometry the Os−−Mo distance is 3.08 Å, increasing to 4.17 Å at the triplet biradical ^{3}SBR, with a torsional opening to $\phi = 21.9°$ (RMSD ≈ 0.88 Å).

The FC absorption spectrum (Figure S10a) shows a singlet–triplet energy pattern similar to Ru_2 and OsW, with metal-centered excitations dominating the low-lying manifold. Along the LIIC (Figure S10b,c), the excited-state topology reveals the same spin-gated mechanism. Substantial SOCMEs are already present at the FC geometry ($\langle S_1|\hat{H}_{SO}|T_2\rangle \approx 280\ \mathrm{cm}^{-1}$), indicating efficient access to the isomerizing triplet.

Despite satisfying Gate I, (Fv)OsMo(CO)$_5$ lies just below the Gate II daily-use window owing to a marginally insufficient thermal back-reaction barrier ($\Delta G_{TBR} = 21.3$ kcal mol^{-1}), corresponding to an estimated half-life shorter than one day.

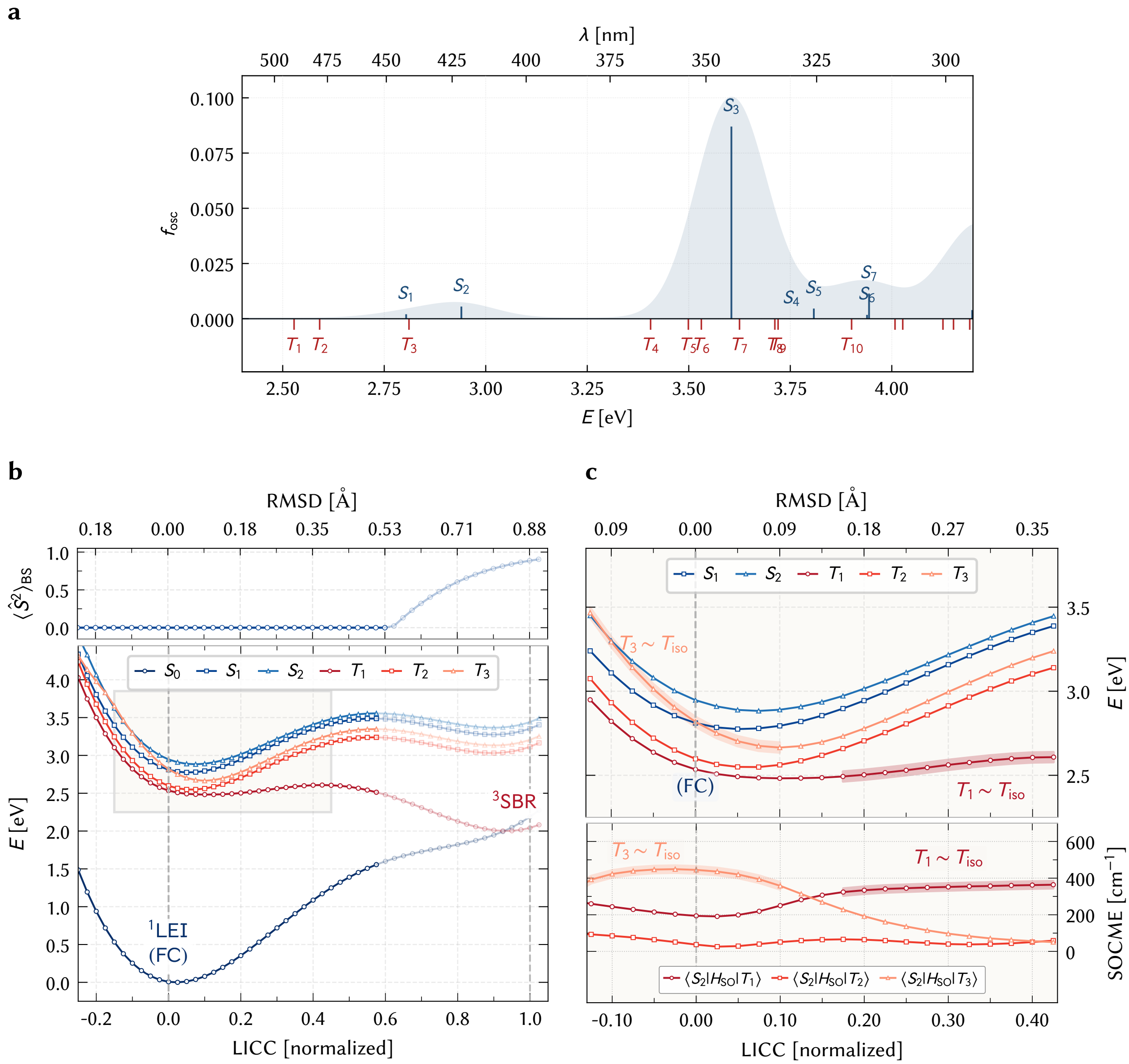

Figure S10. Gate I excited-state analysis of $(Fv)OsMo(CO)_5$ at the PBE0-D4 level. (**a**) FC absorption spectrum. (**b**) $\langle\hat{S}^2\rangle_{BS}$ profile and adiabatic singlet/triplet LIIC from ^{1}LEI to ^{3}SBR, with the RMSD-based structural scale. (**c**) Expanded FC region tracking S_1, S_2, T_1–T_3, T_{iso}, and the corresponding SOCMEs. The panel layout mirrors that of Ru_2 and OsW, confirming that $(Fv)OsMo(CO)_5$ follows the same spin-gated photochemical motif.

S8.2 Fe-Containing Dimers: Intrinsic Gate I Failure

The Fe-containing dimers (Fv)RuFe$(CO)_4$ and (Fv)OsFe$(CO)_4$ represent a qualitatively distinct subclass. Both begin from short M−−Fe separations at the FC geometry (2.78 Å), with the corresponding ^{3}SBR structures showing only modest elongation to 3.52 Å (RuFe) and 3.40 Å (OsFe) while remaining nearly planar ($\phi \approx 0°$). This structural response reflects the weaker ligand field at the 3d Fe center and is accompanied by a markedly higher density of low-lying triplet states.

The FC absorption spectra (Figure S11a and Figure S12a) show dense triplet manifolds lying significantly below the bright singlet states. LIIC analyses (Figure S11b and Figure S12b) reveal that the critical S^*/T_{iso} crossing is displaced into the Marcus-inverted regime in both complexes. Although SOCMEs > 200 cm^{-1} are present at the FC geometry, the unfavorable energetic alignment suppresses ISC into the reactive triplet channel. This behavior rationalizes the experimentally observed failure of (Fv)RuFe$(CO)_4$ to undergo photocharging[S22] and indicates that Fe incorporation leads to intrinsic Gate I failure within this fulvalene architecture.

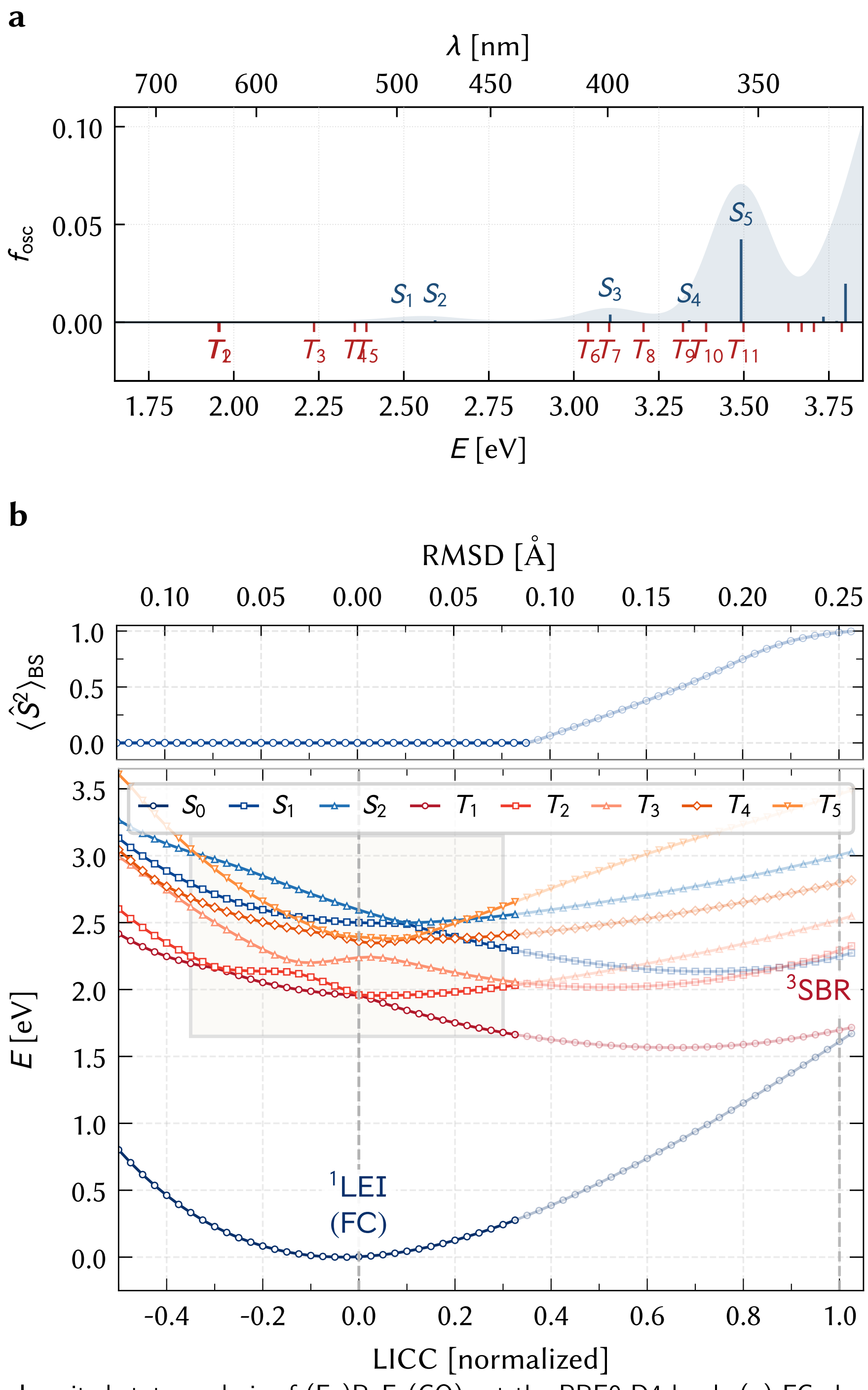

Figure S11. Gate I excited-state analysis of $(Fv)RuFe(CO)_4$ at the PBE0-D4 level. (**a**) FC absorption spectrum. (**b**) $\langle \hat{S}^2 \rangle_{BS}$ profile and adiabatic singlet/triplet LIIC from ^{1}LEI to ^{3}SBR, with the RMSD-based structural scale.

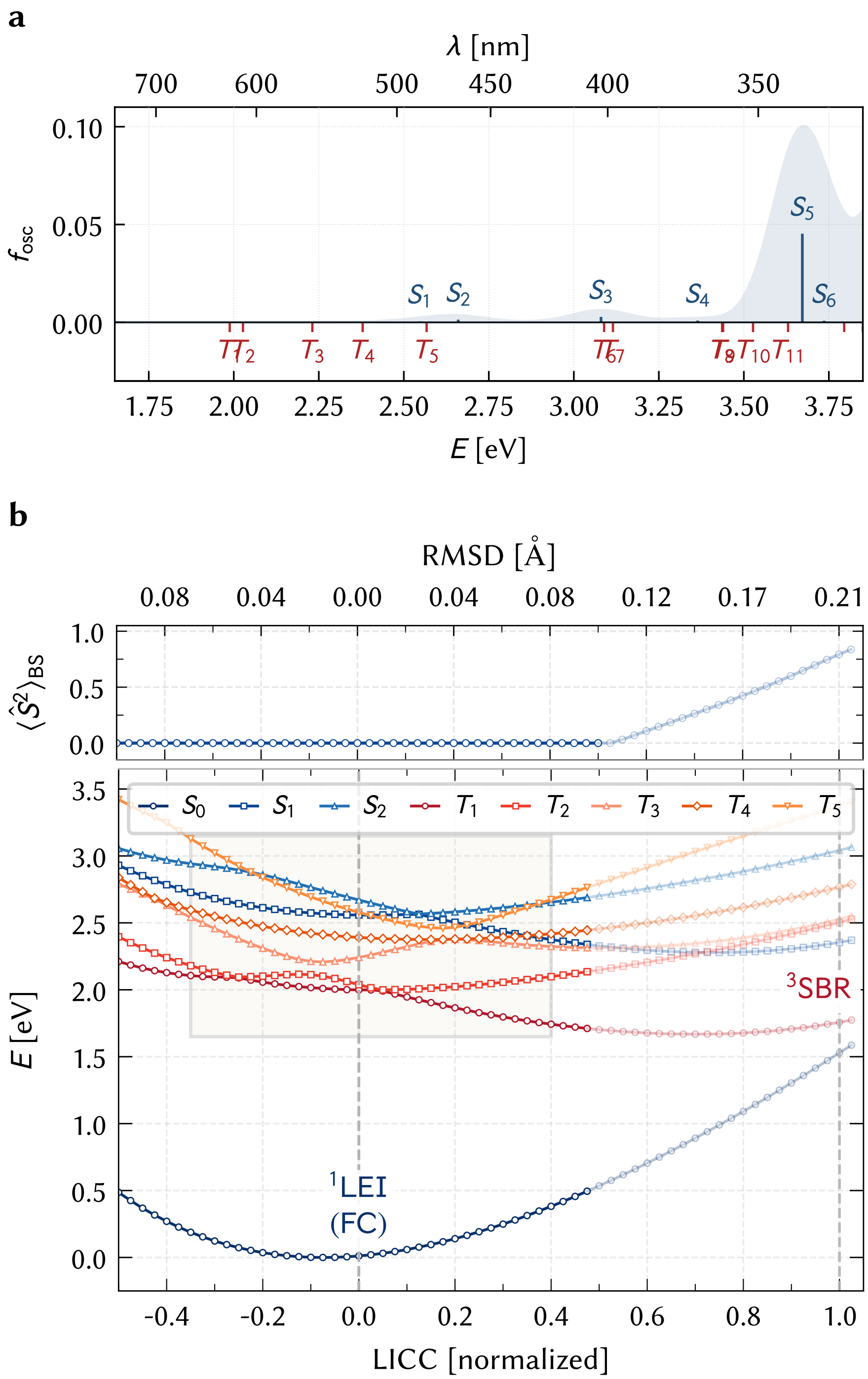

Figure S12. Gate I excited-state analysis of $(Fv)OsFe(CO)_4$ at the PBE0-D4 level. (**a**) FC absorption spectrum. (**b**) $\langle \hat{S}^2 \rangle_{BS}$ profile and adiabatic singlet/triplet LIIC from ^{1}LEI to ^{3}SBR, with the RMSD-based structural scale.

S9 Gate I: TDDFT Benchmark of (Fv)$Ru_2(CO)_4$ and (Fv)OsW$(CO)_5$ — Franck–Condon Absorption, LIIC Topology, and Spin–Orbit Coupling

In this section we benchmark TDDFT for evaluating the Franck–Condon absorption spectra, the topology along linearly interpolated internal coordinates (LIICs), and the spin–orbit coupling (SOC) and intersystem crossing (ISC) accessibility for the two benchmark systems.

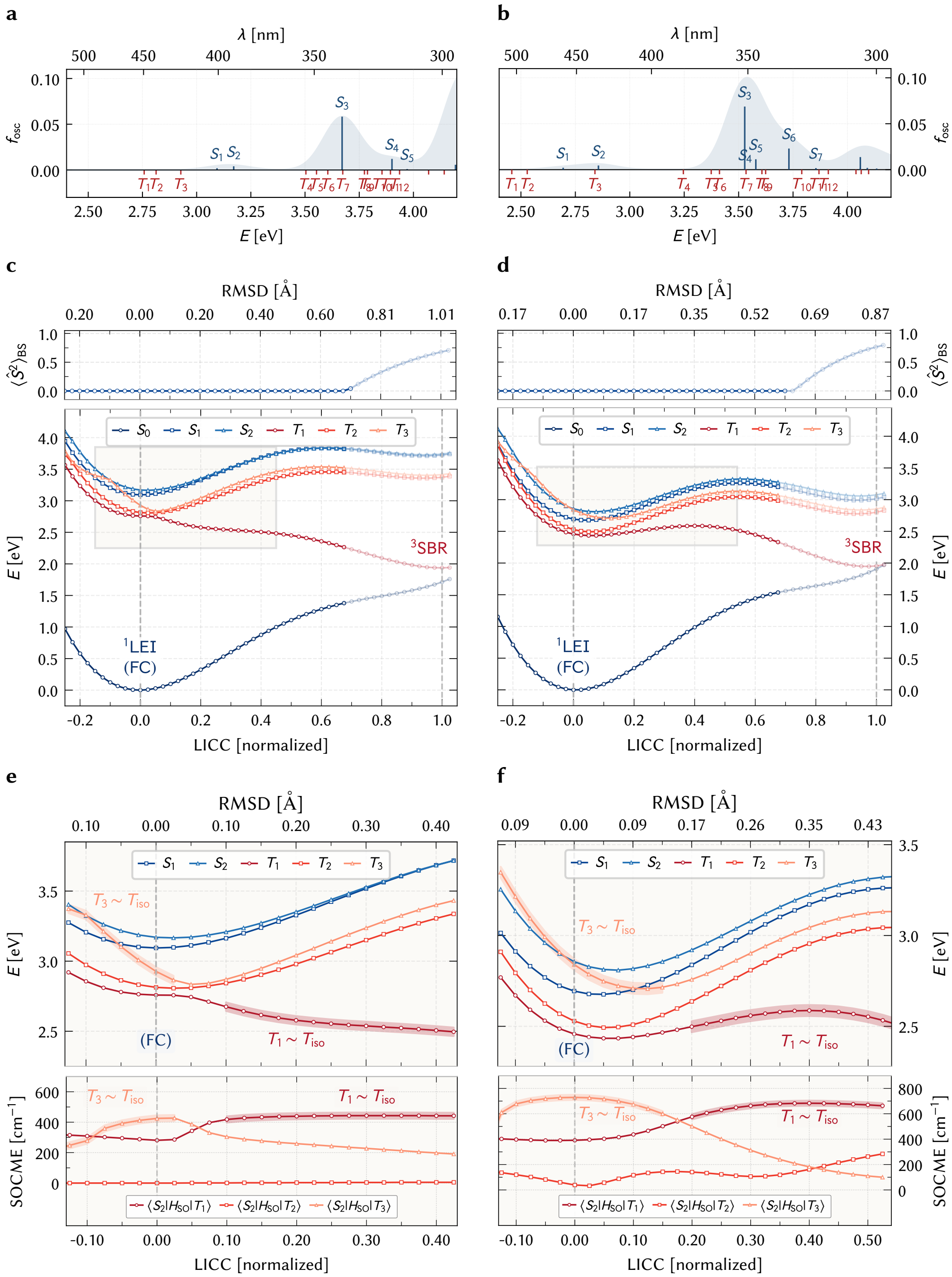

Figure S13. Gate I benchmark with TDDFT/B3LYP-D4 for $(Fv)Ru_2(CO)_4$ and $(Fv)OsW(CO)_5$. The panels correspond to those in the main-text figure, now at the B3LYP-D4 level: (**a**,**b**) FC absorption spectra; (**c**,**d**) adiabatic singlet and triplet LIIC profiles from ^{1}LEI to ^{3}SBR, with RMSD and $\langle \hat{S}^2 \rangle_{BS}$ diagnostics; (**e**,**f**) expanded FC region showing S_1, S_2, T_1–T_3, the isomerizing triplet T_{iso}, and the corresponding SOCMEs. Trends are consistent with the PBE0-D4 reference.

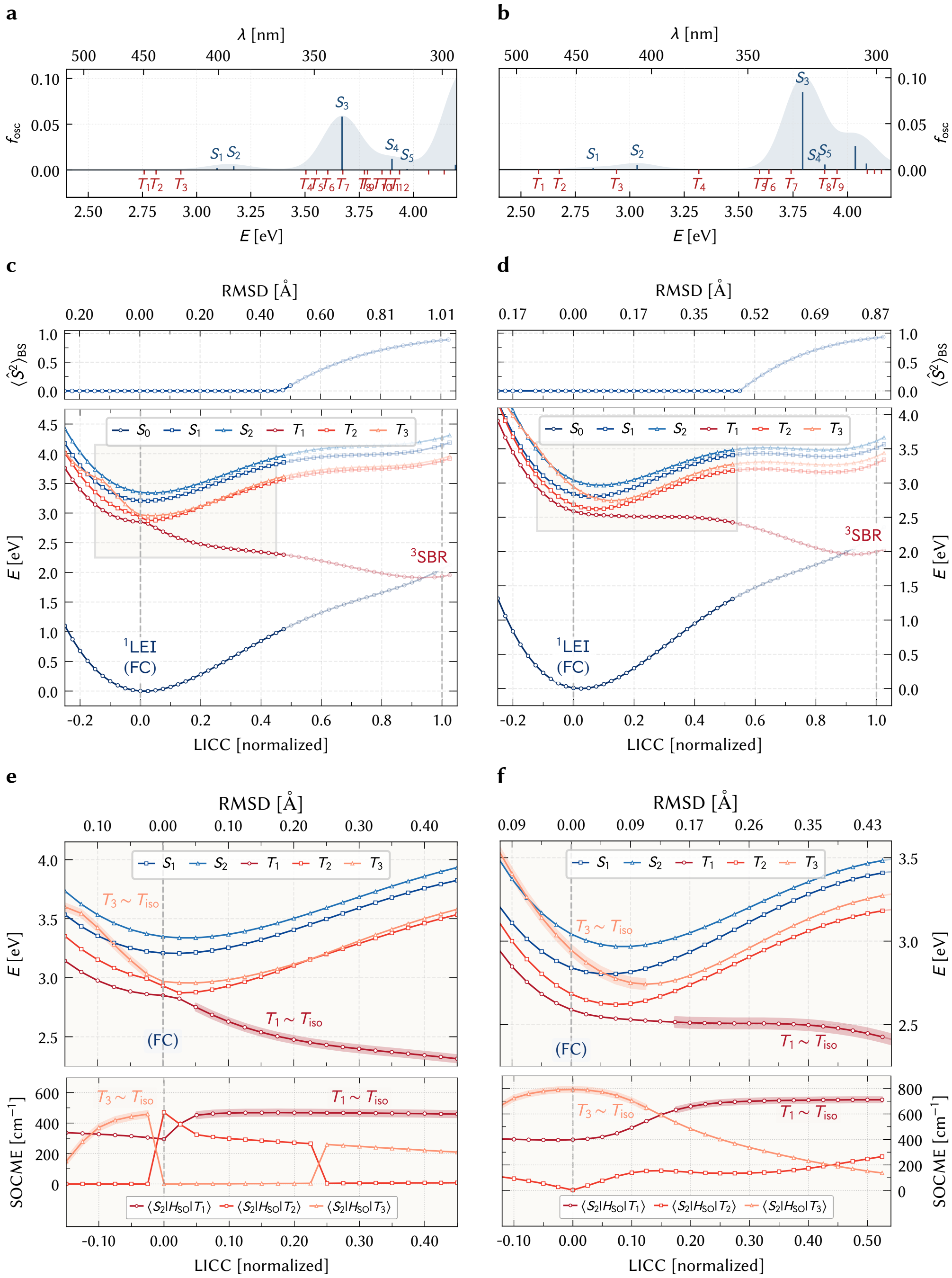

Figure S14. Gate I benchmark with TDDFT/CAM-B3LYP-D4 for (Fv)$Ru_2(CO)_4$ and (Fv)OsW$(CO)_5$. Panel layout as in Figure S13: (**a**,**b**) FC absorption spectra; (**c**,**d**) adiabatic LIIC profiles and $\langle \hat{S}^2 \rangle_{BS}$ diagnostics; (**e**,**f**) FC-region evolution of S_1, S_2, T_1–T_3, T_{iso}, and SOCMEs. CAM-B3LYP-D4 corroborates the qualitative PBE0-D4 picture.

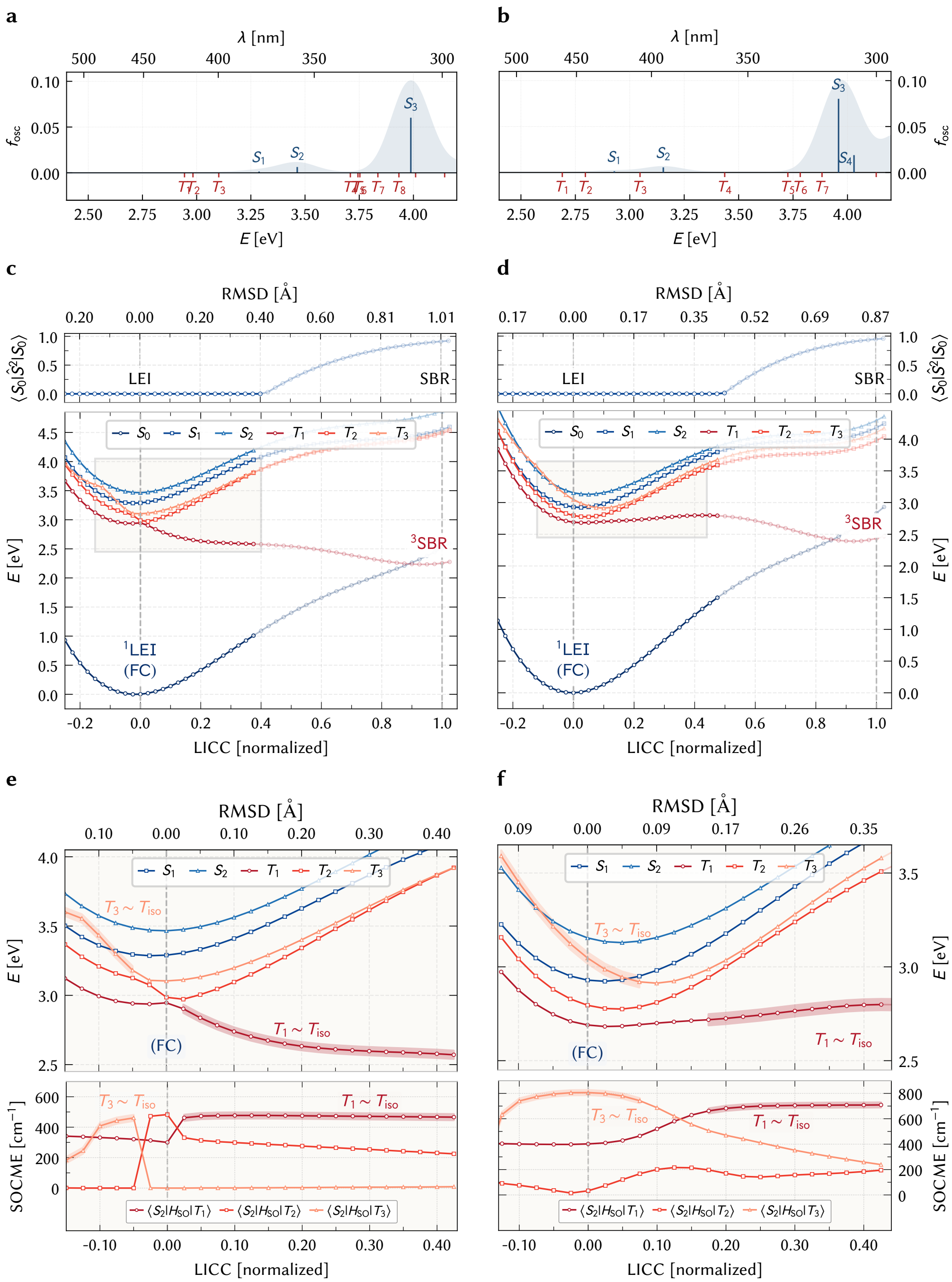

Figure S15. Gate I benchmark with TDDFT/ωB97X-D4 for $(Fv)Ru_2(CO)_4$ and $(Fv)OsW(CO)_5$. Panel layout as in Figure S13: (**a**,**b**) FC absorption spectra; (**c**,**d**) LIIC profiles and $\langle \hat{S}^2 \rangle_{BS}$ diagnostics; (**e**,**f**) FC-region details for S_1, S_2, T_1–T_3, T_{iso}, and SOCMEs. The ωB97X-D4 results confirm robustness of the key mechanistic features with respect to the choice of hybrid functional.

S10 Gate I: Natural Transition Orbital Analysis

Natural transition orbital (NTO) analysis was performed to characterize the electronic nature of the low-lying excited states involved in Gate I screening. NTOs provide a compact representation of electronic excitations in terms of dominant hole–particle orbital pairs, allowing direct identification of the metal-centered σ/π excitations discussed in the main text.

For the homobimetallic benchmark (Fv)$Ru_2(CO)_4$, the excited states at the FC geometry are symmetry-delocalized over both Ru centers. Within the σ/π framework of the main text,[S23] the lowest singlet excitations correspond to $\pi^*_\perp \rightarrow \sigma^*$ (S_1) and $\pi^*_\parallel \rightarrow \sigma^*$ (S_2) promotions, with T_1 and T_2 reflecting the same orbital character and T_3 dominated by $\sigma \rightarrow \sigma^*$.

For the heterobimetallic complex (Fv)OsW$(CO)_5$, the excitations remain predominantly metal-centered but become strongly polarized toward the W center owing to symmetry breaking in the heterodimer. S_1 retains largely $\pi^*_\perp \rightarrow \sigma^*$ character, whereas S_2 is dominated by $\delta \rightarrow \sigma^*$; T_3 remains primarily $\sigma \rightarrow \sigma^*$.

NTOs were additionally evaluated along the LIIC connecting the FC region with the bond-cleaved triplet biradical geometry to verify the state-tracking discussed in the main text. Representative geometries on the negative-LIIC (left of FC), FC, and positive-LIIC (bond-cleavage) sides are shown below for both systems.

S10.1 NTOs Along the LIIC for $(Fv)Ru_2(CO)_4$

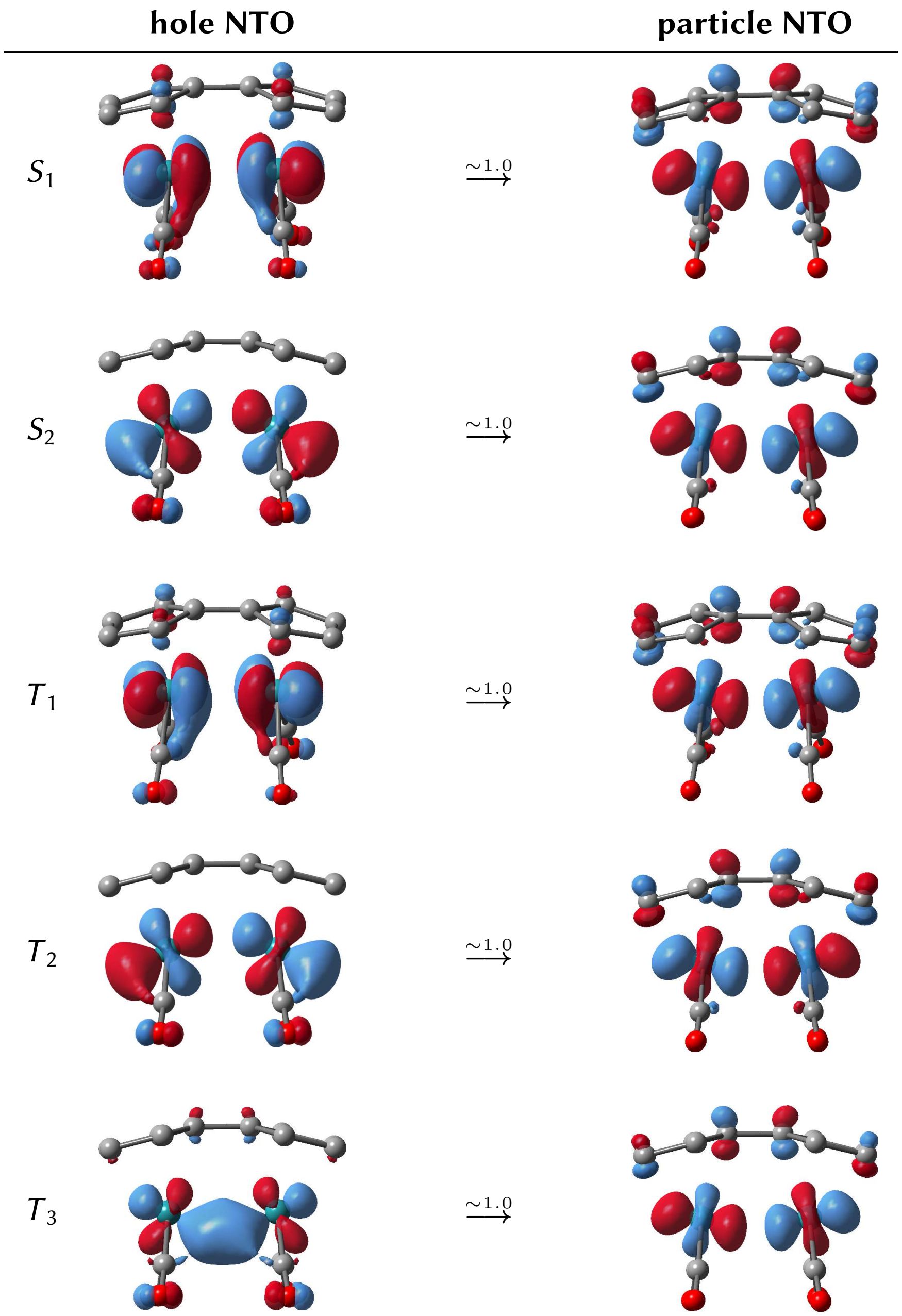

Figure S16. Dominant NTO hole–particle pairs for selected singlet and triplet excited states of $(Fv)Ru_2(CO)_4$ at a representative geometry on the negative-LIIC (left-of-FC) side. Isosurfaces are plotted at ±0.04 a.u.

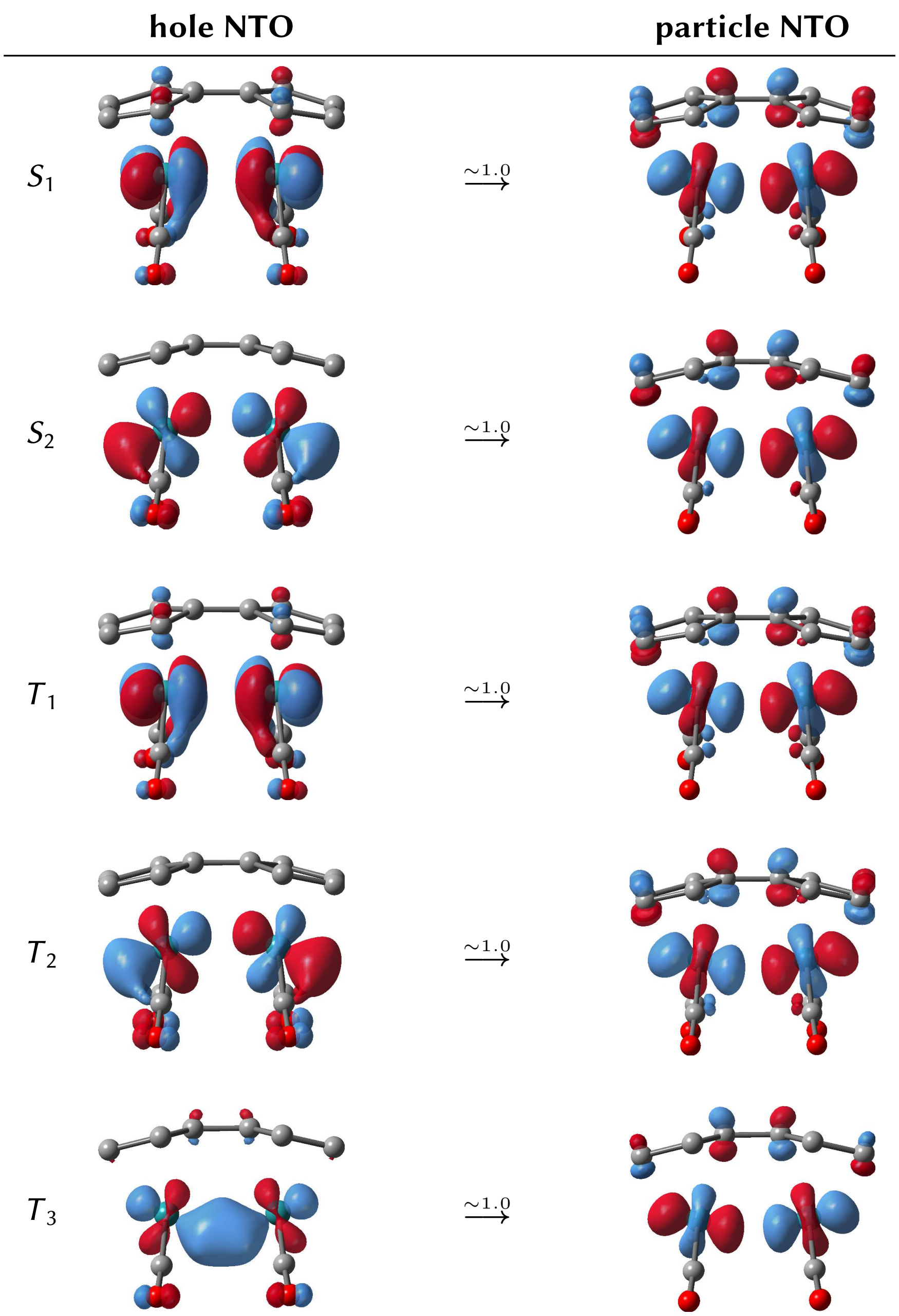

Figure S17. Dominant NTO hole–particle pairs for $(Fv)Ru_2(CO)_4$ at the FC geometry. Isosurfaces are plotted at ± 0.04 a.u.

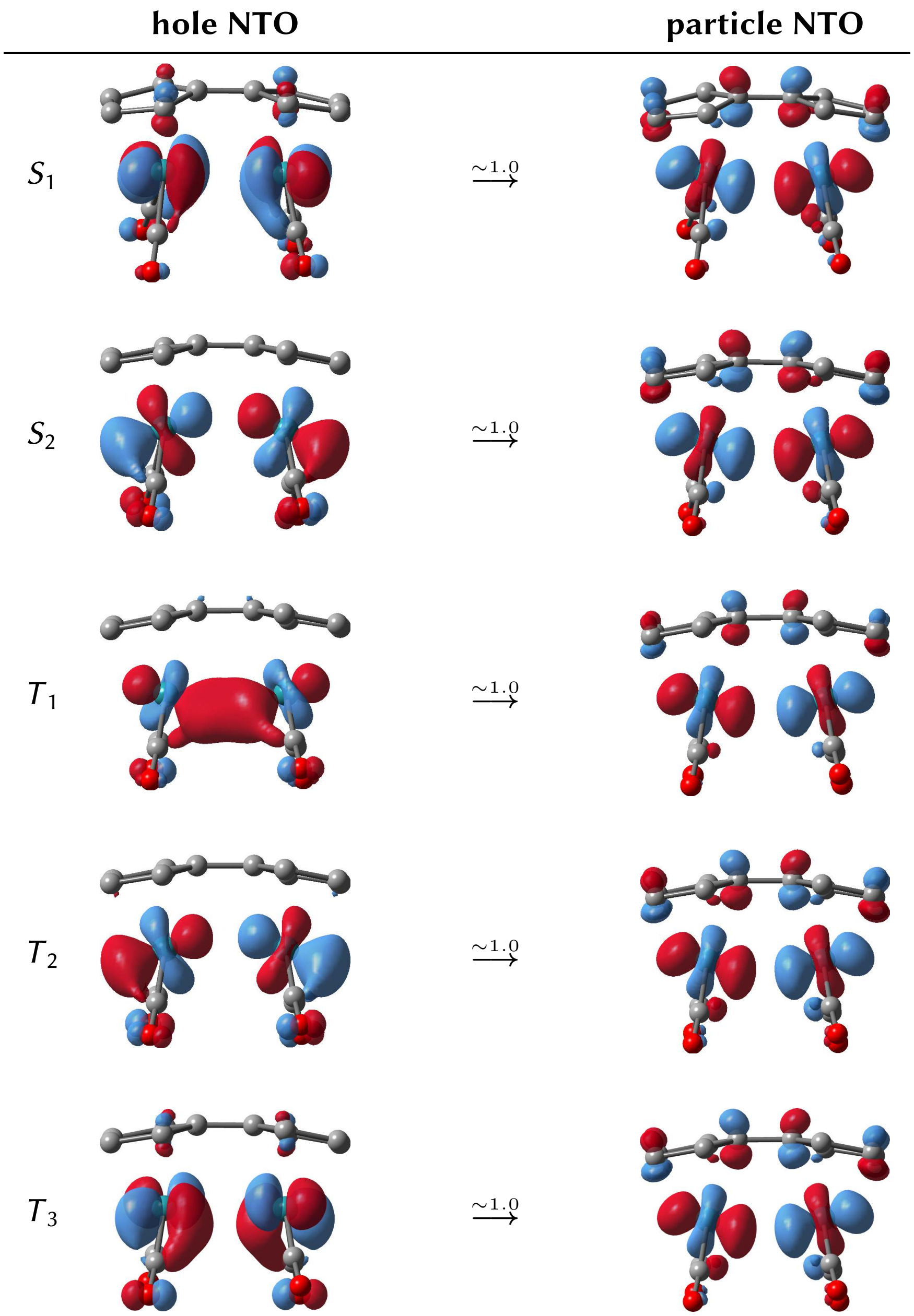

Figure S18. Dominant NTO hole–particle pairs for $(Fv)Ru_2(CO)_4$ at a representative geometry on the positive-LIIC (bond-cleavage) side. Isosurfaces are plotted at ±0.04 a.u.

S10.2 NTOs Along the LIIC for (Fv)OsW(CO)$_5$

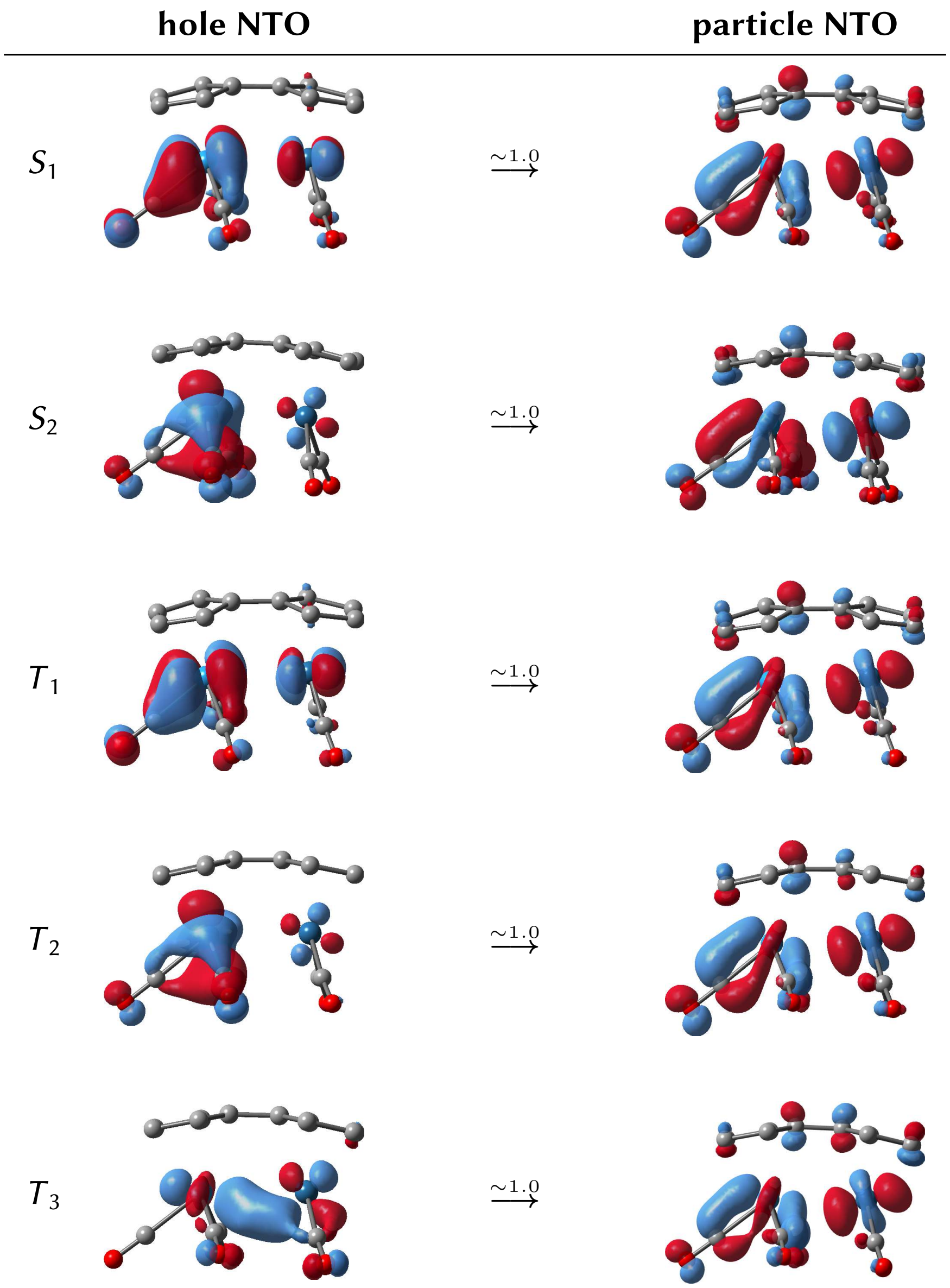

Figure S19. Dominant NTO hole–particle pairs for selected singlet and triplet excited states of (Fv)OsW(CO)$_5$ at a representative geometry on the negative-LIIC (left-of-FC) side. Isosurfaces are plotted at $\pm$0.04 a.u.

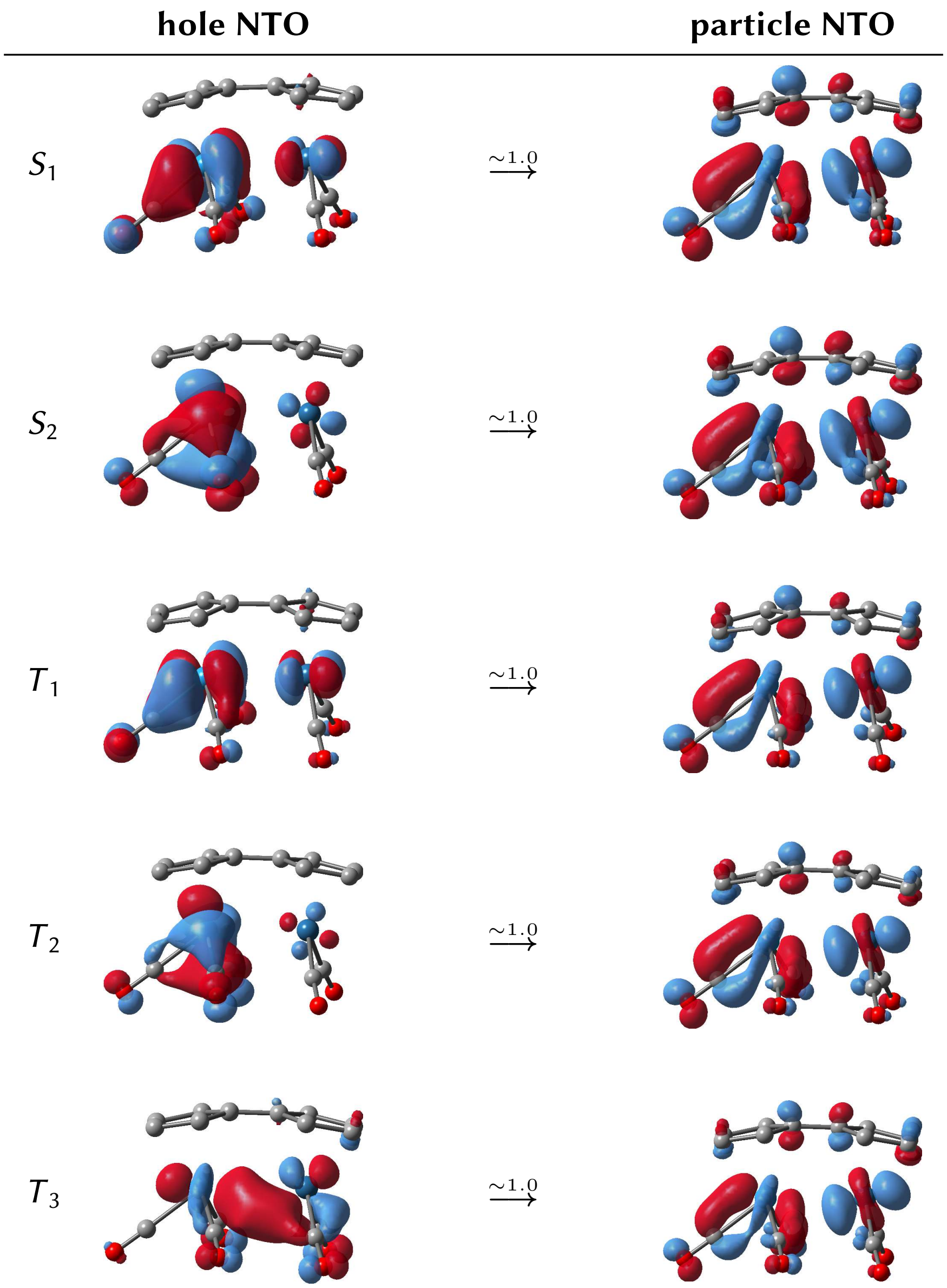

Figure S20. Dominant NTO hole–particle pairs for $(Fv)OsW(CO)_5$ at the FC geometry. Isosurfaces are plotted at ± 0.04 a.u.

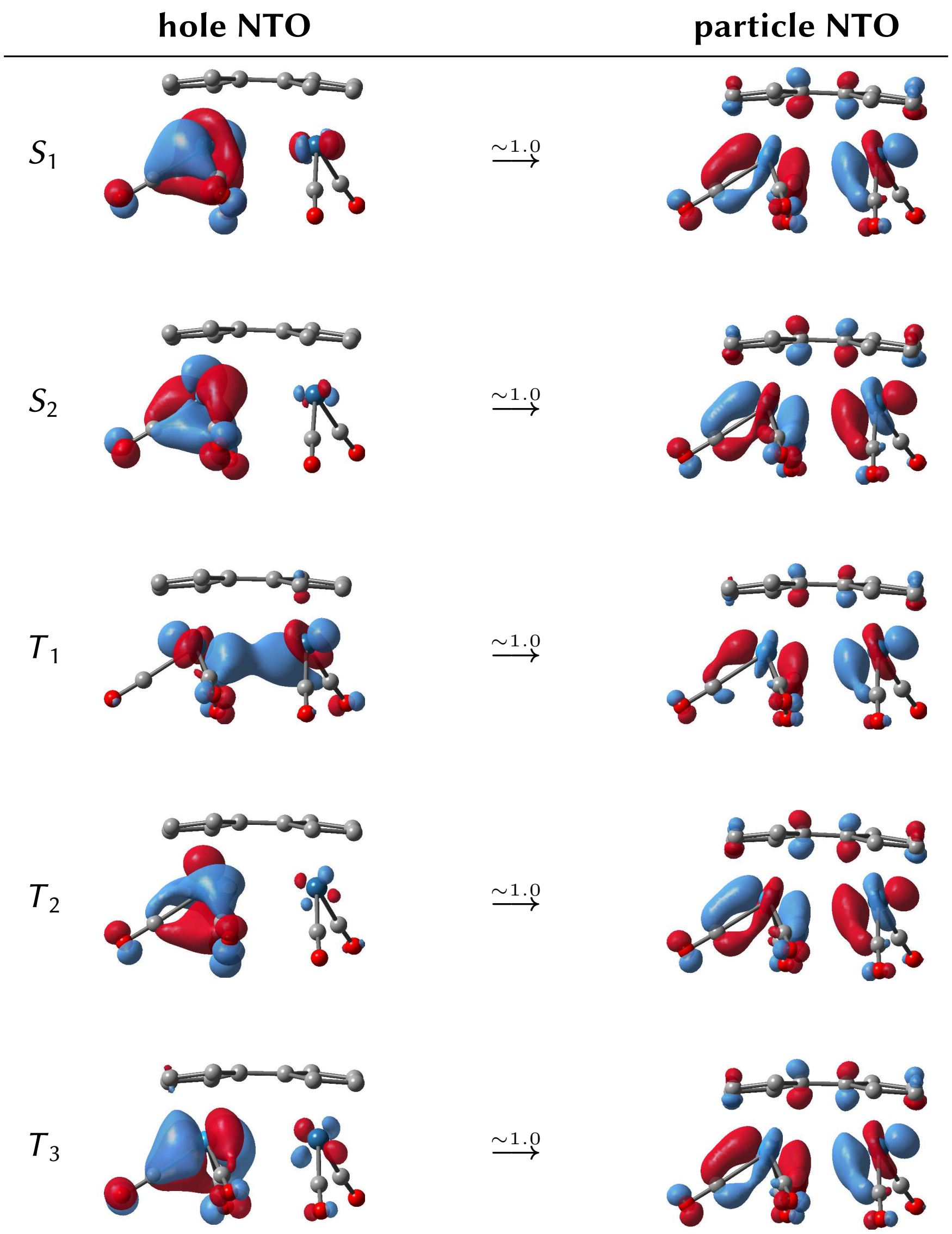

Figure S21. Dominant NTO hole–particle pairs for (Fv)OsW$(CO)_5$ at a representative geometry on the positive-LIIC (bond-cleavage) side. Isosurfaces are plotted at ± 0.04 a.u.

S11 Gate I: Fragment-Resolved Excited-State Character in $(Fv)Ru_2(CO)_4$ and $(Fv)OsW(CO)_5$

Fragment-resolved excited-state analysis was carried out with THEODORE to quantify the extent of localized and inter-fragment charge-transfer (CT) character in the low-lying singlet and triplet states relevant to Gate I screening. For $(Fv)Ru_2(CO)_4$ the analysis employs a symmetric $CpRu(CO)_2/CpRu(CO)_2$ partition; for $(Fv)OsW(CO)_5$ the fragments are $CpOs(CO)_2$ and $CpW(CO)_3$. These decompositions complement the NTO analysis by distinguishing symmetry-delocalized metal-centered excitations from polarized or partially CT states.

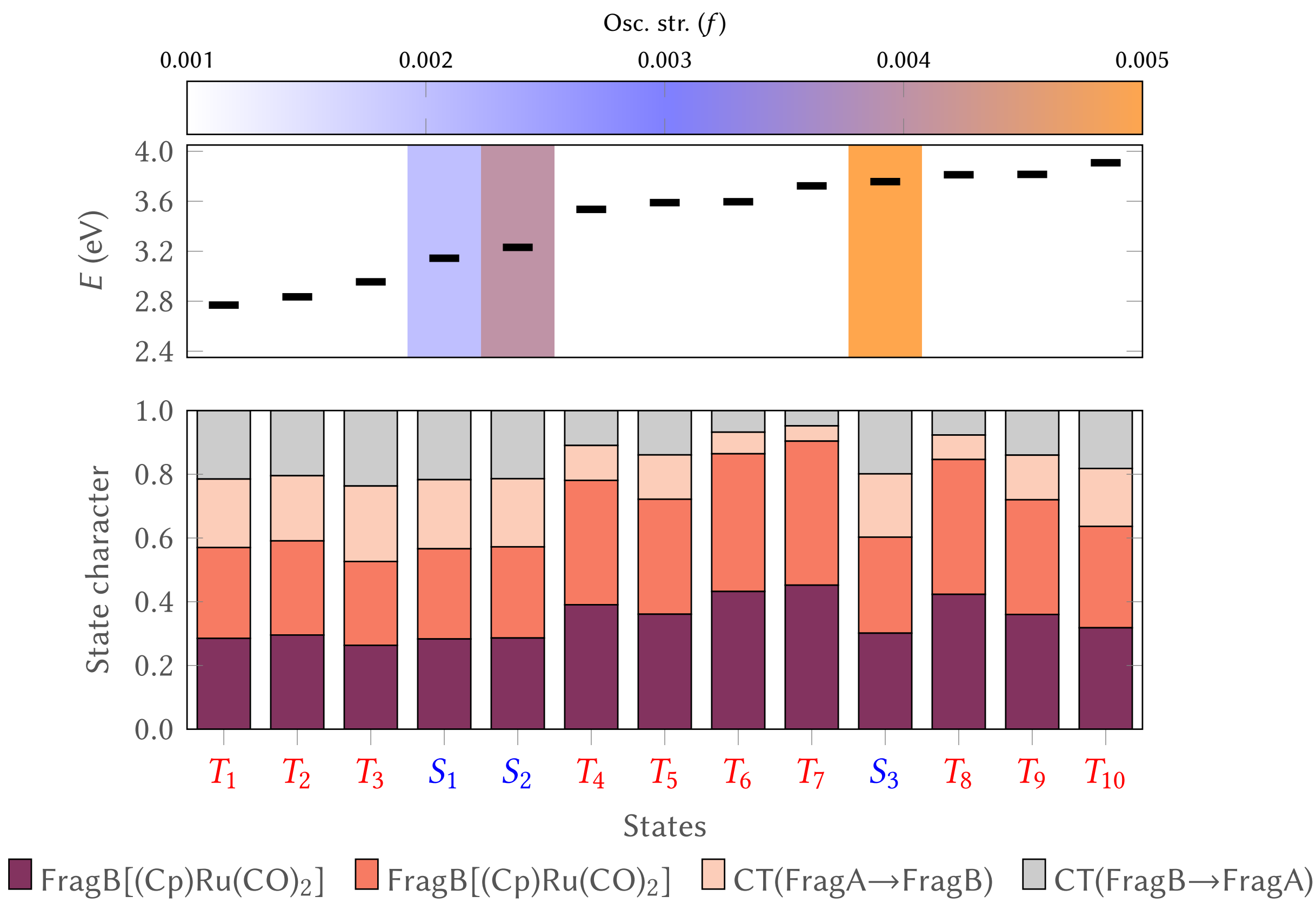

Figure S22. Fragment-resolved excited-state decomposition for $(Fv)Ru_2(CO)_4$ at the FC geometry ($CpRu(CO)_2/CpRu(CO)_2$ partition). Bar plots show relative contributions of localized and CT excitation character for the low-lying singlet and triplet states. The data confirm that the relevant excitations are predominantly metal-centered and symmetry-delocalized across the bimetallic core.

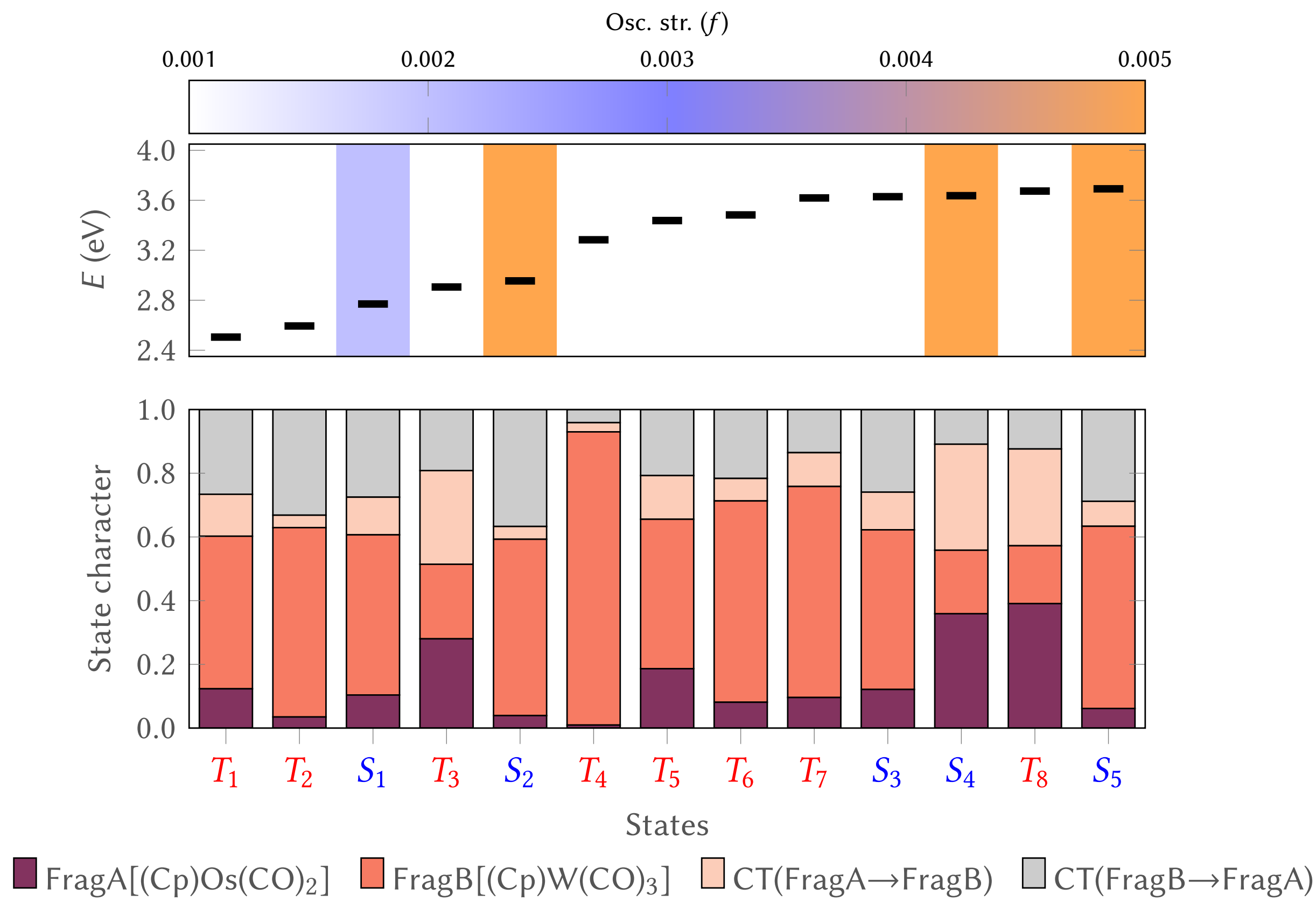

Figure S23. Fragment-resolved excited-state decomposition for $(Fv)OsW(CO)_5$ at the FC geometry ($CpOs(CO)_2/CpW(CO)_3$ partition). Bar plots show relative contributions of localized and CT excitation character. The analysis reveals predominantly metal-centered excitations with stronger localization toward the W-containing fragment, consistent with symmetry breaking in the heterobimetal.

S12 Gate I: Benchmarking TDDFT/TDA Against XMS-CASPT2 for the Low-Lying Excitation Manifold of $(Fv)OsW(CO)_5$

To assess the reliability of the TDDFT/TDA protocol employed in Gate I, we benchmarked the vertical excitation manifold of $(Fv)OsW(CO)_5$ at the ^{1}LEI Franck–Condon (FC) geometry against extended multi-state complete active space second-order perturbation theory (XMS-CASPT2). For the benchmark system $(Fv)Ru_2(CO)_4$, the corresponding comparison has been reported previously, demonstrating similarly good agreement.[S23]

XMS-CASPT2 calculations were performed with OpenMolcas (version 23.10)[S24] using a CASSCF(10,10) reference with state averaging over 5 singlet and 5 triplet roots, with ANO-R2 basis sets on Os and W, ANO-R1 on C and O, and ANO-R0 on H, and scalar-relativistic effects treated with the exact two-component (X2C) Hamiltonian. The ionization-potential–electron-affinity (IPEA) shift was set to zero (i.e., the standard IPEA correction was not applied), and an imaginary level shift of 0.2 a.u. was applied to mitigate intruder states. TDDFT/TDA calculations were carried out at the PBE0-D4/def2-TZVPP/ZORA level, consistent with the protocol employed throughout the main text (Section S2).

Table S5: Vertical excitation energies (eV) and oscillator strengths (f) of $(Fv)OsW(CO)_5$ at the ^{1}LEI FC geometry, computed with TDDFT/TDA (PBE0-D4/def2-TZVPP/ZORA) and XMS-CASPT2(10,10)/ANO-R (IPEA = 0.0). States are matched by dominant excitation character rather than by adiabatic index.

State	Character	TDDFT/TDA	f (TDDFT/TDA)	XMS-CASPT2	f (XMS-CASPT2)
T_1	$\pi^*_\perp \to \sigma^*$	2.51	—	2.40	—
T_2	δ(nb) $\to \sigma^*$	2.59	—	2.68	—
S_1	$\pi^*_\perp \to \sigma^*$	2.77	2.0×10^{-3}	2.51	1.9×10^{-2}
T_3 (T_{iso})	$\sigma \to \sigma^*$	2.91	—	2.95	—
S_2	δ(nb) $\to \sigma^*$	2.96	5.0×10^{-3}	2.92	1.0×10^{-2}

Both methods assign the third triplet (T_3) at the FC geometry as the $\sigma \to \sigma^*$ state, i.e., the isomerizing triplet T_{iso}; nb = nonbonding.

The two methods show good agreement across the low-lying manifold (Table S5), with a mean absolute deviation of 0.11 eV (maximum 0.26 eV, for S_1) and only a small systematic overestimation by TDDFT/TDA (mean signed deviation +0.06 eV). Crucially, both identify the same $\sigma \to \sigma^*$ state as the isomerizing triplet T_{iso} (T_3) and place the bright δ(nb) $\to \sigma^*$ singlet S_2 essentially degenerate with it (S_2–T_3 separation +0.05 eV at TDDFT/TDA vs. −0.03 eV at XMS-CASPT2). Thus, the FC-proximal near-degeneracy underlying the Gate I design criterion is consistently reproduced in both energetic placement and state character.

The residual deviations are modest and largely state-specific. Both methods agree on the character of all five low-lying states; the largest discrepancy is for S_1 (0.26 eV), while the remaining four states differ by no more than 0.11 eV. XMS-CASPT2 predicts somewhat smaller

singlet–triplet splittings within each excitation family (S_1–T_1: 0.11 vs. 0.26 eV; S_2–T_2: 0.24 vs. 0.37 eV for XMS-CASPT2 and TDDFT/TDA, respectively). Although the absolute oscillator strengths differ, both methods predict that the low-lying singlets are only weakly allowed ($f \lesssim 2 \times 10^{-2}$), the qualitative feature relevant to near-visible photocharging. These quantitative differences do not alter the mechanistic conclusions of Gate I, which depend on the proximity of the bright singlet manifold to $T_{\rm iso}$ rather than on the precise ordering or brightness of individual states.

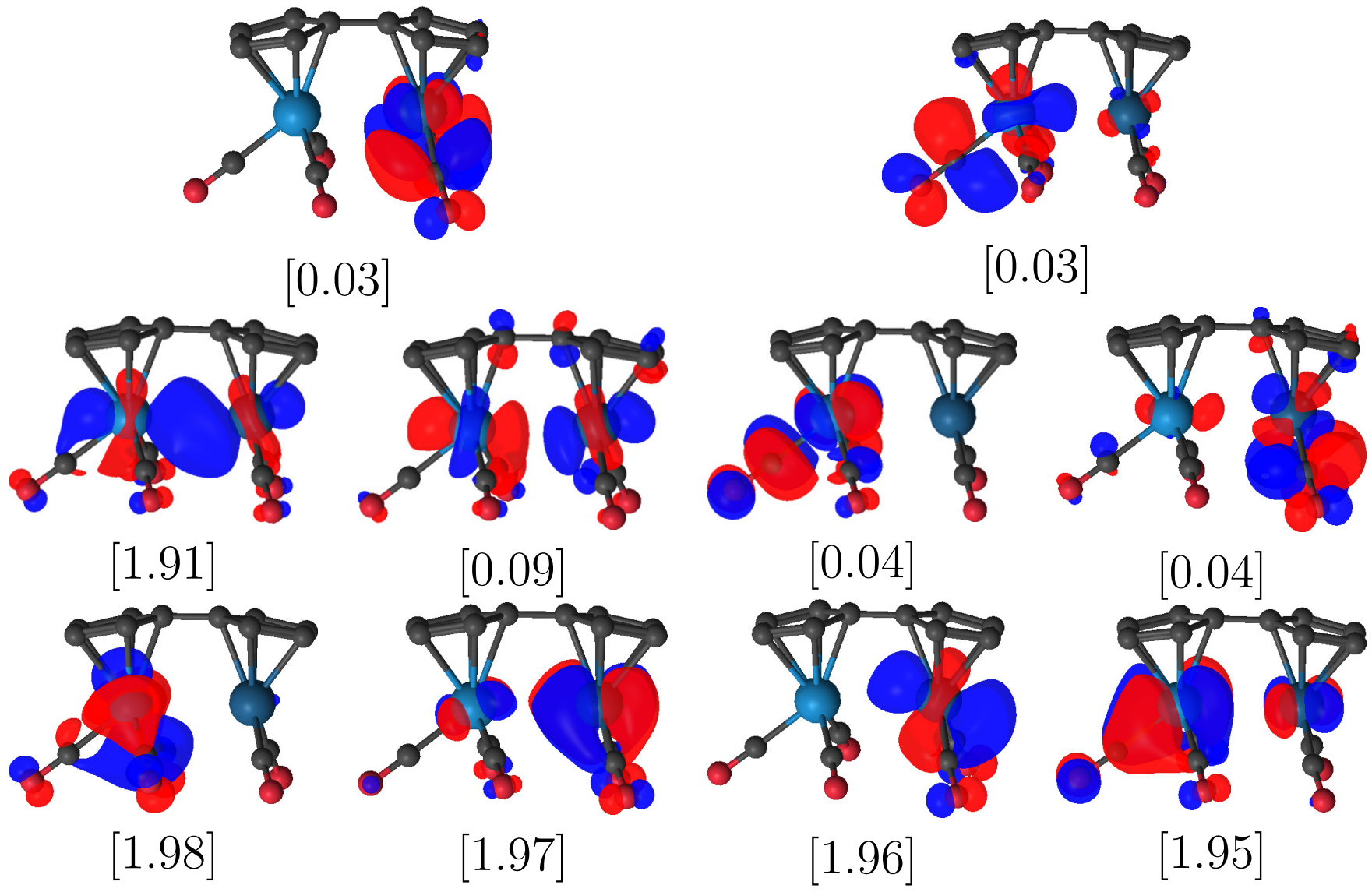

Figure S24. CASSCF(10,10) active-space natural orbitals of $(Fv)OsW(CO)_5$ at the ^{1}LEI Franck–Condon geometry, with their XMS-CASPT2 natural-orbital occupation numbers given in brackets. Isosurfaces are shown at ±0.05 a.u. (red/blue denote opposite phases). The occupations cluster near the closed-shell limits (≈1.95–1.98 for the well-occupied set and ≈0.03–0.09 for the virtual set), with only the M—M′ σ/σ^* pair showing appreciable fractional occupation (1.91/0.09). This near-integer occupation pattern confirms a predominantly single-reference ground state in the Franck–Condon region, supporting the use of the single-reference TDDFT/TDA protocol for Gate I screening.

Finally, the benchmark also addresses the possibility of spuriously low charge-transfer (CT) states, a known failure mode of TDDFT.[S25] No such states appear in the low-lying manifold. Instead, the relevant excitations are metal-centered σ/π-manifold transitions within the bimetallic core, consistent with the σ/π excitation framework established for this family of complexes,[S23] and confirmed by the NTO and fragment-resolved analyses (Section S10 and Section S11). The absence of low-lying CT states in the XMS-CASPT2 manifold—which is free from the CT-underestimation pathology of TDDFT—independently corroborates this assignment. Moreover, the CASSCF(10,10) active-space natural orbitals and their XMS-CASPT2 occupation numbers (Figure S24) cluster near the closed-shell limits, with only the M—M′ σ/σ^* pair deviating appreciably from integer occupation (1.91/0.09); together with $\langle \hat{S}^2 \rangle_{\rm BS} \approx 0$, this confirms a predominantly single-reference ground state in the FC region and thus validates the single-reference TDDFT/TDA description. Together, these results support the use of the TDDFT/TDA protocol for evaluating the Gate I design criterion.

S13 Stability of $(Fv)OsW(CO)_5$ toward Disproportionation

While $(Fv)OsW(CO)_5$ itself has not yet been reported, a central question for its practical accessibility is whether the heterobimetallic complex is stable with respect to redistribution into the corresponding homobimetallic products. This stability can be assessed through the homodimer-scrambling equilibrium

$$2\,(Fv)OsW(CO)_5 \rightleftharpoons (Fv)Os_2(CO)_4 + (Fv)W_2(CO)_6, \tag{6}$$

for which we compute $\Delta G_{\mathrm{scramble}} = +5.7$ kcal mol^{-1} ($\Delta E_{\mathrm{scramble}} = +4.6$ kcal mol^{-1}). The positive free energy indicates that the heterobimetallic complex is thermodynamically favored over the corresponding mixture of homobimetallic products, rendering spontaneous disproportionation into $(Fv)Os_2(CO)_4$ and $(Fv)W_2(CO)_6$ unfavorable. At 298 K, this free-energy difference corresponds to an equilibrium constant of approximately 10^4 in favor of $(Fv)OsW(CO)_5$: once formed, the heterobimetallic complex constitutes the thermodynamic sink of the redistribution equilibrium. We further note that any such redistribution would require bimolecular metal–metal bond metathesis between two intact dimers, a process with no apparent low-barrier pathway and no experimental precedent among fulvalene heterobimetallic complexes, which have been isolated and handled as configurationally robust species.[S26,S27] Together with the literature precedent for stable Os−W carbonyl motifs[S28–S31] and for the selective synthesis of fulvalene heterobimetallic complexes from mixed-metal precursors discussed in the main text, the thermodynamic preference established by Eq. (6) supports the experimental accessibility of $(Fv)OsW(CO)_5$ and motivates its targeted synthesis.

References

(S1) Frisch, M. J.; Trucks, G. W.; Schlegel, H. B.; et al. Gaussian 16 Revision C.01. 2016; Gaussian Inc., Wallingford CT.

(S2) Becke, A. D. Density-Functional Thermochemistry. III. The Role of Exact Exchange. *J. Chem. Phys.* **1993**, *98*, 5648–5652.

(S3) Lee, C.; Yang, W.; Parr, R. G. Development of the Colle-Salvetti Correlation-Energy Formula into a Functional of the Electron Density. *Phys. Rev. B* **1988**, *37*, 785–789.

(S4) Grimme, S.; Djukic, J.-P. The Crucial Role of Dispersion in the Cohesion of Nonbridged Binuclear Os→Cr and Os→W Adducts. *Inorg. Chem.* **2010**, *49*, 2911–2919.

(S5) Andrae, D.; Häußermann, U.; Dolg, M.; Stoll, H.; Preuss, H. Energy-Adjusted Ab Initio Pseudopotentials for the Second and Third Row Transition Elements. *Theor. Chim. Acta* **1990**, *77*, 123–141.

(S6) Weigend, F.; Ahlrichs, R. Balanced Basis Sets of Split Valence, Triple Zeta Valence and Quadruple Zeta Valence Quality for H to Rn: Design and Assessment of Accuracy. *Phys. Chem. Chem. Phys.* **2005**, *7*, 3297–3305.

(S7) Yamaguchi, K.; Jensen, F.; Dorigo, A.; Houk, K. N. A Spin Correction Procedure for Unrestricted Hartree–Fock and Møller–Plesset Wavefunctions for Singlet Diradicals and Polyradicals. *Chemical Physics Letters* **1988**, *149*, 537–542.

(S8) Yamanaka, S.; Kawakami, T.; Nagao, H.; Yamaguchi, K. Effective Exchange Integrals for Open-Shell Species by Density Functional Methods. *Chem. Phys. Lett.* **1994**, *231*, 25–33.

(S9) Neese, F. The ORCA Program System. *WIREs Comput. Mol. Sci.* **2012**, *2*, 73–78.

(S10) Neese, F. Software Update: The ORCA Program System—Version 6.0. *WIREs Comput. Mol. Sci.* **2025**, *15*, e70019.

(S11) Adamo, C.; Barone, V. Toward Reliable Density Functional Methods without Adjustable Parameters: The PBE0 Model. *J. Chem. Phys.* **1999**, *110*, 6158–6170.

(S12) Van Wüllen, C. Molecular density functional calculations in the regular relativistic approximation: Method, application to coinage metal diatomics, hydrides, fluorides and chlorides, and comparison with first-order relativistic calculations. *J. Chem. Phys.* **1998**, *109*, 392–399.

(S13) Plasser, F. TheoDORE: A Toolbox for a Detailed and Automated Analysis of Electronic Excited State Computations. *J. Chem. Phys.* **2020**, *152*, 084108.

(S14) Kanai, Y.; Srinivasan, V.; Meier, S. K.; Vollhardt, K. P. C.; Grossman, J. C. Mechanism of Thermal Reversal of the (Fulvalene)tetracarbonyldiruthenium Photoisomerization: Toward Molecular Solar–Thermal Energy Storage. *Angew. Chem. Int. Ed.* **2010**, *49*, 8926–8929.

(S15) Grimme, S.; Hansen, A. A Practicable Real-Space Measure and Visualization of Static Electron-Correlation Effects. *Angew. Chem. Int. Ed.* **2015**, *54*, 12308–12313.

(S16) Bauer, C. A.; Hansen, A.; Grimme, S. The Fractional Occupation Number Weighted Density as a Versatile Analysis Tool for Molecules with a Complicated Electronic Structure. *Chem. Eur. J.* **2017**, *23*, 6150–6164.

(S17) Tao, J.; Perdew, J. P.; Staroverov, V. N.; Scuseria, G. E. Climbing the density functional ladder: Nonempirical meta–generalized gradient approximation designed for molecules and solids. *Phys. Rev. Lett.* **2003**, *91*, 146401.

(S18) Rolfes, J. D.; Neese, F.; Pantazis, D. A. All-electron scalar relativistic basis sets for the elements Rb–Xe. *J. Comput. Chem.* **2020**, *41*, 1842–1849.

(S19) Dachsel, H.; Harrison, R. J.; Dixon, D. A. Multireference configuration interaction calculations on cr2: Passing the one billion limit in mrci/mracpf calculations. *J. Phys. Chem. A* **1999**, *103*, 152–155.

(S20) Celani, P.; Stoll, H.; Werner, H.-J.; Knowles*, P. The CIPT2 method: Coupling of multireference configuration interaction and multi-reference perturbation theory. Application to the chromium dimer. *Mol. Phys.* **2004**, *102*, 2369–2379.

(S21) Larsson, H. R.; Zhai, H.; Umrigar, C. J.; Chan, G. K.-L. The Chromium Dimer: Closing a Chapter of Quantum Chemistry. *J. Am. Chem. Soc.* **2022**, *144*, 15932–15937.

(S22) Börjesson, K.; Ćoso, D.; Gray, V.; Grossman, J. C.; Guan, J.; Harris, C. B.; Hertkorn, N.; Hou, Z.; Kanai, Y.; Lee, D.; others Exploring the Potential of Fulvalene Dimetals as Platforms for Molecular Solar Thermal Energy Storage: Computations, Syntheses, Structures, Kinetics, and Catalysis. *Chem. Eur. J.* **2014**, *20*, 15587–15604.

(S23) Ganguly, G.; González, L. Unraveling the Photoisomerization Mechanism of Group-8 Fulvalene-Bridged Bimetallic Complexes for Molecular Solar–Thermal Energy Storage. *J. Am. Chem. Soc.* **2025**, *147*, 41855–41866.

(S24) Li Manni, G.; Fdez. Galván, I.; Alavi, A.; Aleotti, F.; Aquilante, F.; Autschbach, J.; Avagliano, D.; Baiardi, A.; Bao, J. J.; Battaglia, S.; others The OpenMolcas Web: A Community-Driven Approach to Advancing Computational Chemistry. *J. Chem. Theory Comput.* **2023**, *19*, 6933–6991.

(S25) Dreuw, A.; Head-Gordon, M. Failure of Time-Dependent Density Functional Theory for Long-Range Charge-Transfer Excited States: The Zincbacteriochlorin–Bacteriochlorin and Bacteriochlorophyll–Spheroidene Complexes. *J. Am. Chem. Soc.* **2004**, *126*, 4007–4016.

(S26) Zhu, B.; Miljanić, O. Š.; Vollhardt, K. P. C.; West, M. J. Synthesis of 2,2',3,3'-tetramethyl- and 2,2',3,3'-tetra-tert-butylfulvalene: Attractive platforms for dinuclear transition metal fragments, as exemplified by (η^5:η^5-2,2',3,3'-t-$Bu_4C_{10}H_4$)$M_2(CO)_n$ (M = Fe, Ru, Os, Mo) and first X-ray crystal structures of fulvalene diiron and diosmium complexes. *Synthesis* **2005**, *2005*, 3373–3379.

(S27) Tilset, M.; Vollhardt, K. P. C.; Boese, R. Chemistry of Dinuclear Fulvalene Complexes: Dihydrides, Zwitterions, and Ring-Slippage Complexes Derived From $FvM_2(CO)_6$ (M = Mo, W). *Organometallics* **1994**, *13*, 3146–3169.

(S28) Jiang, F.; Jenkins, H. A.; Biradha, K.; Davis, H. B.; Pomeroy, R. K.; Zaworotko, M. J. Compounds with Unbridged Dative Metal–Metal Bonds of Formula $(R_3P)_2(OC)_3OsW(CO)_5$ and Related Complexes. *Organometallics* **2000**, *19*, 5049–5062.

(S29) Nakatsuji, H.; Hada, M.; Kawashima, A. Electronic Structures of Dative Metal–Metal Bonds; Ab Initio Molecular Orbital Calculations of $(OC)_5Os–M(CO)_5$ (M = Tungsten, Chromium) in Comparison with $(OC)_5M–M(CO)_5$ (M = Rhenium, Manganese). *Inorg. Chem.* **1992**, *31*, 1740–1744.

(S30) Davis, H. B.; Einstein, F. W. B.; Glavina, P. G.; Jones, T.; Pomeroy, R. K.; Rushman, P. Complexes with Unbridged Dative Bonds between Osmium and a Group 6 Element. Structures of $(Me_3P)(OC)_4OsM(CO)_5$ (M = Chromium, Tungsten). *Organometallics* **1989**, *8*, 1030–1039.

(S31) Shipley, J. A.; Batchelor, R. J.; Einstein, F. W. B.; Pomeroy, R. K. Complexes with Unbridged Dative Bonds between Osmium and a Group 6 Element. Structures of $(OC)_{5-x}(tert\text{-}BuNC)_xOsCr(CO)_5$ ($x = 1, 2$). *Organometallics* **1991**, *10*, 3620–3629.
